# The whole body transcriptome of *Coleophora obducta* reveals important olfactory proteins

**DOI:** 10.7717/peerj.8902

**Published:** 2020-04-10

**Authors:** Dongbai Wang, Jing Tao, Pengfei Lu, Youqing Luo, Ping Hu

**Affiliations:** 1 Forestry College, Guangxi University, Nanning, Guangxi, China; 2 Xingan Vocational and Technical College, Xinganmeng, Inner Mongolia, China; 3 Beijing Key Laboratory for Forest Pest Control, Beijing Forestry University, Beijing, China

**Keywords:** Odorant binding proteins, Chemosensory proteins, Odorant receptors, Ionotropic receptors, Odorant-degrading enzymes, Sensory neuron membrane proteins, Whole body transcriptome

## Abstract

**Background:**

The tiny casebearer moth *Coleophora obducta*, an important defoliator of *Larix* spp., is a major threat to ecological security in north China. Studies have shown that *C. obducta* is strongly specific to host plants; it is unable complete its life cycle without *Larix* spp. The sex pheromones of *C. obducta* Z5-10:OH have been elucidated; and eight types of antennae sensilla, have been detected, indicating that an exploration of its olfactory proteins is necessary, due to the general lack of information on this topic.

**Methods:**

We investigated the whole body transcriptome of *C. obducta*, performed a phylogenetic analysis of its olfactory proteins and produced expression profiles of three pheromone-binding proteins (*CobdPBPs*) by qRT–PCR.

**Results:**

We identified 16 odorant binding proteins, 14 chemosensory proteins, three sensory neuron membrane proteins, six odorant degrading enzymes, five antennal esterases, 13 odorant receptors, seven ionotropic receptors and 10 gustatory receptors, including three PBPs and one odorant co-receptor. Additionally, three putative pheromone receptors, two bitter gustatory receptors and five functional ionotropic receptors were found by phylogenetic analysis. The expression profiles of three PBPs in males and females showed that all of them exhibited male-specific expression and two were expressed at significantly higher levels in males. These data provide a molecular foundation from which to explore the olfactory recognition process and may be useful in the development of a new integrated pest management strategy targeting olfactory recognition of *C. obducta*.

## Introduction

**Figure 1:**
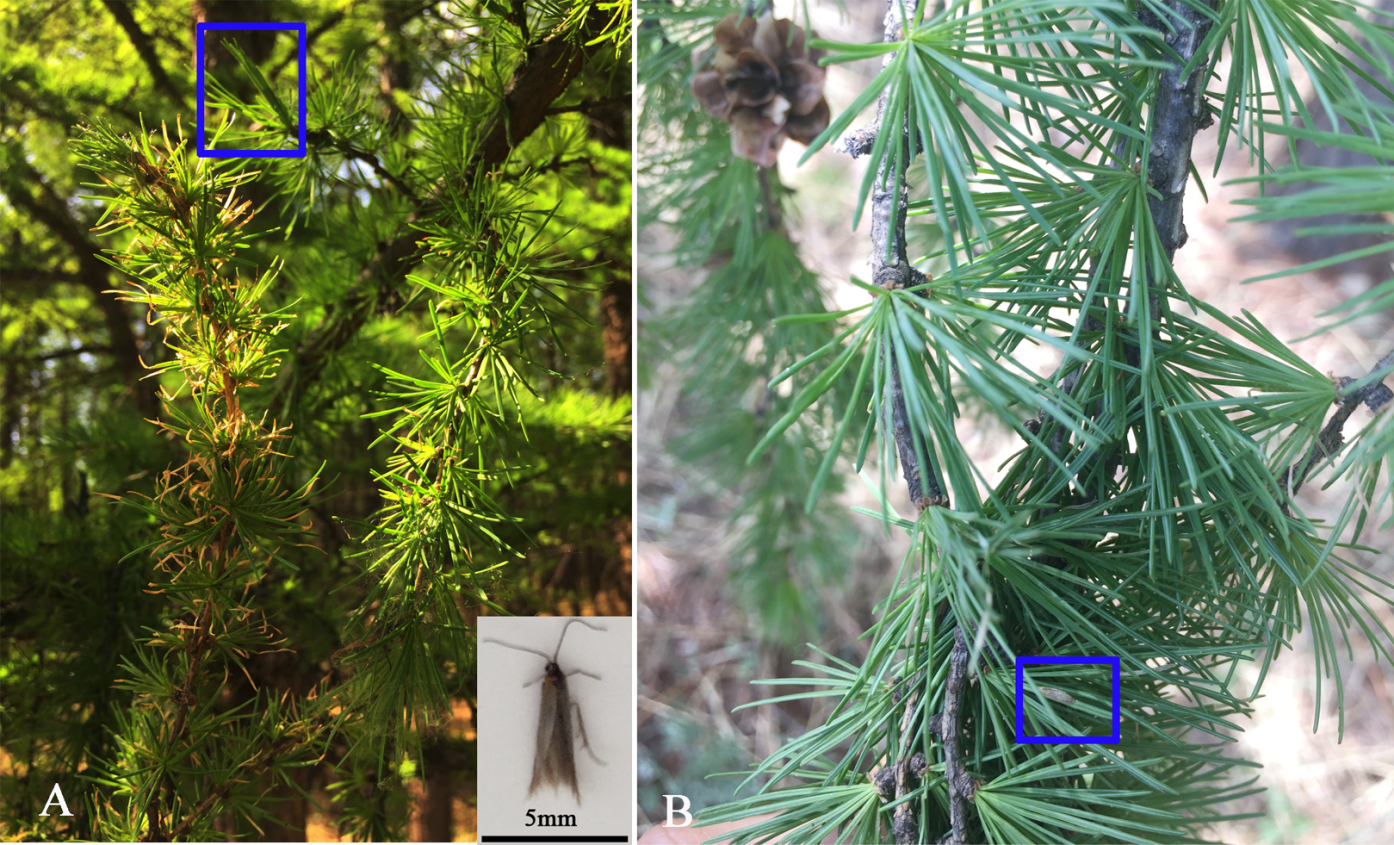
The figure of *Coleophora obducta* and damaged leaves of *Larix gmelini* (A). Old sheath on the leaf of *Larix gmelini* (B). Blue boxes in (A) and (B) are new and old sheath, respectively.

The olfactory insect-plant chemical communication system is central and significant to survival and propagation due to its essential function in growth and development e.g., eating, orientation, searching for hosts, copulation and oviposition (Jones et al., 2011; Syed & Leal, 2008; Zwiebel & Takken, 2004). To achieve integrated pest management, the molecular mechanisms of the olfactory recognition system have come under scrutiny. The tiny casebearer moth *Coleophora obducta* (Meyrick) (Lepidoptera: Coleophoridae) is an important defoliator of larch that exclusively destroys the leaves of *Larix* spp., including *Larix gmelini*, *Larix principisrupprechtii*, *Larix olgensis* and *Larix kaempferi*, which are widely distributed in far eastern Russian, Japan, Korea and Liaoning, Jilin, Heilongjiang, Inner Mongolia, Hebei and the Henan province in China. *C. obducta* is 8–10 mm long, including 2–3 mm wing over the abdomen (Fig. 1) (Li, 2003; Yang, 1984). In northeast China, especially the Greater Khingan Mountains, *L. gmelini*, which makes up half of the trees in the forest, has been damaged by *C. obducta* since 1956. From 1956 to 1990, there were four 10-year fastigium cycles (Yushan, 2004), so *C. obducta* is the main defoliator of *L. gmelini* in northeast China. The larvae remain on the leaves for the first two instars; they then produce and wear a sheath that damages larch leaves. The oldest instar larvae cause the fastest and greatest damage by eating four needles in 1 day (Bao, Yang & Zhao, 1990). When there is an outbreak, the damage, which is similar to fire disastersn larch forests, seriously affects the growth and development of trees and may lead directly to the death of a large number of trees. As a result the ecological value of a forest is weakened. Because the local tree species, *L. gmelini* has important ecological value in northeast China, *C. obducta* has been deemed the main menace to north China’s ecological security, especially in the Greater Khingan Mountains (Bao, Yang & Zhao, 1990) (Fig. 1).

After a long period of coevolution between insects and plants, a complex chemical information network has been gradually established. These chemicals are responsible for many insect behaviors and physiological reactions, coordinating utrition among plants, herbivorous insects and their natural enemies (Du & Yan, 1994). Previous studies have shown that *C. obducta* cannot complete its lifecycle without *Larix* spp., due to its strong specificity for host plants (Shu et al., 2003), so the chemical communication and messaging between *C. obducta* and larch trees is interesting. Eight types of *C. obducta* sensillum, placodea, basiconica, coeloconica, styloconica, trichodea, squamiformia, furcatea of sensillum and Bohm bristles, have been found in antennae; they function as chemoreceptors and as gustatory and mechanosensory receptors (Yang, Yan & Liu, 2009). Z5-10:OH (*cis*-5-decene-1-alcohol), the sex pheromone of *C. obducta*, was shown to be a strong attractant for male moths. Z5-10:AC and Z5-12:OH were also found to be strong and weak inhibitors, respectively, of this pheromone (Clearwater et al., 1991). An optimal dose of 100 µg of pheromone was developed for field monitoring (Chu & Zhang, 1995); however, the trapping effect is not efficient at high population densities (Chen et al., 2002; Liu et al., 2008). Thus, we investigated the olfactory recognition system to clarify the sex pheromone recognition process.

In the first step of olfactory recognition, the perireceptor event, mostly hydrophobic olfactory chemical molecules (pheromones and odors) are transformed into water-soluble molecules and transported from the external environment to the membranes of chemosensing neurons (Zhou, 2010). This is performed by odorant binding proteins (OBPs) and chemosensory proteins (CSPs), which are small soluble proteins that are highly concentrated in the lymph of chemosensilla (Ban et al., 2002; Fleischer et al., 2018; Kaissling, 2001; Leal et al., 2005; Pelosi et al., 2006; Vieira & Rozas, 2011; Vogt & Riddiford, 1981). Soluble OBPs have a conserved pattern of six cysteines that form three disulfide bridges (Leal, Nikonova & Peng, 1999). Pheromone binding proteins (PBPs) are members of a subfamily of OBPs (Zhou, 2010) that bind to pheromone compounds, participate in the pheromone recognition process and exhibit biased expression in antenna, such as in *Eogystia hippophaecolus, Sesamia nonagrioides* and *Helicoverpa assulta* (Glaser et al., 2013; Hu et al., 2016a; Li et al., 2015). CSPs have only four cysteines are smaller than OBPs (Pelosi et al., 2006), and bind to various odors (Ban et al., 2002; Briand et al., 2010; Jacquinjoly et al., 2001; Lartigue et al., 2002). Since the development of genomic and transcriptomic sequencing techniques, OBPs and CSPs have been widely investigated (Pelosi et al., 2018). In the insect chemosensory system, OBPs and CSPs function in the detection and recognition of environmental chemical stimuli. OBPs and CSPs also have different functions in non-sensory organs, including the solubilization of nutrients, pheromone delivery, development and insecticide resistance (Pelosi et al., 2018). OBPs are thought to be involved in the conveyance of odors to odorant receptors (ORs) for specific signal transduction of behaviorally active odors (Venthur & Zhou, 2018).

In insects, chemoreception is mediated by transmembrane receptors, including ORs, ionotropic receptors (IRs), gustatory receptors (GRs) and sensory neuron membrane proteins (SNMPs), which recognize and discriminate between different kinds of semiochemicals and environmental odors (Carraher et al., 2015; Clyne et al., 1999; Fleischer et al., 2018; Leal, 2013; Vosshall et al., 1999; Wicher, 2015). Among these, ORs have been most extensively studied. ORs transmit chemical signals through heteromeric complexes with an Orco-receptor (Orco), which functions as a nonselective cation channel (Sato et al., 2008; Wicher et al., 2008). ORs perceive most food odors (Ronderos & Smith, 2009), while pheromone receptors (PRs) bind pheromones and their complexes (Chang et al., 2015; Leal, 2013). ORs can raise the specificity and sensitivity of odorant recognition. When *CsupPR4* and *CsupPR6* were co-expressed with *CsupPBP4*, the sensitivity of the reaction with (Z)-11-hexadecenal was significantly enhanced (Chang et al., 2015). Many kinds of odors, including acids, aldehydes and even humidity, are perceived by IRs (Ronderos & Smith, 2009). GRs perceive nucleotides, sugars, amino acids, salts, CO_2_, acidic pH conditions and multifarious bitter compounds (Liman, Zhang & Montell, 2014). The main function of ORs and GRs is as ligand-gated ion channels in the perception of pheromones and environmental chemicals. They are also involved in photoreception and thermosensation and they have non-sensory roles (Richard, 2015). SNMPs are part of the CD36 protein family, which participates in pheromone recognition (Vogt et al., 2009) and is conserved throughout holometabolous insects (Jiang et al., 2016; Vogt et al., 2009). Depending on the functional groups of different odors, which may be plant volatiles, pheromones, aldehydes, alcohols, or esters, degradation involves specific enzymes in odor degradation pathways, including multi-functional odorant-degrading enzymes (ODEs), pheromone degrading enzymes (PDEs) and antennal esterases (AES) and all of them are belong to carboxylesterases (CXEs). The subgroup of CXEs uses the Oakeshott classification system (Chertemps et al., 2015).

In this study, we examined the whole body transcriptome of *C. obducta*, identified olfactory proteins and evaluated the phylogenetic relationships between *C. obducta* and other species. We also explored the expression profiles of three PBPs in *C. obducta* males and females. The identified olfactory proteins provide a molecular foundation from which to explore the olfactory recognition process and to develop a new integrated pest management strategy targeting olfactory recognition in *C. obducta*.

## Materials and Methods

### Ethics statement

The tiny casebearer moth *C. obducta* is a Chinese forestry pest and collection of it is permitted by the leader of Xinganmeng forestry bureau, Yinghua Lu and member Tianhua Zhen. It is not in ‘‘List of Endangered and Protected Animals in China’’. For reduction ache and discomfort to them, all operations were implemented on the basis of ethical guidelines.

### Insect collection

Artificial rearing *C. obducta* need *L. gmelini* to feed, pupate, eclosion, oviposition, environment to mating and overwintering. We are exploring it, but it’s hard to control. So we collected spoiling *L. gmelini* braches with pupa and mature larva of *C. obducta* on leaves, putted the braches in bucket with clean water and fed in insect cage outdoors during end of May to end of June 2019 in Wuchagou, Xinganmeng, China, then collected the adult *C. obducta* from the cage during that period every day. In this way we collected hundreds of *C. obducta*, which are too tiny and not enough to construct antennal transcriptome and extract antennal RNA. All bodies of *C. obducta* were stored in RNAlater (Ambion, Austin, TX, USA), then deposited at −80 °C.

### cDNA library construction and illumina sequencing

We extracted total RNA from whole body of *C. obducta* males and females utilization TRIzol reagent (Ambion, Austin, TX, USA) and the RNeasy Plus Mini Kit (No. 74134; Qiagen, Hilden, Germany) according to the manufacturer’s instructions. NanoDrop2008 (Thermo, Waltham, MA, USA) and agarose gel electrophoresis examined density and quality of RNA. Half RNA of male and female bodies with three biological replicates were used to construct three cDNA libraries respectively. Construction cDNA libraries and Illumina sequencing of samples were implemented at Majorbio Corporation (Shanghai, China). Using TruSeq RNA Sample Preparation Kit v2-Set A (No. RS-122-2001; Illumina, San Diego, CA, USA) was to perform purification and fragmentation of mRNA samples. The first-strand cDNA was synthesized by utilization random hexamer primers, then using RNase H, dNTPs, buffer and DNA polymerase I at 16 °C for 1 h to synthesize the second-strand cDNA. After end repair, A-tailing and the ligation of adaptors, the products were amplified by PCR and quantified precisely by the Qubit DNA Br Assay Kit (Q10211; Invitrogen, Carlsbad, CA, USA). cDNA libraries were obtained after they were purified by the MinElute Gel Extraction Kit (Cat No. 28604; Qiagen, Hilden, Germany). On the HiSeq2500 platform three cDNA libraries were sequenced.

### Assembly and functional annotation

All low quality and adaptor sequences in all raw reads were removed by Trimmomatic (http://www.usadellab.org/cms/index.php?page=trimmomatic) to get clean reads. Clean reads assembly was implemented by Trinity (Version: r2019-07-31) with the default parameters. The largest alternative splicing variants in the Trinity results were unigenes. The annotation of unigenes was in six databases which include NCBI non-redundant protein sequences (Nr), Protein family (Pfam), Clusters of Orthologous Groups of proteins (COG), Swiss-Prot, Kyoto Encyclopedia of Genes and Genomes (KEGG) and Gene Ontology (GO). We performed search against the Nr database (http://www.ncbi.nlm.nih.gov/genbank/), Swiss-Prot (http://www.uniprot.org/) and COG (http://www.ncbi.nlm.nih.gov/COG/) with an *E* value cutoff of 1.0E−5 in Diamond (v0.8.37.99) to annotate and classify putative protein sequences. KEGG database (http://www.genome.jp/kegg/pathway.html) were searched by Kobas (2.1.1) with default parameters. Pfam (http://pfam.sanger.ac.uk/) were searched by HMMER 3.0 package (Finn, Clements & Eddy, 2011) with default parameters. BLAST2GO was used to obtain Gene Ontology (GO) annotation of assembled unigenes with an default parameters (Götz et al., 2008). Using the NCBI ORF Finder tool (http://www.ncbi.nlm.nih.gov/gorf/gorf.html) explored the longest complete open reading frames (ORFs) of unigenes. The FPKM (fragments per kilobase per million reads) represent expression levels (Mortazavi et al., 2008), which was calculated by RSEM (RNA-Seq by Expectation–Maximization) (Version: v1.2.6) with default parameters (Bo & Dewey, 2011).

### Identification of chemosensory genes

Candidate unigenes involved in *C. obducta* olfaction from Nr database were identified by Diamond (v0.8.37.99) based on the available sequences of OBPs, CSPs, SNMPs, ODEs, ORs, GRs and IRs proteins from insecta species. All putative OBPs, CSPs, SNMPs, ORs, GRs, IRs and ODEs were examined by tBLASTn online manually to assess the Diamond results. In File S1, the nucleic acid sequences of all chemosensory genes were listed.

### Sequence and phylogenetic analysis

The N-terminal signal peptides of OBPs and PBPs were checked by SignalP4.0 (http://www.cbs.dtu.dk/services/SignalP/). All sequences to construct phylogenetic tree except *C. obducta* were obtained from NCBI protein database. Utilization Muscle method implemented in Mega v6.0 software package carried out amino acid sequence alignment (Tamura et al., 2011). The phylogenetic trees were constructed using the neighbor-joining (NJ) method (Saitou & Nei, 1987) with a pairwise deletion of gaps and *P*-distances model implemented in Mega v6.0 and colored in FigTree (Version 1.4.2). The reliability of the tree structure and node support was assessed by bootstrap analysis with 1,000 replicates. Considering higher reliability of tree, we eliminated binding proteins with less than 122 amino acids and membrane proteins with less than 333 amino acids. The phylogenetic tree of *OBPs* was based on two amino acid sequences of GOBPs, 9 OBPs (except *CobdOBP1* and 2) and 3 PBPs of *C. obducta*, all Lepidoptera PBPs, 15 of *Apis mellifera*, 23 of *Bombyx mori*, 28 of *Tribolium castaneum*, 21 of *Dendrolimus kikuchii*, 11 of *Heliothis virescens* and 14 of *C. suppressalis*. CSPs tree was based on seven of *C. obducta* (except *CobdCSP1*, *4, 7, 8, 10, 11, 12*) 20 of *D. melanogaster*, 17 of *T. castaneum*, 15 of *E. hippophaecolus*, 14 of *Ostrinia furnacali* and 14 of *D. kikuchii*. The phylogenetic analyses of ORs were based on six ORs of *C. obducta* (except *CobdOR2, 5, 6, 7, 8, 9, 10*), 33 of *D. kikuchii*, 4 Lepidoptera PRs, 17 of *Tenebrio molitor*, 7 of *D. mauritiana, 41 of Manduca sexta* and all 39 of insect Orco. ODEs tree were based on all ODEs and CXEs of *C. obducta* (except *CobdCXE4*) and all known CXEs of *Plodia interpunctella* (Liu et al., 2019), *Ectropis oblique* (Sun et al., 2017b), *Spodoptera littoralis* (Durand et al., 2010) and Chertemps et al. (2015). IRs tree was based on all IRs of *C. obducta*, 35 of *D. melanogaster*, 7 of *E. hippophaecolus*, 3 of *H. armigera* and IRs of *M. sexta, B. mori, D. plexippus* and *H. Melpomene* (Schooten et al., 2016). GRs tree was based on five GRs of *C. obduct* (except *CobdGR1, 2, 3, 64, 43a*), 30 of *D. melanogaster*, 7 of *B. mori* and Xu used in *H. armigera* (Xu et al., 2016). SNMPs tree was constructed with 3 SNMPs of *C. obducta* and all known insect SNMPs. Accession number of all chemosensory protein sequences obtained from NCBI protein database in phylogenetic tree without reference was listed in File S2.

### Expression analysis by fluorescence quantitative real-time PCR

Fluorescence quantitative real-time PCR was performed to verify the expression of candidate chemosensory genes. Total RNA of whole body of males and females were extracted following the methods described above. cDNA was synthesized from total RNA using the PrimeScriptRT Reagent Kit with gDNA Eraser to remove gDNA (No. RR047A; TaKaRa, Shiga, Japan). Gene-specific primers were designed using Primer3 (http://bioinfo.ut.ee/primer3-0.4.0/) (File S3). *Lymantria dispar* β-actin was set as reference gene (File S3). The fluorescence quantitative real-time PCR (qRT–PCR) analysis was conducted using the Bio-Rad CFX96 PCR System (Hercules, CA, USA). SYBRPremix ExTaq™ II (No. RR820A; TaKaRa, Shiga, Japan) was used for the PCR reaction under a three-step amplification. Each PCR reaction was conducted in a 25 ml reaction mixture containing 12.5 µl of SYBR Premix Ex Taq II, one ml of each primer (10 mM), two µl of sample cDNA (2.5 ng of RNA) and 8.5 µl of dH_2_O (sterile distilled water). The qRT–PCR cycling parameters were as follows: 95 °C for 30 s, followed by 40 cycles of 95 °C for 5 s, 60 °C for 30 s and 65 °C to 95 °C in increments of 0.5 °C for 5 s to generate the melting curves. To examine reproducibility, each qRT–PCR reaction for each tissue was performed in three biological replicates and three technical replicates, in which each biological replication was with 20 individuals. Negative controls without either template were included in each experiment. Bio-Rad CFX Manager (version 3.1.1517.0823) was used to normalize expression based on ΔΔCt values, with *CobdPBP3* of male in analyze mode as control sample and the 2^−ΔΔCT^ method was used (the amplification efficiency for three genes was equal to 100%) (Livak & Schmittgen, 2001). A Chi-square test was using to compare the expression level of male and female adult in SPSS Statistics 22.0. Values are presented as means ± SE.

## Results

### Transcriptome sequencing and sequence assembly

**Figure 2:**
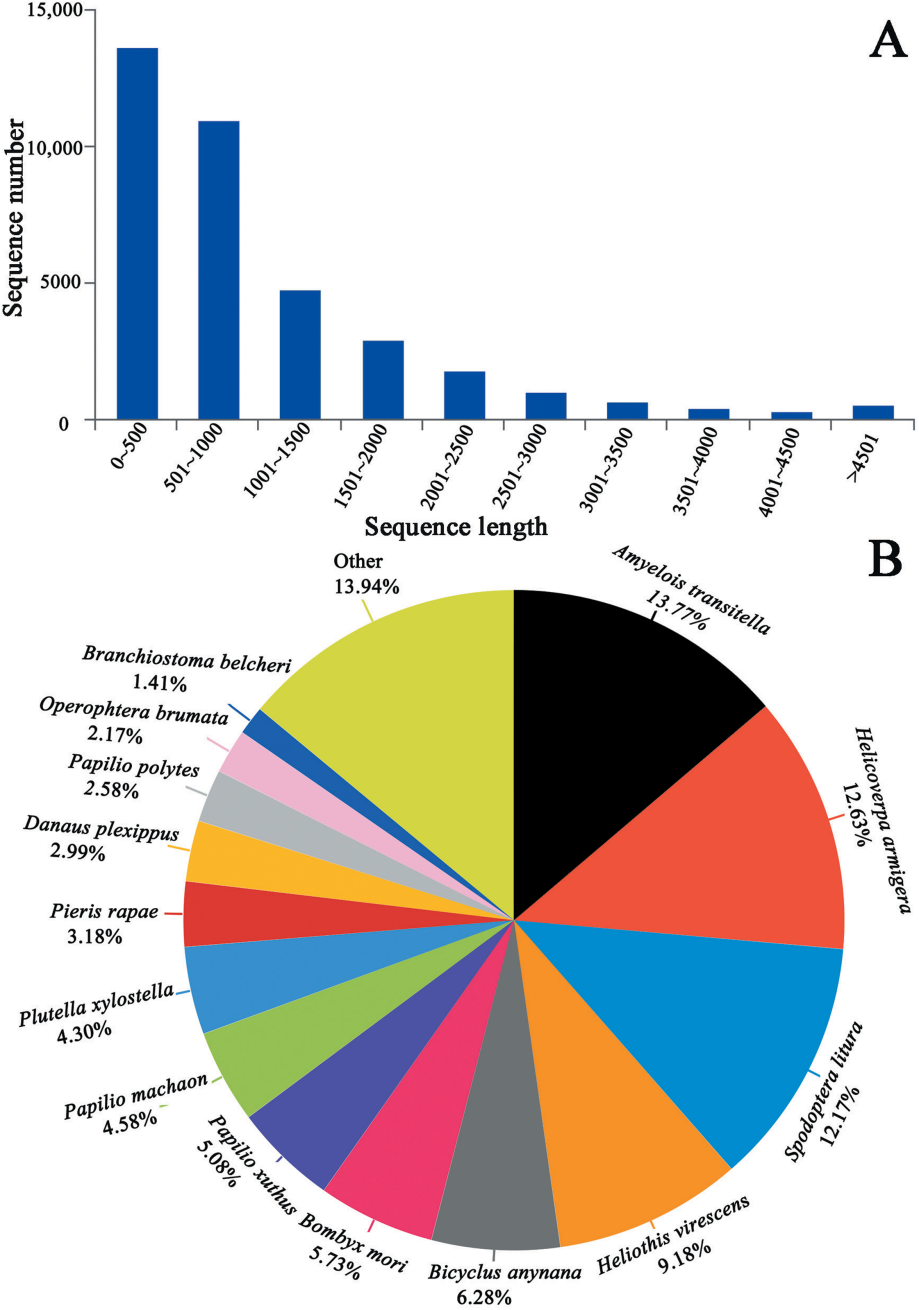
Length distribution of unigene and BLASTx unigenes with other species in the whole body transcriptome of *C. obducta* (A) length distribution of unigenes; (B) BLASTx analysis of identified unigenes with known homologs from other species.

We generated 44.07, 48.57 and 43.37 million clean reads from cDNA libraries of three biological repeats of *C. obducta* whole body (half males and females). The q20 quality scores were 97.71%, 97.55% and 97.45% respectively. The q30 quality scores were 93.84%, 93.48% and 93.27%, respectively (Table 1). After splicing and assembly, 96,657 transcripts, 52,354 unigenes, with a N50 of 1,533 bp, an average length of 900 bp and a maximal length of 19,273 bp were obtained (Table 1; Fig. 2A). The raw reads of three *C. obducta* transcriptome have been deposited in the NCBI SRA database under the accession number PRJNA587422.

**Table 1:** Number and length of unigenes.

Quality indexs	Transcriptome 1	Transcriptome 2	Transcriptome 3
Raw reads	44,539,192	49,142,628	43,896,156
Clean reads	44,072,110	48,571,928	43,368,300
Q20 (%)	97.71	97.55	97.45
Q30 (%)	93.84	93.48	93.27
GC content (%)	47.83	47.64	48.11
Total transcripts number	96,657		
Total unigenes number	52,354		
Largest length (bp)	19,273		
Average length (bp)	900		
N50	1,533		

### Homology analysis and gene ontology annotation

**Figure 3:**
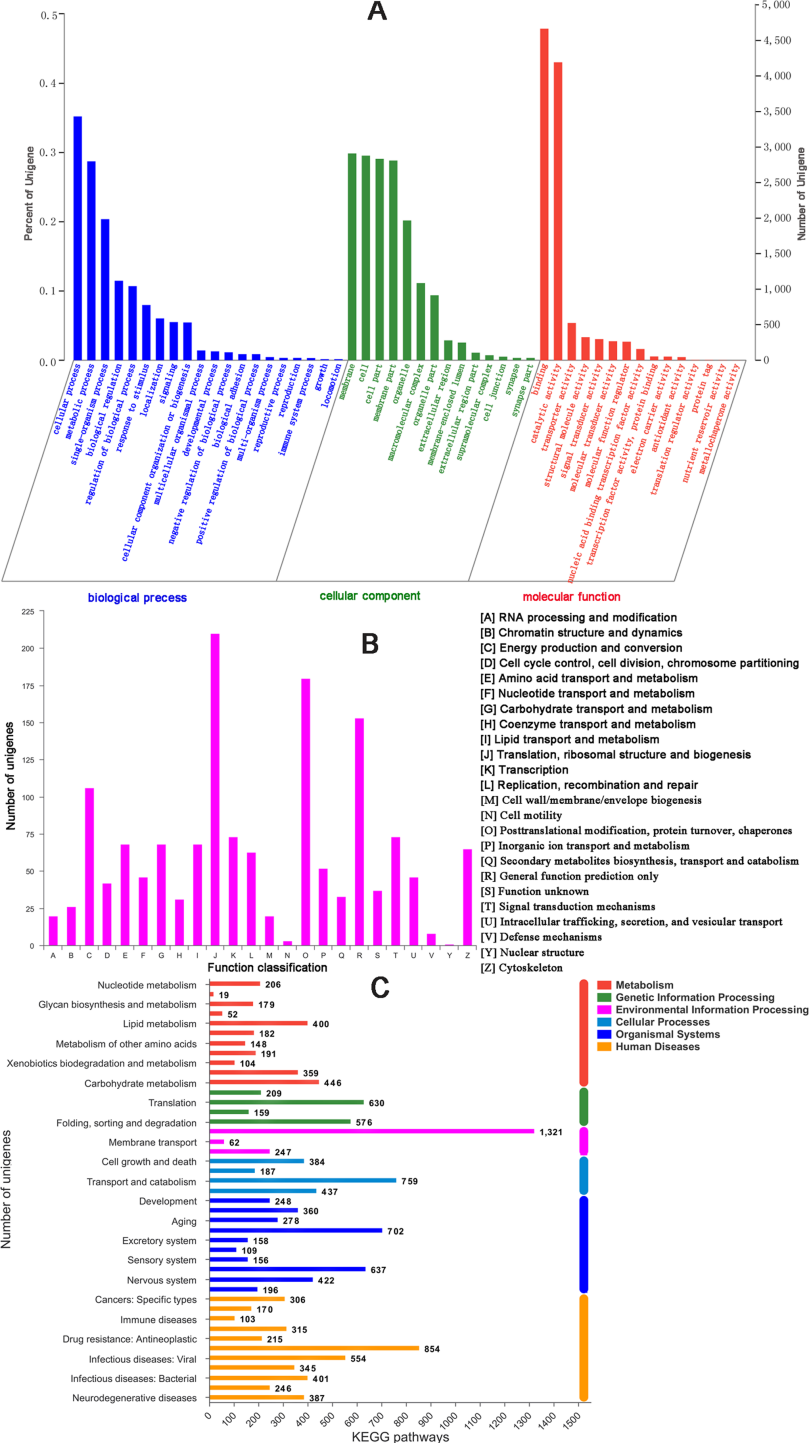
GO gene function classification, KOG and KEGG function classification. (A) GO classification. (B) KOG function classification and (C) KEGG function classification.

There were 17,176 (46.52%) unigenes that were obtained through annotation with the Nr database (Table 2). Of those, 12,206 (33.06%) aligned to the Swiss-Prot database, 12,674 (34.32%) aligned to the protein family (Pfam) database, 9,741 (26.38%) aligned to GO database and 2,937 (7.95%) aligned to the COG database (Table 2). In all, 17,821 (48.26%) unigenes were annotated in six databases. Interestingly, 2,001, 88, 155, 4 and 9 unigenes were uniquely annotated to the Nr, Swiss-Prot, Pfam, COG and KEGG databases, respectively (Table 2). The greatest number of sequences (13.77%) matched genes from *Amyelois transitella*, followed by 12.63% from *H.armigera*, 12.17% from *Spodoptera litura*, 9.18% from *Heliothis virescens* and 6.28% from *Bicyclus anynana*. A BLASTx search found that 32.03% were similar to nine species and 13.94% of unigenes were similar to other species (Fig. 2B). There were 40,565 unigenes that were categorized as functional groups by gene ontology (GO) annotation. In the *C. obducta* transcriptome, the ontology category with the most annotations was cellular component (39.96%, 16,209 gene numbers), followed by biological process (33.32%, 13,515 gene numbers) and molecular functions (26.72%, 10,841 gene numbers). In the cellular component category, the terms membrane, cell and cell part were the most representative. In the biology process category, cellular process, metabolic process and single-organism process were the most enriched terms. Binding, catalytic activity and transporter activity were the most abundant molecular functions terms (Fig. 3A). In total, 1,492 unigenes were classified into 24 COG categories. The major COG category was with 210 unigenes relating to storage and processing (14.08%), followed by cellular processes and signaling (180 unigenes, 12.06%) and poorly characterized (153 unigenes, 10.25%) (Fig. 3B). To elucidate active biosynthesis pathways in *C. obducta*, annotation of Nr data with the Kyoto Encyclopedia of Genes and Genomes (KEGG) database discovered that 14,419 gene numbers were assigned to six main categories. The highest number of KO identifiers were involved in human diseases (2,488 unigenes), followed by organismal systems (1,927), metabolism (1,840), cellular processes (1,008), genetic information processing (368) and environmental information processing (309). Signal transduction (1,321), cancer overview (854), transport and catabolism (759), the endocrine system (702), the immune system (637) and translation (630) were largest number of KO identifiers in pathways (Fig. 3C).

**Table 2:** Functional annotation of unigenes using various public protein databases.

Annotated in databases	Number of unigenes	Percentage
Nr	17,176	46.00
SwissProt	12,206	33.06
Pfam	12,674	34.32
COG	2,937	7.95
GO	9,741	26.38
KEGG	9,192	24.89
Annotated in all databases	17,821	48.26
Annotated in at least one databases	36,925	70.50

### Nonreceptor olfactory gene families

#### Odorant binding proteins

**Figure 4:**
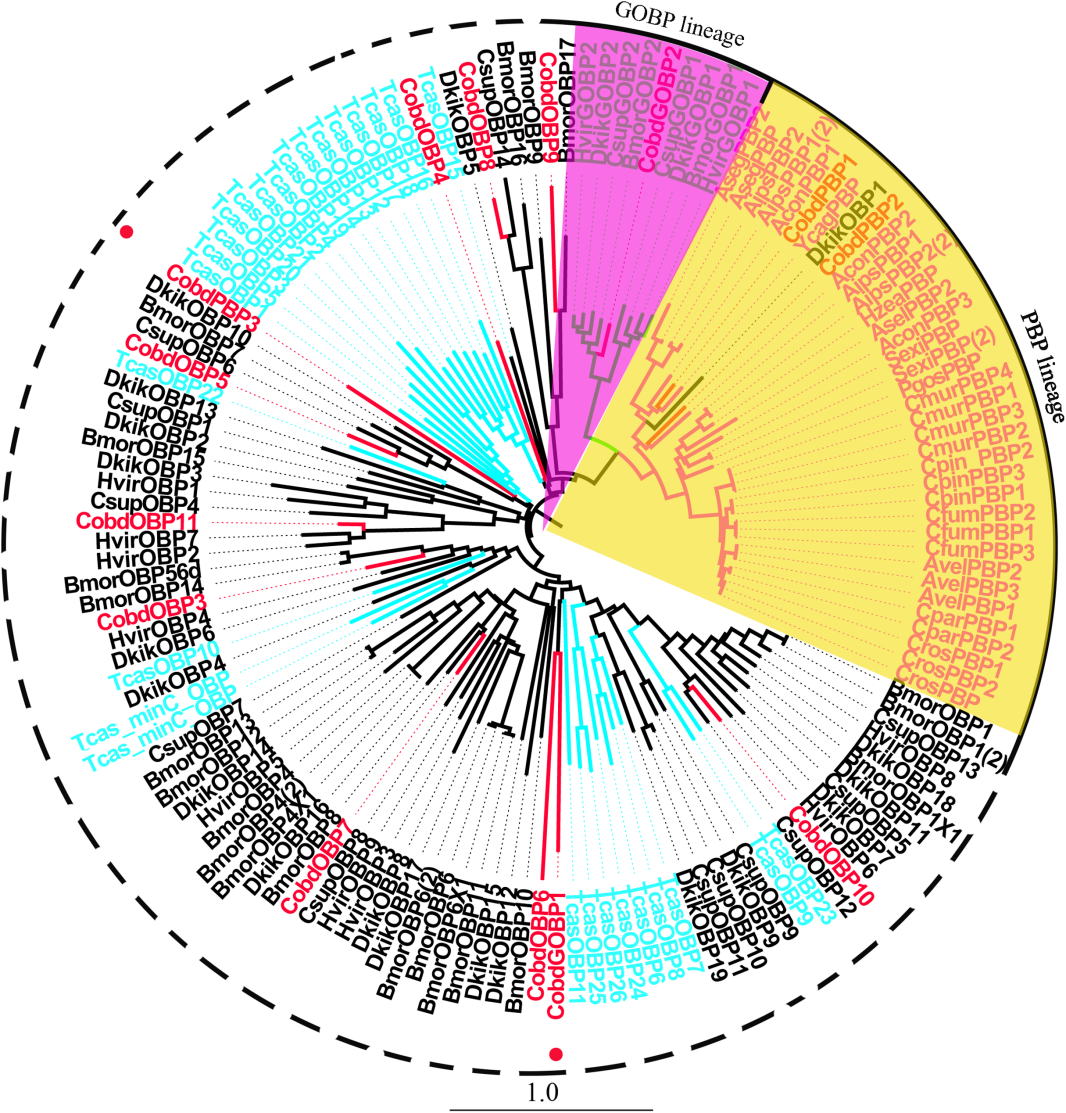
Neighbor-joining phylogenetic tree of odorant binding proteins (OBPs). The NJ phylogenetic analysis of OBPs of *C. obducta* (*CobdOBP*, red) was performed with reference OBPs of Lepidoptera (black) and Coleoptera (celeste). The yellow and purple fill area refer to PBP and GOBP lineage respectively. The scale bar represents 1.0 substitutions per site.

**Figure 5:**
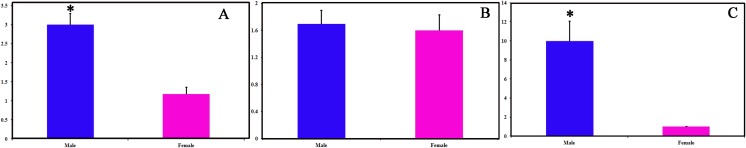
Pheromone binding protein (PBPs) transcript levels of *C. obducta* in male and female. (A) CobdPBP1; (B) CobdPBP2; (C) CobdPBP3; Actin was used as the reference gene to normalize target gene expression. The standard errors are represented by the error bars, asterisk above the bars denote significant differences at *p* < 0.05.

We identified 16 unigenes encoding putative OBPs in *C. obducta*, including two general odorant binding proteins (GOBPs) and three PBPs. Only *CobdOBP11* was a full-length gene with a complete ORF, with signal peptides and a length >400 bp (File S4). The FPKM of *CobdOBPs* showed that *CobdOBP4*, *CobdGOBP1*, *CobdGOBP2*, *CobdOBP11, CobdOBP1, CobdOBP8* and *CobdPBP1* exhibited the highest expression; the top three FPKMs of *CobdOBP4*, *CobdGOBP1* and *CobdGOBP2* were 197.37, 178.96 and 86.05, respectively. *CobdPBP1* was exhibited the highest expression with an FPKM of 23.86, followed by *CobdPBP3* and *CobdPBP2* with FPKM values of 9.08 and 5.00, respectively. In the phylogenetic tree (Fig. 4; File S2), the distinct *PBPs* clade included *CobdPBP1, CobdPBP2, DkikOBP1* and all other *PBPs;* however, *CobdPBP3* was not in the PBP lineage. The GOBP clade included *CobdGOBP2* and all other GOBPs except *CobdGOBP1*. By fluorescence quantitative real-time PCR, we verified the expression of three PBPs in adult males and females and observed higher expression levels in males than females in three PBPs. Moreover, we detected significantly higher expression levels of *CobdPBP1* and *CobdPBP3* in males than in females (*p* < 0.05). *CobdPBP3* in males exhibited the highest expression of the remaining PBPs investigated in males and females. The expression of *CobdPBP2* did not differ between males and females obviously (Fig. 5).

#### Chemosensory proteins

A total of 14 unigenes encoding putative CSPs were identified (Table 1). The FPKM of *CobdCSP* showed that *CobdCSP14, CobdCSP3, CobdCSP4, CobdCSP10, CobdCSP1, CobdCSP6* and *CobdCSP5* exhibited the highest expression in antenna; the top three FPKM of *CobdCSP14, CobdCSP3* and *CobdCSP4* were 379.12, 370.89 and 92.22, respectively. However, the FPKM of the other seven *CobdCSP* ranged from 1.43 to 12.72 (File S4). Based on the neighbor-joining tree of *CSPs* (Files S2 and S5), the Dipteran (*D. melanogaster*) clade was labeled with green circles; however the CSPs of Coleoptera (*T. castaneum*) were divided into five clades.

#### Sensory neuron membrane proteins

We identified three SNMPs unigenes. The FPKM of *CobdSNMPs* showed that *CobdSNMP3* was much higher expressed than *CobdSNMP1* and *CobdSNMP2* (File S4). In the phylogenetic tree of SNMPs (Files S2 and S6), three clades of SNMPs (SNMP1, SNMP2 and SNMP3) were revealed. The clades of SNMP1, SNMP2 and SNMP3 was labeled with blue, green and black circle.

#### Odorant degrading enzymes

**Figure 6:**
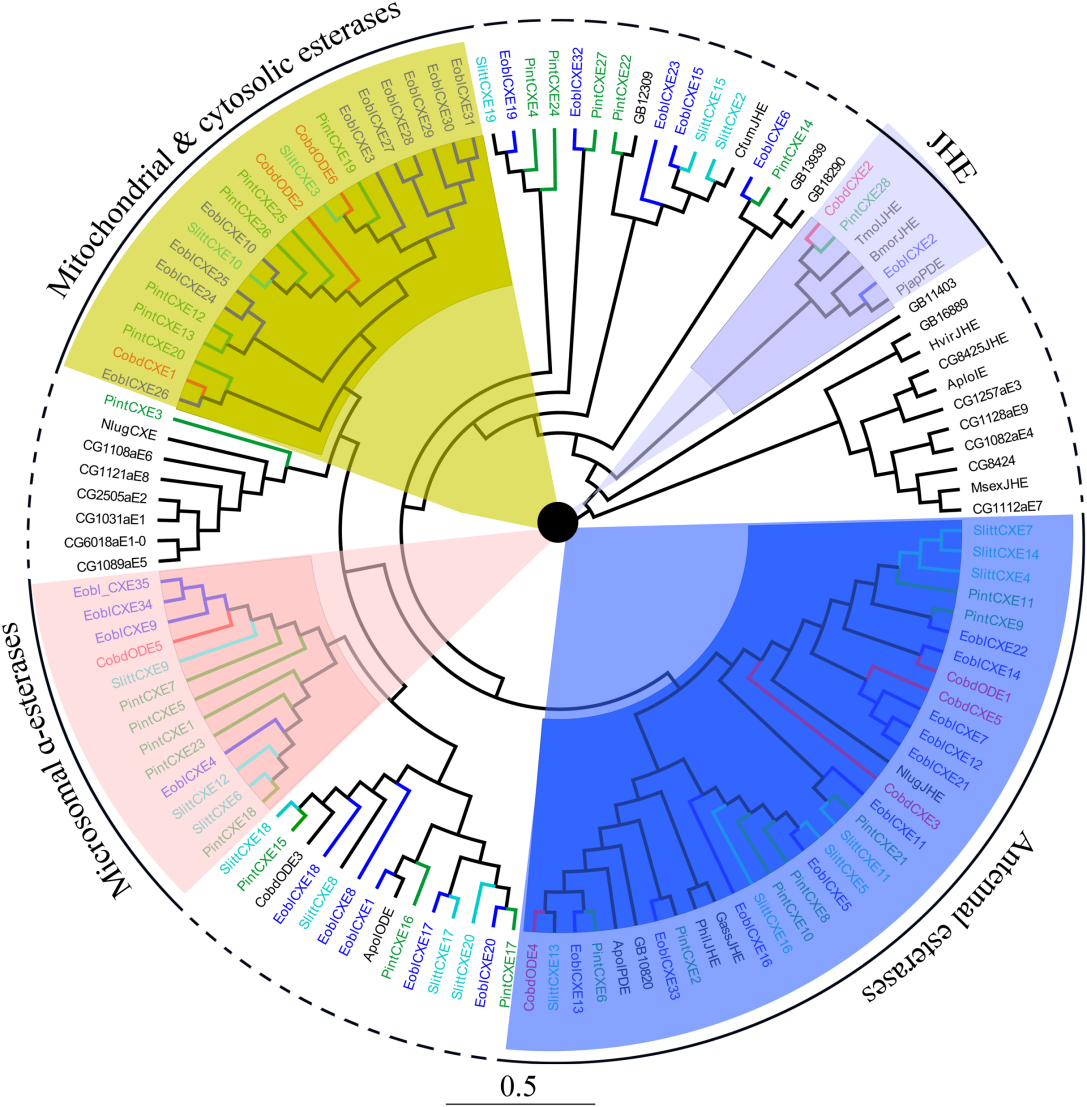
Neighbor-joining phylogenetic tree of carboxylesterases (CXEs). The NJ phylogenetic analysis of CXEs of *C. obducta* (*CobdODEs and CobdCXEs*, red) was performed with reference CXEs of *Plodia interpunctella* (Liu et al., 2019), *Ectropis oblique* (Sun et al., 2017b), *Spodoptera littoralis* (Durand et al., 2010) and Chertemps et al. (2015). The pink, blue, gray and yellow fill area refer to microsomal α-esterases, antennal esterases, juvenile hormone esterases (JHE) and mitochondrial and cytosolic esterases, respectively. The scale bar represents 0.5 substitutions per site.

Six putative odorant degrading enzymes (ODEs) and five putative antennal esterases (CXE) were identified. Nine of these, excluding *CobdCXE4* and *CobdCXE5*, the other nine hadORFs of approximately 1,600 bp. The FPKM of *CobdCXEs* showed that *CobdCXE2, CobdODE3* and *CobdODE4* were the highest expression in the transcriptome; the FPKM of the other eight ranged from 2.86 to 9.72 (File S4). Based on the neighbor-joining tree of ODEs and CXEs (Fig. 6; File S2) and the classification system described in Chertemps et al. (2015), we found that *CobdCXE1, CobdODE2* and *CobdODE6* belong to mitochondrial and cytosolic esterases (yellow area in phylogenetic tree), *CobdODE5* belongs to microsomal α-esterases (pink) *CobdODE4, CobdODE1, CobdCXE3* and *CobdCXE5* belong to antennal esterases (blue) and *CobdCXE2* belongs to Lepidopteran juvenile hormone esterases (JHE) (gray).

### Receptor encoding genes

#### Odorant receptors

A total of 13 ORs were identified. Among them, the full length genes of *CobdOR3*, *CobdOrco* and *CobdOR11* encoded more than 405 amino acids and had complete ORFs. Only two ORs of *C. obducta* were best matches with the same species and sequence (i.e., accession number), the best match for *CobdOR8* and *CobdOR10* was *Helicoverpa armigera* AIG51872.1. *CobdOR10* and *CobdOR12* were the highest expression with a FPKM of 24.94 and 19.56, respectively; the other eleven *CobdOR*s had a FPKM ranging from 1.25 to 7.95 (File S4). In the neighbor-joining tree (Files S2 and S7), the Orco lineage (yellow filled area) included all known insect Orco, *CobdOrco*, *MsexOR2* and *DkikOR32*. *CobdPR1* (Cobd*OR1*), *CobdPR2* (*CobdOR3*) and *CobdPR3* (*CobdOR4*) formed a clade with a PR lineage, including *MsexPR*, *HarmPR*, *ObruPR*, *OnubPR*, *CmedPR1*, *CmedPR2* and three *MsexORs*.

#### Ionotropic receptors

**Figure 7:**
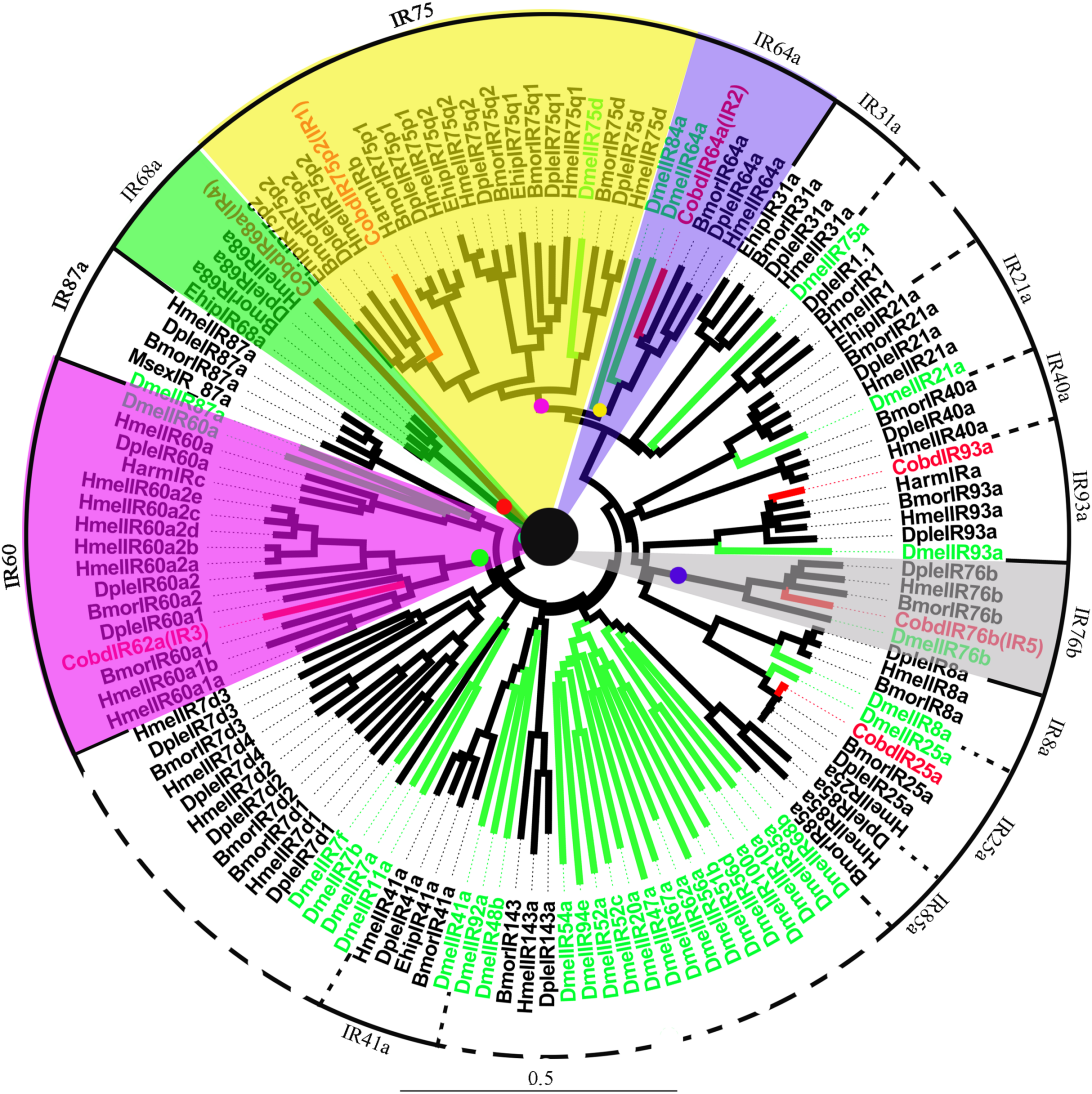
Neighbor-joining phylogenetic tree of ionotropic receptors (IRs). The NJ phylogenetic analysis of IRs of *C. obducta* (*CobdIR*, red) was performed with reference IRs of Lepidoptera, Diptera species. There are 14 subgroup of IRs in the tree. The scale bar represents 0.5 substitutions per site.

A total of 7 IRs were identified in the transcriptome, including IR25a and IR93a. Among them, five were full length genes with complete ORFs longer than 1,500 bp. *CobdIR5, CobdIR4 and CobdIR25a* were the highest expression with a FPKM of 25.69, 23.73 and 12.86, respectively; the other four had a FPKM ranging from 1.6 to 8.22 (File S4). In the NJ tree (Fig. 7; File S2), most IRs were clustered with a known group; the IR62a group contained *CobdIR62a (CobdIR3)*, seven *HmelIR62a*, two *DpleIR62a* and two *BmorIR62a* and the IR25a clade contained *CobdIR25a, DmelIR25a, BmorIR25a, DpleIR25a* and *HmelIR25a*. The IR76b group contained *CobdIR76b (CobdIR5) DmelIR76b, BmorIR76b, DpleIR76b* and *HmelIR76b*, while the IR93a clade contained *CobdIR93a, DmelIR93a, BmorIR93a, DpleIR93a, HmelIR93a* and *HarmIRa*. The IR64a group included *CobdIR64a (CobdIR2), BmorIR64a, DpleIR64a* and *HmelIR64a*,the IR75p2 group included *CobdIR75p2 (CobdIR1), BmorIR75p2, DpleIR75p2* and *EhipIR75p2* and the IR68a group contained *CobdIR68a (CobdIR4), BmorIR68a, DpleIR68a* and *EhipIR68a*.

#### Gustatory receptors

**Figure 8:**
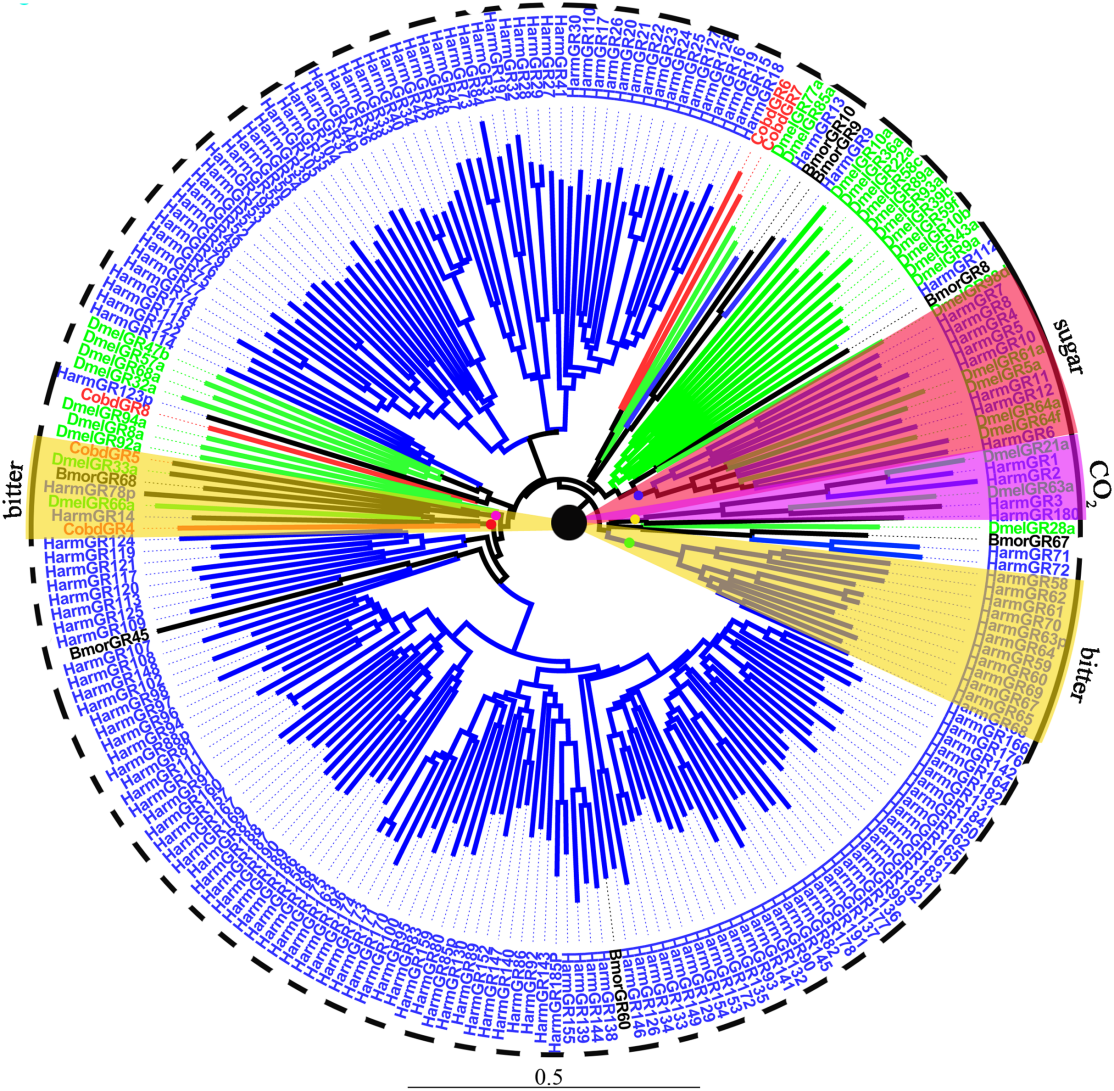
Neighbor-joining phylogenetic tree of gustatory receptors (GRs). The NJ phylogenetic analysis of GRs of *C. obducta* (*CobdGR*, red) was performed with reference GRs of *B.mori* (*BmorGR*, dark), *H.armigera* (*HarmGR*, blue) and *D. melanogaster* (*DmelGR*, Diptera, blue). The GRs group labeled with purple, red and yellow fill area refer to detect CO_2_, sugar and bitter. The scale bar represents 0.5 substitutions per site.

We identified 10 putative GRs, including two GRs for sugar, *CobdGR64* and *CobdGR43a*, which did not have a full-length gene. Four (*CobdGR4, CobdGR5, CobdGR6 and CobdGR8*) were the best matches with *Athetis dissimilis. CobdGR1* and *CobdGR64* were the highest expression in males and females with a FPKM of 172.98 and 70.34, respectively; the other eight GRs had a FPKM ranging from 1.40 to 9.92 (File S4). In the phylogenetic tree (Fig. 8; File S2), the bitter lineage consisted of two subclades, one including *CobdGR4, HarmGR14, HarmGR78p* and *DmelGR66a* and the other containing *CobdGR5, BmorGR68* and *DmelGR33a*.

## Discussion

Olfactory proteins of 246 species of insects have been reported in the NCBI protein database, which includes 51 lepidopteran species (6th-August-2019). However, the olfactory proteins in lepidopteran species that are small and slender with lance-like wings and have larvae covered with a sheath, such as the casebearer moth Coleophoridae, have not been studied to date. We explored CSPs in the body transcriptome of *C. obducta*, based on the feasibility and necessity of exploring the chemical ecology of *C. obducta*. These data provide a direct molecular foundation for understanding olfactory protein function in chemosensory reception. They also show the important function of olfactory proteins in the casebearer moth and establish the groundwork for understanding the molecular mechanisms of olfactory recognition and applying these data to *C. obducta* integrated pest management.

In terms of the number of olfactory proteins, *C. obducta* had similar amount OBPs compared with the whole body transcriptome of *Oedaleus infernalis* (Zhang et al., 2018). Compared with the insect antenna transcriptome (Chang et al., 2017; Nie et al., 2017; Rojas et al., 2018; Sheng et al., 2017; Wang, Liu & Wang, 2017; Wu et al., 2017; Yang et al., 2017, 2018) (File S8), *C. obducta* had an intermediate number of OBPs. The number of olfactory proteins identified in the whole body transcriptome of *C. obducta* is smaller than that inmost other species based on analyses of insect antennal genes. This is probably due to the inclusion of multiple tissues (besides antenna) which would be expected to show a lower abundance of antennal proteins and the exclusion of genes with very low expression levels from our bioinformatics analysis. *C. obducta* is a specialist insect and polyphagous insects with huge expansions of genes associated with chemosensation compared with specialist insects have been identified (Gouin et al., 2017). Importantly, 74 CSPs were identified in the whole body transcriptome of *C. obducta*, including 3 PBPs, 1 Orco, 3 PRs, 3 SNMPs and 2 bitter and sugar GRs, which included most of the important olfactory genes. Thus, the preparation of a whole body transcriptome was a feasible way to search for most of the olfactory proteins in this tiny insect species, which cannot be reared artificially and for which sample collection is difficult.

OBPs are thought of as the first gate in the odorant recognition process with important biological functions, to bind and convey odors across the lymph in the sensillum (Scaloni et al., 1999). The ability of OBP native sensing units to detect odorants and eliminate important behaviors may be useful in the development of novel strategies for insect population management, as well as other biotechnological applications (Brito, Moreira & Melo, 2016). It was recently demonstrated that OBPs can function as molecular recognition units in gas-phase biosensors (Barbosa, Oliveira & Roque, 2018) including OBP22 of *Aedes aegypti* (Zhao et al., 2012). We identified 16 putative OBPs including 2 GOBPs and 3 PBPs. However, from the results of the OBPs phylogenetic tree, determining whether *CobdPBP3* and *CobdGOBP1* belong to PBPs GOBPs, respectively, will require further study.The FPKM values of six *CobdOBPs* were higher than the FPKM values of the *CobdPBPs* in the whole body transcriptome. Considering that OBPs in insects have numerous non-olfactory functions, such as pheromone delivery, solubilization of nutrients, development and insecticide resistance (Pelosi et al., 2018), the strong expression of *CobdOBP4* s, *CobdGOBP1*, *CobdGOBP2*, *CobdOBP11, CobdOBP1* and *CobdOBP8* should make them good targets to determine their expression profiles and functions. Meanwhile, the eight OBPs found in *Ceracris kiangsu* revealed a clear divergence, indicating their varying functions (Li, Jiang & Dong, 2018). It has also been demonstrated that *Cves*OBP2 can bind the *Carpomya vesuviana* male-emitted odor (Z)-3-hexen-1-ol acetate (Li et al., 2017). Notably, most OBPs are expressed in antennae, which indicating the important functions of OBPs in antennal identification processes, such as in *C. kiangsu* (Li, Jiang & Dong, 2018), *H. assulta* (Jin et al., 2015) and *C. suppressalis* (Xia et al., 2015). *AlinOBP11* is expressed in the tarsal gustatory sensilla of *Adelphocoris lineolatus* (Sun et al., 2017a). Expression profiling showed that three *CobdPBPs* were more highly expressed in males than females and two exhibited significantly higher expression in males, indicating the sex biased expression of *CobdPBPs*, similar to *Conopomorpha sinensis* (Li et al., 2018); thus, these proteins may function in male binding of female-emitted pheromones. *CobdPBP3* exhibited the highest expression in males. The same expression profile was identified in of *E. hippophaecolus* for PBP1 (Hu et al., 2018), which is the main protein involved in binding sex-pheromone components during pheromone communication.

We identified 14 CSPs. The top two *Cobd*CSPs, *CobdCSP14* and *CobdCSP3* had FPKM values that were nearly double those of the top two *CobdOBP*s (379.12 and 370.89). OBPs and CSPs exhibit different expression patterns, OBPs are expressed in the antenna, while CSPs do not have a distinct expression preference (Zhang et al., 2013). *Anoplophora glabripennis* CSPs are not expressed in the antenna, but are highly expressed in the maxillary palps and propodeum (Hu et al., 2016b). The expression pattern of CSPs in *Empoasca onukii* showed that CSPs were highly expressed in the head and thorax (Bian et al., 2018). An analysis of the expression patterns of CSPs, indicated that exploration of the functions of *CobdCSP14* and *CobdCSP3* in antenna and other tissues will be important. Some CSPs were significantly expressed in antennae, including most of the CSPs of *Lobesia botrana* (Rojas et al., 2018) and MmedCSP2 and MmedCSP3 of *Microplitis mediator* (Peng et al., 2017). MmedCSP3 can bind insect odors and plant volatiles, as well as pheromone components of *Noctuidae*, Z11-16:Ald, Z11-16:OH and E11-14:Ac (Peng et al., 2017), illustrating the binding ability and functions of CSPs in olfactory recognition. CSPs of Diptera constitute an order-specific clade in the CSP phylogenetic tree, which was the same as *Mamestra brassicae* (Jacquinjoly et al., 2001).

Three SNMPs were identified from the transcriptome. Both *CobdSNMP3* and *CobdSNMP2* were identified as nearly full-length genes and the annotation results suggested they were homologous to *Ostrinia nubilalis* sequences. Moreover, SNMPs are conserved throughout holometabolous insects (Jiang et al., 2016; Vogt et al., 2009); three lineages of SNMPs (SNMP1, SNMP2 and SNMP3) were obvious in the phylogenetic tree.

SixODEs and fiveCXEs were also identified. The biggest groups identified in *C. obducta*, the mitochondrial and cytosolic esterases of *CobdCXE1, CobdODE2* and *CobdODE6* and the antennal esterases of *CobdODE4, CobdODE1, CobdCXE3* and *CobdCXE5*, have also been found in *P. interpunctella* (Liu et al., 2019). *CobdODE5* is a microsomal α-esterases. Such enzymes are well known for their involvement in the detoxification of insecticides and xenobiotics and in the digestion of dietary esters (Campbell et al., 2003; Gong et al., 2017; Yang et al., 2016). The best characterized ODE in *D. melanogaster* is esterase 6, which degrades the major volatile, aggregation pheromone cis-vaccenyl acetate (Chertemps et al., 2012; Mane, Tompkins & Richmond, 1983) and various short chain fatty acid food esters (Benton, 2007; Chertemps et al., 2015).

ORs combine olfactory sensory neurons with binding proteins and they function in olfactory signal transduction, which also uses native sensing units to detect odors. In addition, the interactions between odorant molecules and ORs or OBPs are a source of inspiration for the design of peptides with tunable odorant selectivity (Barbosa, Oliveira & Roque, 2018; Venthur & Zhou, 2018). Insect olfactory receptors are dimers consisting of constant and variable regions (Horsfield, Haase & Turin, 2017). The constant region is a seven transmembrane helix spanning the membrane receptor Orco (formerly OR83b) (Larsson et al., 2004). *CobdOrco* in the Orco lineage of the OR tree demonstrated that we identified *C. obducta* Orco again. The expression of ORs show that 59 ORs of *Tessaratoma papillosa* were primarily expressed in the antennae (Wu et al., 2017) and that most ORs and two PRs of *Loxostege sticticalis* showed antenna-biased expression (Wei et al., 2017), suggesting their putative role in olfaction. It is also obvious that OR subtypes are expressed in different numbers of cells (Fleischer et al., 2018) and can be co-expressed, such as the six co-expressed ORs in the *A. gambia* genome (Karner et al., 2015). Insect ORs appear to be more specifically tuned to odorants than OBPs (Fleischer et al., 2018). In *B. mori*, the receptors for bombykol (the major component of the sex pheromone) and bombykal (the minor component of the sex pheromone) are *BmorOR1* and *BmorOR3*, respectively (Große-Wilde, Svatoš & Krieger, 2006; Nakagawa et al., 2005). *HvirOR13* and *HvirOR6* have also been identified as PRs for the major and minor sex pheromone constituents, respectively (Große-Wilde et al., 2007). Besides, *CobdPR1 (CobdOR1), CobdPR2 (CobdOR3)* and *CobdPR3 (CobdOR4*) are putative PRs in *C. obducta*; the functions of these receptors require further exploration. Identifying ORs, their ligands and key amino acid positions in the receptors (e.g., Ala195 in *AgamOR15* which functions as part of an inhibitor interaction site) (Rahman & Luetje, 2017), could serve as a foundation for the design of pest control agents for a given insect species.

In the antennae of *D. melanogaste*r, ionotropic glutamate receptors responsive to chemical compounds were identified and annotated as IRs (Abuin et al., 2011). The IR group was added and improved based on the genomic analysis of *Heliconius* IRs (Schooten et al., 2016). Overall, the amino acid sequence identities of *Drosophila* IRs range from 10% to 70%, suggesting functional diversity (Richard et al., 2009). Seven IR groups were identified in *C. obducta*, *CobdIR62a, CobdIR76b, CobdIR64a, CobdIR75p2, EhipIR68a, CobdIR25a* and *CobdIR93a*, with different functions. There are three subtypes of IRs, antennal IRs, divergent IRs and IR25a and IR8a, which are expressed, along with antennal IRs, in antennae, gustatory organs (e.g., the labellum) and coeloconic olfactory sensory neurons of the antenna respectively. IR25a and IR8a are co-expressed (Croset et al., 2010; Abuin et al., 2011; Rytz, Croset & Benton, 2013), but we did not identify *CobdIR8a*. IRs also have multiple functions and expression patterns that are general essential chemosensory cues for insects.

Ten GRs were detected in the transcriptome. GRs typically function in sensing sugar, CO_2_ and bitter molecules (Liman, Zhang & Montell, 2014), they were also clustered in groups according to different functions in the phylogenetic tree. The type 2 bitter GRs of *H. armigera* were clustered together in the phylogenetic tree (Xu et al., 2016); two groups of bitter lineages were also identified. *CobdGR4* and *CobdGR5* were clustered with *DmelGR66a* and *DmelGR33a*, respectively, indicating *CobdGR4* and *CobdGR5* function as bitter sensors. *DmelGR21a* and *DmelGR63a* are required for responsiveness to CO_2_ (Jones et al., 2007; Kwon et al., 2007). The sugar receptor lineages included *BmorGR8* and *BmorGR9* (Koji, Kana & Kazushige, 2011; Zhang et al., 2011) and *HarmGR8, HarmGR7, HarmGR4, HarmGR12, HarmGR10, HarmGR6* and *HarmGR5* (Xu et al., 2016), consistent with their function. We did not find that *CobdGRs* function in sugar and CO_2_ sensing. Some GRs are important in pheromone detection, which is required for sexual behavior (Joseph & Carlson, 2015). For example, a previous study speculated that Gr39a participates in female pheromone detection and the authors demonstrated that the knockdown of Gr39a led to less courtship behavior in males (Watanabe et al., 2011). Thus, GRs-binding pheromones are usually considered olfactory signals (Bray & Amrein, 2003; Jeong et al., 2013; Miyamoto & Amrein, 2008; Moon et al., 2009).

## Conclusions

We reported the whole body transcriptome of *C. obducta*; this is the first analysis of olfactory proteins in a Coleophoridae species. We identified 74 olfactory proteins, which will provide a foundation for exploring their functions in olfactory recognition process and system. We also explored the expression profiles of three *CobdPBPs*, which showed that all PBPs exhibited higher expression in males than females, consistent with the previously reported male-biased expression of PBPs. Future studies will explore the functions of the identified olfactory proteins in the antenna of *C. obducta*.

## Supplemental Information

10.7717/peerj.8902/supp-1 Supplemental Information 1 Nucleic acid sequences of all candidate chemosensory proteins identified in *Coleophora obducta* whole body transcriptome.

10.7717/peerj.8902/supp-2 Supplemental Information 2 The protein names and gene accession number were used in phylogenetic trees.

10.7717/peerj.8902/supp-3 Supplemental Information 3 Primers were designed for fluorescence quantitative real-time PCR.

10.7717/peerj.8902/supp-4 Supplemental Information 4 Putative olfactory protein in the whole body transcriptome of *C. obducta*.

10.7717/peerj.8902/supp-5 Supplemental Information 5 Neighbor-joining phylogenetic tree of chemosensory proteins (CSPs). The NJ phylogenetic analysis of CSPs of *C. obducta* (*CobdCSP*, red) was performed with reference CSPs of *D. melanogaster* (*DmelCSP*, Diptera), *Tribolium castaneum* (*TcasCSP*, Coleoptera) and CSPs of Lepidoptera species. Yellow fill area refers to Diptera clade. The scale bar represents 1.0 substitutions per site.

10.7717/peerj.8902/supp-6 Supplemental Information 6 Neighbor-joining phylogenetic tree of sensory neuron membrane protein (SNMPs). The NJ phylogenetic analysis of SNMPs of *C. obducta* (*CobdSNMP*, red) was performed with reference SNMPs of *insect inNCBI database*. The blue, green and black clade refer to SNMP1, SNMP2 and SNMP3 respectively. The scale bar represents 0.1 substitutions per site.

10.7717/peerj.8902/supp-7 Supplemental Information 7 Neighbor-joining phylogenetic tree of odorant receptors (ORs). The NJ phylogenetic analysis of ORs of *C. obducta* (*CobdOR*, red) was performed with reference ORs of Lepidoptera (black) and Coleoptera (celeste) insect. The blue and yellow fill area refer to PR and Orco lineage respectively. The scale bar represents 1.0 substitutions per site.

10.7717/peerj.8902/supp-8 Supplemental Information 8 Identified olfactory proteins and quality index of antennal and whole body transcriptome.

## Additional Information and Declarations

### Field Study Permissions

The following information was supplied relating to field study approvals (i.e., approving body and any reference numbers): The tiny casebearer moth *C. obducta* is a Chinese forestry pest and collection of *C. obducta* is permitted by the leader of Xinganmeng forestry bureau, Yinghua Lu, and member Tianhua Zhen.

### Data Availability

The following information was supplied regarding data availability: The sequence data is available at NCBI SRA: PRJNA587422.
