# Supplementary material for: The whole body transcriptome of Coleophora obducta reveals important olfactory proteins
Source: PeerJ. 2020 Apr 10;8:e8902. doi: 10.7717/peerj.8902 (PMC7153557; doi:10.7717/peerj.8902)
Supplement: Supplemental Information 1 [file peerj-08-8902-s001.doc]

**The whole body transcriptome of *Coleophora obducta* reveals important olfactory proteins**

Dongbai Wang^2^, Jing Tao^3^, Pengfei Lu^3^, Youqing Luo^3^, Ping Hu^1,2^

^1^ Guangxi University, Nanning, Guangxi, China

^2^ Xingan Vocational and Technical College, Xinganmeng, Inner mongolia, China

^3^ Beijing Key Laboratory for Forest Pest Control, Beijing Forestry University, Beijing, China

**Supplementary file 1**

**Nucleic acid sequences of all candidate chemosensory proteins identified in *Coleophora obducta* whole body transcriptome**

>CodbPBP1

ATGTGTGCCTGTGTTCTGTTCCTGCAAGTGGAGACTTCACAGGAGGTCAT

GAAGAAACTGACGATGGGATTCTCGAAAGCTTTGGACCAGTGCAAGAATG

AGCTGAACCTCGGAGAGCACATAATGCAGGACTTCTACAACTTCTGGCGC

GAGGAGTACGCCCTGGTCAACCGGGAGCTGGGCTGCGCCGTCATGTGTAT

GGCCTCCAAGTTCGACCTGATCACGGACGACATGAAACTGCACCATGGGA

AGGCACACGAGTTCGCTAAGAGCCACGGCGCTGATGACAGTATGGCGACA

CAACTGGTCACCATACTCCGCACATGCGAAGAGCAGCAGGCTGCCGTAGT

GGACGACTGCCACAAGACGCTAGAAGTAGCAAAGTGTTTCCGCACTGGCA

TCCACAACCTAAAATGGGCACCCTCCATGGAGGTGATACTCGAGGAGGTC

ATGACGGAGGTCTAA

>CodbPBP2

ATGGTTCACATGAAAGTGTTAGTAAATGTGGTGGTGTTGGGGATGACTCT

ACGGAGAGTAGAGCCTTCAGCAGATACGATGAAACAGATTACGTCAAGTT

TTATGAAAGCTTTAGATGAATGCAGACATGAACTGAACTTGTCGGACCAT

GTGATCCGTGACCTGTACTTCTACTGGAAGGAGGACTACCGCCTGCTGAA

CCGTGAGACCGGCTGCGCTATAGTCTGCATGAGCCACAAACTCAACCTTC

TTGATACCAGTGGGAAGTTGCACCATGGAAACGCTAAGGAGTTTGCGCTA

AATCATGGCGCTGGTGACGACACAGCCTCCAAGCTGGTGGCGGCCATCCA

CGAATGCGAGAAGACGCACGCGGAGGAAGAAGACGCTTGCATGAAGGCAC

TGGAGGTGGCAAAGTGCTTCAGAACTGCCATCGACCAGCTTAACTGGACC

CCAAAGATGGACGTCATCATTACTGAAGTGCTCACTGAGATTTAA

>CodbPBP3

ATGTACATTGTTGTGTCTTGGTCTCTCAATCTGATTCAAATTGTCATCAA

AACTGTTAAGGTTTCGACGGCCTACGGGTTTTGTCATATCATAGTAAATG

TCACTCAACTTGGGAACCCAGTGAAAGTAGGAGATACCTATCCTGAAACA

CTTGGCTATCTCCAATGTGGTGATGCAAGTATTCCTCAAAAGCTTCTTCT

TTATTACTTGTGTACAATCGAATGCCAGCTCTTTAAATCTGTATATCATA

AGTTCGTCCCCTCCATGGCTCCTGATGAAGTGCTTAATATTTGCCTCTTG

AGGCCACCCTTTCTTTGTCACCAGTCCCATCTTCCAGCAAACACAGGCGA

CTGCGCACCCAAACACTTTGGTCAGCGGGTGCTGCGTGTCCCAATAAAAG

TAGAACTCTGTTATAAGTCCGTTCGTTATCGGGACCACTTTTTCACATGC

ATACAGTGCCTGTCCAAAAGTTGCACTCATCTTTCTTATTACGTTTTTGT

AGTACTGATGGGATGCCGTCGATATCCCTCTCACTATTATTAA

>CodbGOBP1

ATGATGAATTCTGCCATAAGCATCTCCATGGTGGGCGCGATGCCGCGCTG

CTGGCACGTGCCCTTGAAGCACTCCGCCACGCGCAGGATGCGCCAGCAGT

GGTCCTGCTCGCCGTCGAACTCCTGTTCGCATTCGTGGATGATGCTTACC

ATCTGCTTCGACAGCACCTCGCCGTTGGGGAAGCTCTTGATGAACTGGTC

CATGTTGTCGTGGTGCATGCGGTGCGAGTCGGTGAGCAGTTGGAAGTGGT

GGCTCATGCACTGGATGGCGCAGCCGAGCTCGCGGTGCTCGAACTTGAAG

TCGTCGCGCCAGAAGTGGAAGAACTCCTCCATCTTCTCCTCGCTCAGCTG

GCTCTCCTCGCGGCAGTGTTTCAGCGCCTCCCCGAAGCCGAGCGTGACGT

CCTTCATTACGTCCGCGCTGGCGCGCGCGCCGCCGGCCGCCAGCAGCAGC

GCCACCAGCGCCACCAGCGCCGCCTTCACGATCATCATTACAAACGATGT

T

>CodbOBP1

ATGGTTAATCAACGTGAACTTTTCTTATTAAAACTAAAAATCTCTCCAAC

ATATCATTACTTCTACAGTTTACAAATCAATTTTAGTCCTGTACACCTTA

CCGTTTTTAGTGCGACCACGTTTCTCGAGTTTCAGGTTTCTCGTTATAAC

TGTGTCTTTGTCGATTTTAAAGTCGTGTACGGTGTCGACGCCCATCATAC

GATTCTTCTCGAGTCCATC

>CodbOBP2

TTGTATCCACAGATCTGGCGCTTGTCTCATTGTTGTGATTGCTGCCTAGC

GCTGGCCGGGGCACTGAGTTCACGTACTTCTCCCCCATACTGCCGGCGCC

GTACCCTCCGTAATCATAGTCAGTGGCATTCAGCACATGGATCTGATCCC

TCGTCTGACTGGTGTTTCGCCGCTCATGGTTGTATTGGTTCATGTGTTGC

CTGTTGTTGCCGTCTATGCGAGGAGTTCGATCCATGTAAGGATCTTCGTT

GCTATATTCATCAGATCTCCGCTGGCGGTTGCCATCGCCCGTACAGGATT

TGA

>CodbOBP3

ATGAAGATTGTTCTGTTCTTGATGTGTGTAGTGGTCGCAGCTGCAGCCCA

TAACGTGCAGCTATCGCAACAGCAAAAGGAAAAAGTCCGCAAATACACAG

CAGAATGTATGCAGGAAACTGGCGTAAAACCTGAGCTTCTAGCCCAAGCC

AAGAAAGGCGAATTCACTGATGACGAAGCTCTGAAGAAGTTCACTCTTTG

CTTCTTCAAAAATTCAGGACTTTTAACTAAAGATGGAACGCTGAATACTG

AAGCAGCTCTTTCGAAATTGCCTGCTGGAGTTGACAAGGCAGCTGTAGCT

AAAGTGCTAGATGAATGCAAGAAAAAAAAGGGAAAAGATTCTGCTGATAC

AGCTTTTGAGGTGTTCAAATGCTACCACCGCGCTACTCCTGCCCATGTTG

TGTTCTAA

>CodbOBP4

ATGGGTTCAAACGGTGTTATGTACTTGGCTTTTGTTTTAATTGTGCCTTT

TCTTGGCGCTGCGATCGCCGTTGAAAGAAAAGAATGCCACATGAAATTGG

GACATCCTGCCAACTTCCACTGCTGCAAGGATATCCAAAATAAACGTGAA

AATAATAACGTCCGTGATACGTTCAAAGAGTGCTTCCAGCCACCCAACAA

GCCACCTCCATCTTGTGACACTGATATATGCATGGCTAAGAAACGTGGGT

ATCTAGCAGATGACGGTTCAATAGACAAGGCAGCCCTAGAAAGCGTTGTT

AAGAAGGACTCCATCGACGATCCAGTCTTGGCTGAGGCTGTAATAACCAA

ATGTGTTAATGGAGATATCTCTGACTATGGCCCACCA

>CodbOBP5

ATGGCAAAACTGACTCTATTATTATTCACACTGGGTTTGTTGACTGTTGC

TTTGACATCGACAATACCTGGTGAACCTAAAAAGAATAAAGGTACAACAC

CCCCAGGAGTTGTAATGGAGACTTTAACTAATAATACCATGGATGATGAC

CAGGAGCCCATGAATGATAAGGATTCGAAGGATGTAAACTTGATGGAAAT

AATGAACGAGTGCAATGAGACGTTTGTAATTCAACCATCGTACCTACAAG

CCCTGGACGAGAGCGGCAGCTTTCCCGACGAGACAGACAGAAATCCAAAG

TGCTACATTCGCTGTGTTTTGGAAAAGACCGGGGTAGTGTCAGAAGAGAA

GTTTGACCCAGCGCGGACTGCCGTAGTGTTTGCAGGCGAGAGAGGGGGCA

GGCCCATCAATGATTTGGAAGCTATGGCAACTGCTTGCGGGTATAATAGA

GTGGAACCATGCAAATGCGAGAGGTCATACCAATTCATGAAATGTTTAAT

GGAGATGGAGATACAGCGCTATGAAAAATCTGCAAAATAA

>CodbOBP6

ATGTGTCTTGTTAAAGTTTGCGTTTTTATATTTCTAGTTTTGTTTTTAGA

ATGCTATAATGCTTTAAACTGTCATTCTGAAGGTGGTCCTAAAATTGAAG

AGCTGAAGTCAATCTACATGACTTGTCTGAAAAAGCAAGATGGCAAAAAC

AACAATCGAGGACATTCAGGAGATGAAAATTGGAATTCTAGGGGATCCAA

TCAAAAATGGGATAGAGAGAGGAGATTGGGAAGTAGAGATGATAGGATGT

CTGGGAGAGATGATAGGATGAATGACAGAGATTACAGAACAAGTGGGAGT

GGAACAGACGATAGGATTAACTCAAGGAATGACAGGGATAGAATGAATGA

TGGTTACAATAGAGATGATAGAATGGGAAGTAGAAATGATAAGGTGAGTA

ACAGAGACATGATGAGTCGTAATGAAGGATACGGAAGAACCGATATAACA

GGGAGAGATGACTTCCCACTCAATAATGATTTTGGAAGCGAATCATCATA

CAATACGTACAGACCCACAACGCAGACTCCTCGGCGATTCAGGCGTGACA

AACGCACAGGCATGAACTCGGGA

>CodbOBP7

ATGTTTAAGCTTGGATTCATGTTTGCTTTGTTTGCTATCTTAGTAAGGAA

TATAACGGGGTTAAATGATGAACAGAAAGGACAGATACAATTCCAGTTCA

TCGAGGCAGGTGCTGAGTGCATCAAGGAACATCCACTGTCTGAAGAGGAT

ATCAACTCCTTCAAGGAAAATAAGTTTCCCAGCGGTGAAAATTCGGGATG

CTTCGCTGCGTGCGTGTACAGAAAATTAGGTTTTATGGATGACAAAGGAA

CTCTTACGCATACCGATGCTTTAGAGAATGCAAAGAAAATCTTCGAGGAT

GAAGAAGAACTGAAGAACATTGAAGAATTCCTTTCAACTTGCACTAAAGT

TAACAATGAGGATGTCAGCGACGGAGCAAAAGGCTGCGAACGCGCTGCCC

TAGCCTACACTTGTCTCACCGAGAACTCTGAGAAGTTCGGGTACAACATT

GGCTTCTAA

>CodbOBP8

ATGTCAGTTTTGAAAATTATATTTGTATTTATAGCGCTTTTCAGTTTATG

TTCTTCCTATGTTAATATAGAGAGATACATGAAGATATGCCATCGGAACT

CGCCGGAGGTGAACGAGTGCTTAGTGGACGCGATTCAGTCCGGGTTGGTG

ACGCTGGCTCCTGGCATACCGGAGCTGGGAGTGCCCCCCACAGACCCCTA

CTATCAGAAGGAATTGCGCATCGACTATAAAAATAACCAGATAATAGTAA

AAATGGTGATGAAAAACGTAAAAGTTATGGGTCTGAAAGATTCTACAGTC

CACAAAGCAAGGCTGCGGGCCGACGAGGATCGATTCTACCTTGAAGTGGA

CATGACCTCTCCGCGCGTGTCGGTCACAGGCGAGTACGTCGCAGAAGGAT

CCTACAACTCCCTGCAAGTTAAAGCCTTCGGGAAGTTAAACGTCAATATG

ACGAATCCCGTTTACACGTGGAAAGTCGAAGGGACCCCAGAGAAACGTGA

CAATGAGACGTACATAAAGATCCATAAGTTCTACATGCGTCCCGACCTAT

CGAACCTTAAGGCCTACCTGACGAACGATAACCCTGACACGGCAGAACTC

GTTAGACTAGGCAACGACTTCGCGAACAGCAACTGGAAGAGTCTTTACCG

CGAGTTGCTGCCCTACGCGCAAGAAAATTGGAACAGAATAGGCATTCGTA

TTGCGAACAAACTGTTCCAGAAAGTGCCTTACGACCAGTTTTTTCCTACT

GATGTATAA

>CodbOBP9

ATGTGTCGTAAATTATTTGTGGTGTTTGTTGTAACATTAATTACTACACA

ATTGTCAGGAGCTCCGAATGTCACAAAATGCAAGGCTTCTGATTCGAAAT

GCATAAGAGCATCAGCAGTGACTTTGTTCCCAGCATTTGCAGCAGGTATT

CCTGAGATGAACGTTGCTTCACTCGATCCTCTGCATATGGACATAATGGA

TGCTAGCACTCCAGGCCTAAAACTGGTGTTGCGGGATTCAGCCGTAACTG

GATTGAGGAAATGTGATTTAAAGACAGTGGAACGTAACGTGGCCAAGTCT

AAGCTTCTCGTGACAGTGCTCTGCGATGTGGACGTGAAGGGCAAATACGA

GTTAGATGGTCATATCTTGATACTGCCCATGCAAGGAGAAGGTGATGGCC

ACGTGCAGCTGAATAAACTCCTAATAGATATCGAAGCAAACTTAGGTGAG

AAGACTGACAAACAAGGTGTTAAACACTGGGACGTCAAGAGCTTCACGCA

CAGTTTTGACCTGCAGGACAATGCCAAGATACATTTGGATAATCTCTTCC

ACGGAAACAAGGAATTAGCTTCGGCCGCGAACACTCTCTTCGATACCAGC

GCTAATGAAGTGATTAAAGAAGTCGGCGTGCCCATTGTGAAAAGTATCGT

TACGGAAGTCGTGAAGAACGTGAATCACTTCTTCCATAGAATACCAGCTG

AGGACCTCTCCCTAGATTAA

>CodbGOBP2

ATGAAGGCCGGTGTTAAGGTATGGTGGCTGGTGGTCCTGGGGGTCCTTGC

TTCAGTGAAGGGAGATGCTCAGGTCATGAGCCACGTGGCTGCGCACTTTG

GGAAGACTCTGGAGGAGTGCCGCGAAGAGTCAGGGCTCCCAGTGGAGGTG

ATGGACGACTTCAAGCACTTCTGGCGTGAGGACTTCGAGGTGGTGCACCG

CGAGTTGGGCTGCGCCATCATATGCATGTCCCAAAAGTTCTCCCTGCTCA

AGGACGACACCCGCGTTCACCATGAGAACCTCGATGATTATATTAAGAGC

TTCCCTAATGGTGAAATCTTATCGAAGCGCTTGGTGGACATGATCCACAA

TTGTGAGAAGCAGTACGATGACATCGATGATGAGTGCATGCGCGTGGTGA

AGACGGCGGCGTGCTTCAAAGTGGACGCGCGCGCAGCCGGAATCGCGCCT

GAACTCGCTATGATCGAGGCGGTTGTGGAGCAATACTGA

>CodbOBP10

ATGGGTCTCCTAGTGGTTGTTGCTAAATTCCTATTGATTTTCACATTTTG

CAATGCGATGACCATGAAACAAATCAGAAACACTGGGAAAATGATGAGGA

AATCATGCCAGCCAAAGAACAACGTAGAAGATGAGAAAATCGATAAAATT

GGTGACGGCGTATTCATAGAGGAAAAGGAAGTGATGTGCTACATGGCATG

CATCATGAGAATGGCAAACGCGGTGAAGAACAATAAGTTTAACTACGAGG

CAGCTATAAAGCAGGCTGATCTGTTGTTACCTGATGAAATCAAGGAACCA

GCGAAAGAAGCACTCACGGTCTGCAGAAAAGTTCCGGAAAGCCACAAGGA

CATCTGCGCAGCAGCATTCCACATGACAAAGTGTATTTATAACCAGAACC

CCGAAGTTTTCTTCTTCCCTTGA

>CodbOBP11

ATGGGAAAAACAACAAACAACAAAATGTTTGAATTATTCATCGTTATAGC

AATATTTTCCGTAACTAATTGTGATTTATTAGTCGAGAGACCAAAAGGAG

CCAGTTTGAAACCACTATCAGCGTGTTGCGATATTCCAGAGCTAGGCGAT

GCTAAACCTCTAACAGAGTGTTCGATCCCAAAGATACCTGGCCCTTGCAA

TGACATCCAATGCGTCTTCGAGAAGTCCGGTTTCCTGGTCGACAAAACCA

CCCTGAACAAGGAGTCTTACAGAAACCACCTTCGCACGTGGTCTGGACAG

CATCCCGGCTGGTCGCAAGCGGTGGAGAAAGCCATACAAGACTGTGTTGA

TAAGGACCTTCGGCAATACCTGGACTATCCCTGCCGGGCTTACGACGTAT

TCTCATGCACTGGCATTGCTATGCTGAAGAAATGTCCGAAGGAAGCATGG

AAATGTTAG

>CodbCSP1

ATGCAGCTCCACCATATTGTAGTGGTGTGCGCGGTGGTCGCGGTGGCGGC

CTGCGCGGCGCCTGCGCAGACGCAGCGCCCGCAGGTCTCCGACACAGTGC

TCGACGACGCGCTCAGTGACAAGCGGTTCATTCAGAGACAGCTCAAGTGC

GCGCTGGGGGAGGCGCCGTGCGACCCCATCGGGAAGAGATTGAAGACCCT

AGCCCCCTTCGTGCTGCGCGGGGCGTGTCCCCAGTGCACCCCACAGGAGA

CCAAGCAGATCCAGCGCACGCTGGCCTACGTGCAGAGGAACTACCCGCAG

CAGTGGGCCAAAATCGTGCGCCAATACTCTGGCTAA

>CodbCSP2

ATGAAGCTAATCCTCATAGCGGTTGCGATGTCCATATTCGCCCTGATCGG

GGCTGAGGAGGACAGATACACGGACAAGTATGACAACATGGACATAGAGG

AGGTGCTTTCTAATAAGAGGCTGCTCAACGCGTACACCAAGTGCGTGCTG

GACCAGGGTAAATGTACTCCTGAGGCTAAGGAGTTGAAATCCCACATCTC

CGAGGCTCTCCAGAATGGCTGCGCGCAATGCACCAAGACTCAGAAACAGG

GGATGAAGAAGGTAATAAAACACTTCATCAACGAGGAACCTGACTCCTGG

AAGCAGATGGTGGAAAAATACGACCCGCAAGGCCGGTACTCCAGCAAATA

CGAAAAAGAGATGAAAAGTATAATGCACTGA

>CodbCSP3

ATGATGGCGAAATACGTCCTAGTGCTGTGTTGCCTTGCTGCGGCCCTGGT

GGCCGGCCAGAAGTACACGGACAAGTACGACAACATCAACCTAAAGGAGA

TCCTGGAGAACAAGCGCCTGCTGCAGGGATACGTGGACTGCGCGCTGGAC

AAGGGCAAGTGCACCCCGGATGGCAAGGAGCTCAAAGAGCACATCCGAGA

GGCCATGGAAACCGGCTGCGAGAAGTGCACAGAAACGCAGAAGGACGGCA

CTTACACCGTCATCTCGCACCTCATCCACCACGAGCCGGACATGTGGAAC

GCTCTTTGCGCCAAGTACGACTCCGCCGGCCAGTACCGCAAGAAGTACGA

GGACCTGGCCAAGGAGCGCGGCATTGAGCTCCCAAAGTAG

>CodbCSP4

ATGTCCGTCAGATCCTCTTTTAGAACTTTGTCGTCTTTCGTGCAGTTGCT

GTCATCTTTAACGCAGTTCAAAAATCTTCTCGCATTTGTCGTATTTGAGA

ATATCGTTTCAACATTAAAGTCTTCATAG

>CodbCSP5

ATGCGTGCTGTGCTATTTCTTAGTGCGAGTGTGTGTGCTTGGTATTTGGT

GGTGGGACAAGACATTTATGATATGAGGAATATGCCCAAATACGAATCCA

GGTACGATTACCTAGATGTTGACGCTATTCTGGATAGCAAGAGACTTGTC

AGAAATTACGTGCAGTGTCTTATAAATTCGCAAAGGTGTACACCTGAAGG

AAAAGCGTTAAAAAGAATACTCCCCGAAGCGCTTCGCACGAAGTGTGTGC

GTTGCACTGAGAAGCAGAAGAGAACAGCAGTGAAGGTGATCAAGCGCATC

AAATACGAGTATCCAGAAGAGTGGGCAACGCTGTCCGGCAAGTGGGACCC

CACTGGTGACTTCACGCGGTACTTCGAAGAGTTCTTAGCCAAGGATAACT

TCAACGCGATCTACAACAACGGTAACGAGAAACCCAGCTCAACGCTTCCA

CCGCCGCCGCCGCCGCCGTCACCCCCATCGCCCCCATATCTTCCGGCGTC

GCCCCCGCCGACCCCGTCCATCCCGCCGTCGCTGCCGTCGCTGCCGCCCC

TGCCGCTGACGTCACTGTCAGAAGCGCCGCAGCCGCCAATGACGATGACA

ACTGAGACTCGACCACCTGTCATACTCAATAGGTTCGGAGACGATGGCGA

GCTGATGATGCCGAGCCCGTCTTCACCTGCCTTCACCCCTCAAGCCACGA

TGATGCCGCCCTCGTCCATGCGGCCGGTGGTCGCCACCATGAGGCCTCCC

ATGCAGCCTCCTATGCAGACCAGACCACCTCCATTGACATGGACCGGGGC

AGCCTCGGACACGATGCCAACGCGGTTCCCATTACGACCGCTCAACATGC

AGCCTTCTTACACGACGGCTATCACAATTATCGACCAGATCGGTTACAAG

ATCCTGAGGACTACCGAACTTGTCACGGATATACTGAGGAACACGGTTCG

CGCAGTTGTCGGTTGA

>CodbCSP6

ATGAAATACCTGGTCCTGCTGTCACTGGTGGCGCTCGCAGCCTGCGACAC

CTACACTCCGGATAACGATGATCTGGACATAGAGGCTGTGGCGGCAGACC

TGGACAAGCTGAAGGCTTTCTACAACTGCTTCATGGACACTGGATCCTGT

GATGCTGTGCAGCAGGATTTCAAGAAGGACCTTCCCGAGGTGGTAGCCGA

AGCGTGCGCTAAGTGCACCGCGAAGCAGAAGCACATTGCCAAGAAGTTCT

TCGAGAGCCTGAAGCAGAAGCTACCGGAGGAATACCAGAACTTCCAGAAG

AAGTACGATCCCGAGTCTAAGTACTTCCTCAAATTGGAGACAGCTGTTGC

CAACTCATAA

>CodbCSP7

ATGTCTACTCTCCTGCATCTCATAGTTACGCCTTACTTCTTGATTATGGA

GATCATTGTGTCCGTCAACAGAATACACTTCTTGGTGCTGCAGTTCCTGT

GGTGCGCCACGCACGTGCTGCGACTGTTTGTGGTGGTTGAGCCCTGCCAT

TATACCATCGCAGAGGGCAAAAGAACAGAAGGCCTAGTCTGCCGGCTGAT

GATGTCAGCGCCATCTACCGGCGTACTC

>CodbCSP8

ATGACAACTTCAACGCCGACTCCATCATTCAGAACGACAGGGTTCTGCTC

GCTTACTACAAGTGCGTCATGGATAAAGGACCTTGCACCAAGGACGGGAA

GAACTTTAAGACCGTGTTACCCGAGACACTGTCGTCGGCTTGTGGGCGCT

GCTCCCCGCAGCAGAAGGCTGTGGTCCGGAAGCTACTCCTCGGCATCAGG

GCCAAGAGTGAGCCGCGCTTCCTCGAGCTACTGGACAAGTACGACCCTCA

GCGAATCAACAGAGATGCCCTCTACACTTTCCTCGCCACTGGCCAGTGAG

CAGTCTAAGGACACATTAATGGACACTTGA

>CodbCSP9

ATGGAACTGGCACACCTGGCCATGTGGACGATACTGACAGTAGTCGCGGC

AGACTTTTACAGCGCTCGCTACGACACCTTCGACATCCAACCTTTGCTGG

AGAACGACCGGATTCTGCTGAGCTATACGAAGTGCTTCTTGGAGACAGGG

CCTTGTACGTCAGAGGCCAAGGACTTCAGAAGAGTGATTCCCGAAGCTTT

AGAGACAACCTGCGGCAAATGCACGCTAAAACAGAGGGAGTTGATTCGCA

AGGTGATCCGGGCGATCGAGGACCGCCACCCTGAATCCTGGAGCCAGCTG

GTGGACAAATACGATCCTGACAAGAAGTTTCGGCCCGCCTTCAACAATTT

TCTCAAAGAGGAGGATTAG

>CodbCSP10

ATGAATGCCTTAATTGTGTTTGGTCTGGTGGTGGTGGCAACGGTGAGCTG

TGTAGAGGTAGACACCTTGGAAGACATGGATATTGAAGCTCTTCTGCAAA

GTGAGGACCAGAGGAACGCTGCCTTCGCCTGCCTGATGGACCAGGCTCCA

TGCGGGAAGCTGCAGAAACTGCGAGACAGCATCCCCACGATGGTGGAGAC

GAAGTGCGCGCTGTGCACGCCCAGACAGAAGGAGAAGTTCGACCAGGTTA

ACAAGATGCTGTTCGCCAAGTACCCCAAGCAGTTCCAGGAGCTGGCACTC

AAGTACTCGCCCAAGTCTGACTAG

>CodbCSP11

ATGCCAAAGTCTTGCTCCGTGGAATATATCCCGCGGATATTTATGTCCAC

TGGGTCTGGGATTTTCTCGGATTTCTCTGTAGAATTAGCTCTGAACAGCT

CAGCAGTATTCGGTCGGTATGTGTCCAACGAAGCGCTAAGTTCCATGAGA

GGCTTCATAATGGCTTTTAGGTGTTGCAGCTGCTCGCATGCGAACGCAAG

CCAGGAGCAGAACATCACGTCAAGCAGGTTAGCAGTCATCATCGAGAAGA

GGAGCCAGTACACCTGA

>CodbCSP12

ATGCAGGCAACAGTGCTGCTCGCCCTATGCGCGTGCGCGTGCGCGGTGCC

AGCGATCCTGTCGTCCCCAGCGCCACCACCGGCGCCGCTGCAGATGACGG

ACGCGCAGTTGGAGCAGGTCCTGAACGACCGCGCTACCATGCAGCGCCAC

CTCAAGTGCGCTACTGGTGAAGGACCCTGCGACCCTGTGGGCAGGCGACT

CAGAACTCTAGCCCCGCTGGTGCTGCGCGGCGCTTGCCCCCAGTGCTCCG

CGCAAGAAGCCAAGCAGATCAGGCGCACGCTCGCCCACGTGCAGCGCAAC

TACCCCTGGGAATGGGCCAAGATCGTGCGCCAGTATGGCTGA

>CodbCSP13

ATGTTTCTGATGGTGCAAATGTATTTTGGTTGCACGATGTTCAACCTTAT

TCCCTTCTACAGAAGCCTGAACGATGGCGCGTTCCGCAACGATAAGCCCG

CAAACGTAACTTTTAAACATTGTGTATACTTAGAACTACCTCTTGATTAT

GAACACGACTTCAAGTCCTACGTTTACGTGGTTTTCCTGAATGTGTATCT

TACCTGCTTGTGTTCCACGCTTTTTTGTACAGTTGATTTGACGATCGCCT

TGATTGTTTTGCACCTATGGGGTCACTTAAAGATTCTCCTTCATGATTTG

GAACACTTCCCCAAGCCAATCATTGATGGGCCAAGCAGCAAGTTTGCAGA

TCATGAGATGAAAAAAGTCTTCAGTCATCTGATAGACATCATCCAGCATC

ACAAATTCATTTTGGAGTTTACCCAACAATTATCTGATACTTACAGGCCA

TTTTTAATAATATACCTGGCTTTTCATCAAGTAGCTACATGTATCTTGAT

GTTGGAATGTTCGCAAATGACAGTAGATTCGCTCCTGCTGTATGGACCCC

TAACCATTATTTTGCTACAACAATTGATTCAGTTGTCTGTTGTTTTTGAA

TTAATGTACTCTACGAACGAGAAGGTCCAAGATTATGTCTACAAT

>CodbCSP14

ATGAAATCCTTCGTGGCTGTGTGTTGCCTCGCCCTGGCCGCTTTAGCCGC

CGCCCGCCCCGAGGACAAGTACACTGACAAATACGACAATGTGGACGTCA

AAGAGATCGTCTCCAACAAGCGGCTGTTGACGCCGTACATCAAGTGCGTG

CTGGACCAGGGCCGCTGCTCAGCTGAGGCAAAGGAGCTGAAAGCTCACAT

CTCGGAAGCCCTGGAGACTTACTGCGCGAAGTGCACGGAGGTGCAACGCG

AGAAGACCCGCCAGGTGATCGGCCACCTCATCAACAAGGAGCCCGAGTAC

TGGAGCCAGCTCTCTGCCAAGTACGACCCGCAGAGCAAGTACACCGCCAA

ATACGAGGCTGAGCTGAAGACCCTGAACCCTTAG

>CodbODE1

ATGCCTGCACCAACATGGGTTCAACCGCTAGAAGCCTTTGATAGAGGAAT

AATATGTCCACAAGTCATTGTCATGCGTCTTGAAGACAAAAGTATGATCG

ACGACTGTCTTATTGCAAACGTTTATGTCCCGGACACCGCTGGTGCCGGG

ACGCTACCAGTCATCGTTTACGTGCACGGTGGAGCCTATCAAGCAGGCTA

CGGTGATTTGATCACACCAAAAAGAATTGTACGAAGCAAAAAGATAATAG

CCGTCACATTTAATTACAGGCTTGGAATCCAAGGATTTCTTTGCTTAGGA

ACAAAAGACATACCTGGAAATGCGGGAATGAAAGACCAAGTGGCGCTGTT

GCGCTGGGTGAACAAAAACATCGCAAACTTCGGAGGTAATCCTAATGACG

TCACCCTCGCAGGGTACAGTGCCGGTGGCTCCGCCGTCGATTTGTTGATG

CTGTCAAAAATGGCAACTGGTTTGTTCAACAAGGTAATTCCAGAAAGCGG

CTCCAATCTTGCCGTATGGTCCATTCAAATAGATCCCCTGGAAAATGCTA

AAGCATATGCGAGAACACTGAACTTTACAGATGTGGATGAAATCTATTCC

CTTGAACAATTTTATAAGACTGCATCATTGAAGATGTTGACGCAAGAATC

ATTTCTGTACAGACCTGATTCCACTTTCGTATTTGTACCTTGTATTGAAC

AAGATACAGGAACCGAAATGTTTTTAGAGGACTCTCCAGTGAATATTCTA

AAAGCGGGCAATTATAGCAAAATGCCGATGTTATATGGATTTGCCAATAT

GGAAGGTATATTTCGCGTTCAAACGGGGTATGAAAAATTTCGGTATGACA

TGAATAATAAATTCTCAGACTTCCTACCAGCTGATTTGCAGTTTAAAAGT

GAGGAAGAAAAAGATAAGATTGCAAAGCAGGTAAAAGAGTTTTATTTCGA

TGATGAAGCAATAGGTGATCATACTATTACAGGATACATAGACTATTTTA

CTGATGTTATGTTTGCTTATCCGGTGTTAAGAGCCGTCAAACTGCATTTA

GCAGCAGGCCATGACAAAATATTTTTGTACGAATATTCCTTCGTAGATGA

GGAATATTTGGAATCTGATGAAGGTGTCTTGGCTAAAGTGCGAGGCGCTA

ACCACGTGGCACAGAGTACAGCGGTTCTTGACCAGAAAACCATGAAGGGT

AACGAAGAAGAACATCTAACAGAATCTTACCAGAAGATGAAGTCCATTAT

AAGGGAATTATGGCTTAACTTCATAACGAGCGGCGCACCTGTGCCAGCGG

AGTCAAGGCTGCCCGCATGGCCGGCGACAGGGTCACAGGGGGACCCGCAC

ATGTCGCTGAACAGCACTCTGCGCGTGCGCGGGCCGCTGCTGCAGCGCCG

CGCGCGCTTCTGGCACGCGCTCTACGAGCAGCACTACCGCGCGCCGGCGC

CGCCCCCCGTGCACCCGCCGAGACCTCGATATGATCTTTAA

>CodbODE2

ATGGTACAGGTGACAGTAAAAGAAGGAACCCTAGAGGGAGAGTTAGTTGA

CAATGTGTTTGGAGGAAACTACTATAGCTTCAAAGGTATTCCCTACGCCG

AGCCCCCGACTGGAGATCTACGATTTAAGGCTCCAAAACCCGTAGAACCA

TGGAGCGGTGTGCGAAACGCTAAAGAGCACGGTCCAGTCTGTTATCAATA

TGACAATATTATCATGAAAAAAATGCCTGAAGACGGTAGCGAAGACTGCT

TATACCTTAACGTCTACTCTCCTCAAATAAAACTGGGAAAACCTTTACCA

GTCATGGTCTTCATTCACGGAGGAGCATTTGTGAGTGGAAGTGGTAATAG

TGACATGTATGGACCCGAGTTCCTTGTTAGAGAAAACGTAATCCTTGTCA

CGTTTAACTATAGACTAGAAGCACTTGGTTTCCTCTGTTTGGATACTCCA

GAAGTACCTGGTAATGCTGGAATGAAGGATCAAGTAGAAGCTCTACGATG

GGTAAAGAGAAACATCAATAGCTTCGGTGGTGATCCTGAAAATATAACAA

TTTTTGGTGAGAGCGCAGGTGCAGCCAGTGTAACCTTCCATCTAATATCG

CCAATGTCTAAAGGCCTTTTTAAAAGGGCTATAGCTCAAAGCGGAAGTGC

AAATTGCTACTGGGCTTTGGCTGTAGAACCTCGTGAAAGGGCTCTAGCAT

TAGCAAAGAGTTTGGGTTGTAAGTCTGAAGATGACAAAGAGCTGTATGAG

TTTTTGAAAACACAATCTAAAGAGTCGCTTATCAATATAAAAGAGTATAT

TAGCTTAAAAGAAATTGCAAGAGAAAAAAAAGATCTTATTTTTTCAATTG

TAGATGAGAAACCATTTGAAGGTCAAGAAAGGTTCTTCCATGGTGACGTC

TTAGAAGCCATACGTAGCAACCTTCACGAAGGAGTAGAAATAATAGCTGG

GTACTGCAATGATGAGGGCATTTTGGGTTTATCTGGTGAGTTTCAGCTTG

ATAAAGAGGTGACTCAATTAAACAGGTTCCACGAAATGTTCGTTCCAAGA

CCATTATTCGATGACTTAGCCCTTAAAGATATCTTGGAAGCTGGAAGAAA

AGTCAAAAAATTCTACTACAAAGATGAACCCGTCACGATGGAGAATTTAG

GCCCGATGGCAAAATATAATGGATTCACCAGGTTCACTTTTAATATCGTT

AATTTCCTTAAAGAGTGTGCGAAGAAGGGCAGTGATGTCTACTTGTACAA

GTTCATGTGTAGCTCTGAAAGAAACATTTTGGGGCACGTGTTGAAACTGG

GCAAGATAATTGGCAAGACAGAGGCTGTATGTCACGCTGATGACCTTGGC

TACTTGTTTCATATTAAGATGTTGGAATCCAACTTTGAAGAGAACTCCAA

GGAGTATAAAATGATCAGGAATTTTGTGAAGCTTTGGACCAATTTTGCAA

AATATGGGAACCCAACACCTGACAGCAGTCTAGGAGCCAAGTGGTCTCCA

TTTACACTGAAGCAGCAGGAATATTTGGAGATTAAAGAGAGTCTGGTCCC

AAGAGCAAATCCAGAAAAGGAGGAGTTCGACTTTTGGGAGGAAATCTTCA

GGACTTATTATCCGCAGAGACTTGCTTAG

>CodbODE3

ATGAAGTGCGGGAAGAAACTAGTGTTGTTTTCGCTGTTTGCGATGAATTT

AGTTGATCAACCAGCTCCGGAAGTGAGGATTGAACAGGGTATAGTGAGCG

GGATTATCAGCAATGATGGCAAGGTTTTACAGTACATAGGCATACCTTAT

GCCACTGTGAATAGTACCAACAGATTTAAGGCACCATTGCCGCCTGCAAA

ATGGGAAGGTGTTTTCAAAGCAATCACGGAATACCAATGTCCACAAACGA

TGAAATCCTTCGTTTTTGGAACAGAAGACTGTCTCAGAGCAAACATCTAT

GTACCTGTCACAACAGCACCTAAACCCCTGCCTGTTATGATCTACATTCA

CGGTGGAGCTTTCATTTTGGGCAATGGAGGGAAACTTATGTATGGGCCAG

AATTCATAGTCAGACATGACGTAATTCTAGTCAATATAAATTACAGACTT

GGAATACTTGGATTCTTATGTTTGCACACAAAAGAAGCTCCAGGAAATGC

TGGTATTAAAGACCAAATTGCTGCGTTGAGGTGGATAAAGAAAAATATTG

CTGCCTTTGGTGGTGATCCAGATAATATAACTGTTTTTGGTGAGAGTGCT

GGAGGGACTTCGACTTCTTTGTTGATCGCAAGCGAAGCTACAAATGGACT

TTTCAAAGGAGCAATTATTCAGAGTGGATCTTCTGTATCTAATTGGGCTA

TAAATAGGGATCCGTTAGCCATTGCAAGTCTTTTGGTAACAACATTAGGT

CATAAAACCAAGGATCCTAATGAAATGTATGAAATATTATCGAACCTAAC

TATTGAACAGTTGATCAAAATAACAGCCAAGAAAGATCTAGGTATGTACT

TTGATACTCAACTTTTACATTTGCCATGCGTAGAGGAACCGATACCGGGA

GTTGAACCCGCTATAACAGACATACCTTATAATCTTCTTAGAAAAAATAC

TAAAAATATACCTGTGATATACGGATCAAACACCAAAGAAGGTTTGTTTA

TAGCGATAGAAGAAACTGATGCAGAAATTGAAGAAAAGAACAAAAGATAT

ATATTCGCTTCTGACTTAGAGTTCCCATCAGAAGATAAGGCCCGCAAAGT

AGATCAAATTTTAAAAGAATTTTACTTTGGGAATGAACAAATAGGTCCAG

CAAATATAAAAGTCATATCTGATTTGTACACGCAACTATACTTTGAGCTT

CCAGAAATTTTTGAAGCTGATATTTTACTGAATGTTAGTGATGCTCCCAT

TTATAATTATATATTCAACTATTCTGGTGGAAGGAACTTCATGAAATCCA

ATCTTGGGTTTACTAATGAGTCTGGTGCACTTCACGGGGATGACCTATTG

TATCTATTTACTAGTCAAATATGGCCATATAGAATAAAAAAAGACGATCA

AAGAATGATTGACTGGATAACTGGAATGTGGACGGATTTTGCGAAATACG

GGGATCCGACGCCTACATCATCAAATGTGCCAGTCAAATGGATGCCTAGC

AAGAAAGGCGATTTAAAATTTCTATATATTGAAGATAAACTAAGAATGGG

TGCAATGCCCAACCCAGATGGATATCGGCTATGGGACGACGTTTACAAAA

AATATCGAATAATTAAACTCAAAAAGTAA

>CodbODE4

ATGCCGGCTTTTAGGTTATTTCTGTGCTTCGCCATCTGTGCCTTAGGTGC

TGGCCAGGGTTCGAACCCCATAGTTAGAGTGTCCCATGGGGTGCTGCAGG

GCGCTTGGCAGACCTACAAAGGGAAGGCTTATGCTAGCTTCGAAGGAGTG

CCGTATGCTAGGCCACCGGTCGGGAAATACAGGTTCAGGGAGCCGCAGCA

GCTGAAGCCCTGGCCCGGTGTATGGGATGCCACCAAGAAGCTGCCCATTT

GTCTGCAGTACGACCCAATCCTTGATCAAGTCCTTGGTAGCGAGAGCTGC

CTCTACCTCAACATCTACACTCCGAAACCGACCGCCGGCGCGTCCCTTCC

CGTCGTGGTGTTCATCCACGGAGGAGCCTTCATGTTCGGCGCCGGCTCCA

TTTACGACGTGTCGGCCATGATGCGCAGAGATGTGGTGGTCGTCACCTTC

AATTATAGACTTGGGCCGCTTGGTTTCCTGAGCACGGGCGATGAGGCAGC

CCCGGGCAATGCGGGGCTTAAGGACCAGGCGTTTGCGCTGCATTGGATCA

AGAACAACATCATGATGTTTGGCGGCAACCCTGACAGTGTGACCCTGACG

GGGTGCTCCGCTGGGGGCGCCAGTGTGCACTATCAGTACTTGTCGCCGCT

GTCTAAAGGTACTTTCCACCGAGGAATTGCCTTCAGTGGGTCTGCCTTTG

CCCCGTGGACACACGCCGTGAAGCCGGCGCAGAAGGCCAAGACCCTGGCT

TCCATCGTCGGCTGCCCCACCACCAGCAGCCGGGAAATGGTGGACTGTCT

CCGGTACAGACCTGCGGAGGTCCTTGTGAATGCCCAGATCGAGATGTATG

ACTGGAAAGTGCGCTACTTTACTCCCTTCACGCCGACGGCTGAGGCCCCG

AGCGTGCGGGAGCCCTTCCTGACGCAGTACCCTTTCCACGCGGCCCAGTC

CGGGGCCATGCACAAGGTGCCCCTCATCACCTCCGTCACCTCTGAGGAGG

GGCTGTATCCTGCTGCAGCCTACCAAGCGGATCCCGACATTCTTCCAGAG

TTGGAAGCCCGCTGGGAAGAGCTGGCCAGCAACATCTTCGAATTCAACGA

GACTCTACCTTTGGACCAGAGGGCTACCGTGGCTGCGAAGATCAAGCAAC

ATTACCTTGGTGGAAAGCCCGTCAGTCAGGCGACGTTTGCGCAGTTGGTC

CAGGCCTTAAGCGACCGTCACTTCGTGGTGGACGTCGGTAAGCTGGCTCA

GATCCACGCGGCGCGCTCCGGCCAGCCCGTCCACGTGTACAGATACTCCT

TCCGCGGGCAGAAGAGCCTCACTAACGTCGTAGCGCGTAGTGACAAGGAC

TTTGGTGTGAGCCACGCCGATGACGTCTTCCATGTGTTCAACCTGCCCGG

CACGGTGGGGGATTCGCCTGATGACGCCAAGATGACCGAGGTGCTTGTGG

ACATGATCTACAGTTATGCCGTCACAGGCGTACCTAGGGTAACCAACCAG

GGCCCAGCGTGGCAGCCAGTGATCCCCGGCTCCAGTGAGATCCGCTACCT

GGAGATCTACTCGCCAACCAAGACCGAGATGAAGACCAGCTCTGATTTCG

GACAGAGATCCTTCTGGGACAGCTTGGGTTTTATGGAGAATGAGAATTTC

CATTTGTATAGGAAAGACGAACTGTAA

>CodbODE5

ATGAGTTATACACGTGACAATCCAGAAGTGATACAAACAGTGGATGGACC

TATACGCGGTTATATTGACAAGATTGAGGATAAAAAATATTACAGGTTCA

AAAGTATACCTTATGCTAAACCTCCCGTTGGAAATTTACGGTTTTTGCCC

CCGTCACCGCCTCTACCATGGACAGACGTAAAAGACTGCACAAAGGATGC

CCCTTCGCCACATTCACACTTCCCCGTAGACATGAAGCTCGCACCGGAGT

CAGAGGACTGTCTGTATGTCGAAGTGTTTTCACCAGACATTGCACCAGAC

AAACCATTGCCAGTCATGTTGTGGACAGGCTGCTACCTGTTTGCGAGTAA

CATTGACGCAGTTTTGGATCCCACGCTTATCAACGATCAGCAAATAGTAT

ACGTGCGCTGTGGCTTTAGATTAGGAGCGTTTGGATTTCTCTCCACAAAA

GCCTTTTCGGCACCCGGCAACTGCGGACTTAAGGATATTGTCATGGCTTT

AAAATGGGTGCAGAGGAATATCAGCAATTTTGGTGGAGATTCCAATAACG

TGACCGTGTTTGGGAATAGCTCAGGTGGCGCTATAGTCCATCTACTAATG

CTATCTCCAATGGCAAGTGGATTATTCCACAAAGCTATCATGCAGAGTGC

AACTGCGTTGAACAACTGGTCGTTAGAGAAAAATCCTTTGCTACCACTGA

CTCAGCTGGCTAGGGAACTGGGTATCAAGAAAACTTGCAACGAAGAAACG

CTCCAAGAGCTTAGGAAAGTTTCAGCCGAAGACATAATGAACGTTGTCTA

TAAAATTGAGGAAAAACGGAGAATGGAAGACAGCGATACATTTGAATCAA

TATTCAAACCATGCATAGAAGAAGAGTATGAAGGACTACCAGCATTCCTC

ACAAAAACTCCTTGTTCAATAATAAAATCTGGTAATTTCAATAAAGTTCC

CCTAATAATCGGAAGTAATAACATAGAAGGTTCCGTTATGAAATACGTAA

AGAACGATTTCTATACTAATTTCGAAAAATTTAATGAAAACGTTCAGCTA

CTAGTACCTAAATGTTTGGCTGCAGAAAAGACACTGTCTAAGAAAATTGG

GCTTCAACTTCTCAAATTCTATTTAGGCGGAGACGAAGCGTTGACGAAAG

ATACGAAGAACCAGTACATTCAACTGTTAAGCGACTACTATTTCTTGTAT

TTCGTGAACAAGACTGTGAAGCTGCACAGTCAATTCTCTCCAGAGACTCC

TATTTACTATTACGTGATGAATTTTGTCGGCGAGTGGCTGGTTCCGGAGA

GACTCTCGTTTGTCAACAATCTGGGGCACTCCATGGAGCTGCCGTTCCTG

TTCGGCATCAGGTCGCCGGGTAGCCCGAGTGGGCAATTGTGCAAAGGCAG

CAGAGATTCCATTAACGTCAGAAGCAGTGTCATCAAAATGTGGACTAACT

TCGCCAAATATGGAAACCCAACACCCGATGAGGACGACCCCTTGGTTCAG

ATCAAATGGGATCCAGTGGAGAATAAGGAGAAACTGAACTACCTCAGCAT

AGCACCCGACCTGACCAAGGGCAGAAACCCGTTCCAGGATCGGATGGCTT

TCTGGGACGACCTGCATAGGGAGCACGCGGTGCTGAGGCTTTTGGTGTAC

TTCAACGACTTGGGCGTCTCGTGGTAA

>CodbODE6

TTGTTTTTAATAATGGCGAAAGTCCAAGTGGAACAAGGCTGGCTGGAAGG

TGTACAGTTGGAGACCTGCACAGGAGATGGGTCATATTACAGTTTTAAAG

GCATACCCTATGCAGCTCCGCCGGTCGGCAAGCTTAGATTCAAGGCTCCT

CAACCACCGTTGCCATGGCAAGGAGTACGAAAAGCGACAGAGCACGGCCC

AGTGTGTTGGCAGATTGATATTTTCACCAAGGAGCTCATCACTGGCAGTG

AAGACTGCCTCTATTTAAACGTCTATTCCCGAGACTTGAAGTCGAAGACT

CCAGCACCAGTAATGTTCTTCATACACGGCGGCGGTTACAAAAGCGGCTC

TGGAAACGACAACAATTATGGTCCAGACTTTTTGGTCAATCATGGAGTTG

TGTTAGTCACCATAAACTACCGGTTGGAGGCGTTTGGATTCCTTTGTCTG

GACACAGAAGAGGTGCCTGGCAATGCTGGCATGAAAGACCAAGTGGCTGC

CTTGAGATGGGTTCAAAAGAATATTGATAAGTTTGGAGGTGATCCGAATA

ATGTAACTGTATTTGGAGAAAGCGCTGGTGGAGCTTCTACTGCTTTGCAT

GTTTTGTCACCTATGTCTAAAGGCCTCTTTAAAAAGGCTATAGCAATGAG

TGGAGTTCCTACGTGTGACTTTTCTGTTGCCTTCCAGCCAAGGCGAAGAG

CGTTTGCACTTGGAAAACTACTCGGCTTCGAAACAGATGACCCTAAAAAA

CTTCTGGAGTTTCTCCAAAGCTTGCCAGCAGATAAACTGATTGACGTCAA

ACCTTGTGTAATATCTTCTGAAGAGACATCCGACAACATATTTAAAATGT

TCCATTTTACTCCAGTAGCGGAAAAACAGTTCGGGAAAGAGCACTTTATA

ACGGAAGAATCTTTAGAATTGCTGAAGAAAGGAAAGGTAAACGACGTAGA

TTTCATGTTCGGTCACACCAGCGCAGAAACTTTACTTGGTATCGAATTTA

TCAAACACTTGGGCACCAATTACCAAAAATATTCTGAAATTTTAGCCCCA

AGAAAGATACTAATCAATTGCGCACCAGCTAAAATACTAGAAATAGCCGA

TATGATCAGGAAACAGTACTTCGGCGATAAAAATTTTGGCTATCCAGACA

CTTTAAAGGAAACCTTGAACTATCTATGTGATACTTGCTTTACATACGAT

GTTTCCAGATTCGGAATACGTTTCCCAAAGACAGGAAAGAAGGTATTCAT

GTATAAGTTCTCATGCGTGTCCGAGAGGAATATTTATGGCAATGTTGGGA

AGGAACATGGGTTGATTGGAGCCAGCCACTTGGATGACCTGATGTATCTG

TTCCATCCGTACAAAGTAAACCTTCCCATAGACAAAAAGAGTAGGGAATA

CAAGATGATTCAACAGACTTGCACATTGTTTACGAACTTCGCCAAATATG

GAAACCCAACACCAGACTCATCGCTTGGCGCAATATGGCCTGAGTTCGAC

AGTGTCTCACAACGCTACTTGGACATCGACGAGACCCTCACTCCTGGACA

GAAGCTCAATTCAAATGTTGTGGACTTCTGGAGCAAGATCTACAAGATTG

CTGGGCTGGACTATTGA

>CodbCXE1

ATGCCGCCGCCGCCGCCACCGGTTGTGGAGACAGAGCAGGGCACAGTCCG

TGGTATTGTTATCGAAAATGGAGAGTCCAAATACGAAGCATTCAAGGGCA

TCCCTTACGCCAAACCACCTCTTGGAAAATTAAGATTCAAGGCGCCCGAG

CCCCCGGAGGCATGGCGGGGGGTGCGCGAGGCGACGCAGCACGGGCCCGT

GTGCCCGCAGTACAACGAGCGCATGGACCGCATCGAGCCCGGCAGCGAGG

ACTGCCTGCACCTCAACGTGTACCGCCCGCCGCGCCCCCCGCGCCGCGCC

GACCCGCTGCCCGTCATGGTCTGGGTCCACGGCGGCGGCTTCTACACCGG

CTCCGGCGACTCCGACTTCTACGGCCCGGAGTTCTTCGTGGCACAAGAAA

TCATACTGGTCACTTTCAACTACAGGTTGGAGGTCCTCGGCTTCCTCTGT

CTCGACAATGAGGACGTACCCGGCAACGCCGGCCTGAAGGACCAAGTGGC

GGCCTTGAAATGGGTTAGAAAGAACATAGCAGCCTTTGGTGGCGATCCAA

ACAATGTCACTCTCTTCGGCTGCAGCGCCGGCGCCGCGTCCACTTCTTAC

CACTTGTTGTCGGACATGAGCAGAGGACTGTTCCACAAAGCCATATGCCA

GAGCGGGATTTGCCTGAGCGAGTGGAGCTACAATCTGCACGCACGCCAAC

GTGCCTTCCAACTAGGAAAGTTCCTGGGCAAGGACACCGAAGACCCACAA

GAGTTACTAGAGTTTCTACGTAACGTGCCTGCGCGTTCGCTAGTCAATAT

TAAATTGCCGTCCTTAGAAACTAAGTTTCAAGATATAGCGGATAGTATAT

TATTCGGACCTGTGATAGAAAAACCTAATTTAAACGTAGAGAAGTTTGTA

TCGGAAGAACCACCTAAGCTCGTGAGAGAAGGCAGAATAGCGAACATTCC

TTTAATTTTAGGCTACACTTCGGCAGAAGGAATAGAGATAGCTAGAAAAT

TGTCACATATTCTTGGATTTATGAAAACTGTAGGCGCAGTAGTGCCAAGA

GAAATAAAAATGAAACTGTCACCAGAAAAACTAAAGGACATAGATGAAAA

AATACGTAAAAATTATTTCGGTGACAAAGAAATTTCAATGGAGCTTATAA

AGGAAGTAGTAGACTTTGAAACTGACTGTTTATTTTCATACAACATCCAA

AGATATGCGCGATACCATGTTTTACATAATACGGAACCGGTGTACATGTA

CAAGTTTACAGTTGAGACTGAGAGGAACTACACAAAGACACTGTATGGAA

TGGAACACATAGAAGGGGTTTGCCACGCCGATGACTTGTTTTACTTATTT

AACGTCACGTGTCTCGACGTGCCACTGACCAAAGAATCCAAAGACGTAAT

TAAGAACGTTGTACAACTATGGACTAATTTCGCCACCACCGGAAACCCCA

CGAGTTCGCCCGATATCGAACAATGGGTGTCCTTCGAGGAAACGGAACGA

AGTTATTACACAATCGGGGAGAGCTTTACACGCGGCACAAATAAGGAAAA

CAAAAACATGCAGTTCTGGGAAGAAATATACAACGAAACTACAATTGAGC

CAAGAAATAATAATATAAATACGGGACCCTAA

>CodbCXE2

ATGTCAGAAAACAGGCAGGAAGTTTTGAAAGACGCTGCCGCAGCAGCGAC

GGCGGCCGCGGCGGCAGGCGGTTCGCTGTTCAGCACGTTTGATATCGTTA

TGCTAGTTTTATTACTGGGTGCCGCTGTGTGGTGGCTCTACAGTTCTAGA

AAAGAAAATAAAAACGATGAACTTCTGCTGAGTAAATATTCCATACAGCC

AGCAGGATCGATACAAGTCACCGAAAATTCTTTTATAAAAAAGTTACAGT

CGTCCGGCAGAAGTTTAGTGGTATTCTATGGCTCTCAAACAGGGACTGGT

GAAGAATTTGCTGGTCGGCTTGCCAAAGAAGGCATACGATATAAGTTGAA

GGGAATGGTGGCTGACCCCGAGGAATGTGATATGGAAGAGCTTATAAAAC

TCAAAGATATATCAAATTCATTAGCAATATTCTGTGTGGCGACATATGGT

GAGGGAGATCCTACTGATAATGCGATGGAGTTTTATGAATGGTTGAAGAA

TGGAGAGCCTGACTTAACTGGATTAAATTATGCGGTGTTTGGATTAGGAA

ATAAAACCTATGAACACTACAATGCAGTGGCTATTTACATAGACAAAAGG

TTAGAGGACCTCGGAGCCACTCGTGTGTATGAACTGGGTCTGGGCGATGA

CGATGCTAACATTGAAGATGATTTCATCACGTGGAAAGATAAGTTCTGGC

CGGCCGTGTGTGAGAAGTTCAGCATCGAGAGTGCGGGCGAGGAGGAGCTG

GTGCGGCAGTTCACCCTGGTGCGCCACGCGCGGGGCGAAGTGCCCGCCAG

CAACGTGTTCACGGGGGAGATCGCCCGCTTGCATTCACTGCAAGTCCAGA

GGCCACCCTATGATGCCAAAAATCCATTCTTAGCTCAAATTACAGTAAAT

AAAGAACTACATAAGGGGGGTGATAGATCTTGCCTCCATGTGGAGTTCGA

CATTTCTGATTCAAAAATGAGATATGAAGCTGGTGACCACATTGCGGTGT

ATCCTATAAACGACACTAGCCTGGTGGAGCGGCTCGGCGAGCTGACGGGC

GGCGACCTCGATGAGATCTTTTCGCTGGTGAACACGGACCAGGAGAGCAG

CAAGAAGCACCCCTTCCCCTGCCCCACCGCCTACCGCACCGCGCTCTCGC

ACTACGTCGAGATCACCGCGCTGCCTCGCACGCACATCCTTAGAGAACTA

GCCGAGTACTGCTCAGATGAAGAGGACAAGAAGAAGCTGCTACTAATGGC

GACCAACTCGCAAGAAGGCAAGGCGCTGTATCACTCGTTCGTGGTGGACG

CGTGCAGACACATCGTGCACATCCTGGAGGACATCAAGTCGTGCCGCCCG

CCGCTGGACCACCTGTGCGAGCTGCTGCCGCGCCTGCAGCCCAGATACTA

CTCCATATCTTCCAGTCCTAAGCTGTACCCGGACACGGTGCACATCACGG

CGGTGGTGGTGCAGTACCGCACGCCGACGGGGCGCGTCAACAAGGGCGTC

ACCACCACGTGGCTGGCGCAGCACAGGCCGGAGCCCGGCGCCGGCGGCCC

GCTGCCGCGCGTGCCTGTCTTCATCCGCAAGTCGCAGTTCAGACTGCCGC

TGCAGACGACGACGCCCATCGTGATGGTGGGGCCGGGCACAGGGCTGGCG

CCGTTCCGCGGCTTCCTGCAGGAGCGCGCCTTCGCCCGCGCCCACGGCAA

GGAGGTCGGCGAGAACGTTCTCTACTTCGGCTGCCGGAACCGAGGCCACG

ACTTCATCTACCAGGAGGAACTCGAAGGATATGAAAGAAGTGGCGATGTG

AAATTGCACCTCGCGTTCTCCAGAGACCAAGAACATAAGGTGTATGTGAC

ACATCTCCTTGAGCAGAACATGGACCAGATTTGGGACATTCTTGGAAATA

GAAATGGACATTTCTATATTTGCGGTGACGCAAAGAACATGGCCGTGGAC

GTGAGGAACATCATTCTAAAAGCTTTACAAGAAAAGGGTGGTAAAACAGA

AGCCGAGGCGGTACAGTACCTCAAGAAACTAGAGTCCATGAAGAAGTATT

CTGCAGATGTGTGGAGTTAG

>CodbCXE3

CTGTGCGCCTTCGTTGCATACACAGATTGCGTTGCCTGTGTGCAAACCCA

GTGGTCCAACTACCGGGATGTGAAGACACAGGAGGGGATCGTGAGGGGCA

CAGTGGGGTCAGATTATGTATCGTATCTCGGCGTGCCATATGCTGCCTCT

CCCATCGGGGAAAATCGATTTAAAGATCCAATACCACACCCTTTATGGGA

GGGCGTCTACCATGCGAACCGCACAGTAAAGTGTCCTTTCAACGGAGATG

GGGATGAAGATTGTCTAGTCCTAAACATATTCACACCAATTGATATCAAA

GACACGCCAGTACTCATATACATCCACGGTGGTTCCTTTATTATGGGTTC

AGGGCAAACCACTGGAGTAGAACATCTAATAAAAGAGAACTTGACAGTAG

TGACTGTAAACTACAGGCTCGGAGTATTAGGGTTCCTCTGTCTAGGTACT

GAAGATGCCCCAGGAAACGCTGGACTGAAAGACCAAGTAGCTGCTTTCAG

ATGGGTACAGAGAAACATCGTCAATTTCGGGGGTGATCCAAAACGGGTAA

CGATCTATGGTATGAGCGCAGGAGCTGCAAGCGTGGAATTTCATGTTCTG

TCAAACATGAGTAAAGGTCTTTTCAAATCGGCCATTGTCGAGTCTGGCGC

TGCTTCTTCCGTTTGGACAACAGACCCTGATCCAATTAGTACAGCGATTA

CCTATGCAAAAGTTTTGGGAATGCCTGAAACAAGTAATTTGACCAAACTG

GTCCAGTATTACAAGGAAGTGCCGGTAGACAAACTGAGCATAGTAAACTA

TGATTACTACAACAGCTTAACTGATGGATATTTCGGTTTCGTGGCTTGTG

CAGAGAAAGATATTAAAAAAACAGAGGCTTTCATCACAGAGTCTCCTTAT

GAAATGTTGAACAAAGAAGATTATGACAAAATTCCTTTGATGATTTTCTT

TTCAGCCCTAGAAGGTTTGTTTCTGCGAAGTGAGGATTATTACACACTGA

ACTACAAAGAAAGAATGGAAGAACACTTTATTGATTTCATACCTGGCGAT

CTTATCTTCGAATCTCTAGATATCAAGCAGGATGCTGCTCAAAGTGTAAA

GCAGTTCTACTTCGGCAATGACAGCATTGGCAACAACAAGATAATAGAAT

ACTTGAATTACTTCGGTGATAATCTTATCGTCAATGGTTTACTGAAATCT

GTTGAAATACACTCAAGGAACCAAAATCCTGTGTATTTGATGGAATTCTC

GTATAAAGGCCAGATGGGTAGCCATGAAGAATTTTATGAGAATATCACTC

TAGCAGGCCATGGAGATGTAGTGAAGCACGCTATCTTGCTCAGATCGATA

CAGAATGAGGATGACAGGAGAACGGTGGAGCGAGTGGCTAAACTTATTGC

GAATTTCTTAAAGTTTGGGAACCCAACACCAAACAAAACGGATCTACTAC

CCGTGACCTGGCCAACCTACCAGCCAGATAAACAGTATCTTCACTTGGAC

AAGGAGGCTCAAGTTCTGGAGCTATCACAGCACCCGATGATGACCAGGGT

GAAGTTCTGGGATGAGCTGTATCAGAAACACAGGAGGACTATAAGGCCCG

TTGACCCTAGTCTATACGTGACCAGTGGTGGGTACAGTATTCGATTCAGT

GGAGTGATTATACTATTCTTTTACACAATAAGTATTTTTGATTAA

>CodbCXE4

TTGCGTAAAGAGACTATCCTTGCTGATATTACTATTGACAATAGCTCATG

TTTCAGAAACCAGCACCACATCCAAGATGCTGTCGCATTTTTCTACAAAC

CTTTGCCGCCTACGACGAACAAAACGTTATTGAGAAATAAGTTCCTTGAG

TTTTACGGCGACAAAGTTTACGGAGCAACCTCTTATCAGTTGGCGAAGCA

GATGAGTAAACAA

>CodbCXE5

ATGAAAGACCACGTGGCGCTGCTCCAGTGGGTGAAGAGGAATATCGCTCG

TTTTGGCGGCAATCCCGATGACGTCACGATCCGAGGTATCAGTGCAGGGG

CTAGCTCCGTCAGTTTGCTTACGCTCTCCAAAATGGCACGAGGACTCTTC

AGTAAAGTAGTAGCTGAGAGCGGTTCACCTGTTGCTGTTTGGGGGGTGCA

AATATCTCCTGTAGAAACAGCATTTATTTATGCCGAAAAACGTGGTTTCA

AGAATAAATCAAATGATATCTACGCCCTTGAAGAGTTTTATAAAAATTTA

AATTTAGAAATACTTAATGACGCGTTTGCATATAATGGTGATTTTACCTT

TTGGGGGTTCGTACCATGCGTGGAACGCCCAACCGGCGGCGGCGGAGGCA

TGGTGTTCTTAGATGATGCTCCTGTTAACATTATGAAAAGCGGTGATTAC

GCTAAACTACCTACTTTAATGAGCACTTCAGCCAGTGAATTTGATAATAG

ATTTGATTTTACTAAACTTGATTTACCAAAAGATTTGCAGTTTAAAAAGA

AAAAAGAAAGAGAAGAGTTAAATCAAAGAATCTCGAAGTTTTATTCTAAT

TATAAAGATTATGTAAGCGATATCTCATACACTTACTCAATGCTAAGAGA

AGTAAAGCTGCACATTGAAGCTGGCCATAACAAGTTGTACTTTTACGAGG

TCGGCTACGACGGTCACGCCGCCCAGGCGTGGACTATTAGGGATGAATCT

GAAAATCAACGTGAAGATACACTTGAAAAAGATTATAAAAGAGTTAAAGC

CGTTTTGCGTGAAATATTGCTAAACTTCGTAACTGCAGGCAACCCTGTGC

GACAGGGCTCGGGGATGCCGGCGTGGGGGCCCGTGGTGAGCACAGAAGGA

GCCCCCCACATGCTGTTGGAGCAGGGCGCCGCACCGCGCATGCGCGGCCC

TCCGCCACAGGCGGCGCGGGCGCAGTTCTGGCACGCCGTCTACGACGAGC

ACTACCGCCCGCCGCAGCCTCCGCCCAAACCTCCCTCTGCAGTTACAGTG

ATGCGACGAGTATTCCAAAGTGACTACTGGCATTATGCTGACGTTTCAAA

ACATGTGAATTAA

>CodbSNMP3

TTGCGTCACGAGTACGACTTGCAGTACGAGGGCATCCCGGTGGGGCGGTT

CGTTCTTAACGAGTGGTTTTTGGATAACGACGAAGGCTGCTTCTGCCTCA

ACGTCACGCAGGGTCCCGGCCTCAACCGAGAGGACGGCTGCTTGCTGAAA

GGCGCCATGGAACTCTACACGTGCGTTGGTGCGTTTTTGGTTTTGTCTTA

CCCTCATTTTTTGTTTGCTGACCCTATATACCAAAATGGAGTTTTGGGTA

TGAATCCAGTGAGAGATGATCACAGAATATTTTTAGACATAGAATTGAAT

ACAGGAACAGTGTTGAGAGGTGCCAAAAGGGGGCAATTTAACATAATGAT

GCGTCCTATCAGAAGCATAGCCGCTACACAGAACCTCCTCCCCACATTAA

CACCCTTAGTATGGTTTGAAGAAGGCATGGCTCTTCCCGAAGAATTTGTG

GATGAAATAAAGGACCGACTGTTAGCACCACTGAACCTGCTGGATATTTT

GATACCCGTACTGATTGCTGTGTCCTGTGCCGTGCTCCTGGTAGGAGTCG

TATTAGCTGTAAGAGTAAAGCTG

>CodbSNMP1

CGCATGATGCAGTTGCCCAAGCACCTAAAGATAGCTGCGGGCTGCGGCGG

CGCGGCCGTCTTCGGCGTCCTGTTCGGCTGGCTCTTGTTTCCAGTCATAT

TGAAAGGGCAACTCAAGAAGGAAATGGCTCTTTCTAAGAAGACGGATGTA

CGGATGATGTGGGAAAAGATACCTTTTGCTCTCAACTTTAAAGTCTACCT

GTACAACTACACTAACGCCGAGGACGTAGCAAAGGGAGCTCTGCCGATTG

TCAAGGAAATTGGACCATACCACTTTGACGAATGGAAAGAGAAAGTAGAA

ATCGAAGACCACGAGGAAGATGACACGGTCACGTACAAGAAGCTGGACGT

TTTCCACTTCAGGCCAGACCTCTCGGGCCCAGGGCTGACTGGGGAGGAGG

TGGTTGTGATGCCTGATCCGTTTATATTGGGTCTAGTTACGGTAGTGTCC

AGGGATAAGCCAGCCATGCTGAACATGATCGGCAAAGCCCTCAACGGCAT

CTTCGACAACCCAAAAGACATCTTCCTAAGAGTCAAGGTTCTAGACATCC

TGTTTAGAGGGGTCATAATAAACTGTGCACGGAGTGAGTTTGCTCCTAAA

GCCGTCTGTACTGCGTTGAAGAAGGAAGCTGTCACTGGGATGGTCTATGA

GCCTAACAACCAGTATAGATTCTCTTTGTTTGGAACACGCAACGCAACCG

TAGACCCTCACGTGATAACAGTCAAGAGAGGCATTAAGAACGTGATGGAC

GTCGGCCAAGTGGTGGCTGTGGACGGCAAGCCGCAGCAGGACATCTGGAG

AGACTCCTGCAATGAGTACCAAGGCACCGACGGCACCGTCTTCCCGCCCT

TCTTGACCGAGAACGACAGACTGCAGTCGTACTCTGGTGATTTGTGCAGA

TCCTTCAAGCCGTGGTATCAAAAAAAGTCGTCTTATCGCGGCATCAAGAC

GAATCACTACATTGCTAACATGGGCGATTTTGCTAACGACCCAGAACTGC

AGTGCTTCTGTGAAGCCCCCGACAAGTGCCCCCAAAAGGGGCTCATGGAC

CTTACGAAGTGCATGAATGCTCCCATGTTCGCCTCTATGCCGCATTTTCT

GGACAGTGATCCAGAGTTGCTGGCAAACGTGAAGGGACTGAACCCAGATA

TTAACCAGCATGGGATTGAGATTGATTTTGAGCCGATATCCGGAACCCCG

ATGGTTGCTAAGCAACGAGTCCAGTTCAACATGCGCCTCCTGAAAACCGA

AAAGCTGGAGCTGTTAAAAGACCTGCCAGAAATCATTGTACCTCTGTTTT

GGATTGAGGAGGGGCTCGCCCTCAACAAGACGTTCGTAAACATGCTGAAA

CACCAGCTGTTCATCCCGAAGCGGGCCGTGGGCGTGCTGCGCTGGTTGCT

CGTGTCATTTGGCACTTTAGGTGCCGTCGGCTGCCTCATCTTCCACTTCA

AAGACCGCCTAATGCAGTTCGCAGTGACATCAGGGTCGGCGGAAGTGCGC

AAAGTGGCCCCCGGGGAGCTAGCGGTGGAACAGAAGGAGATAAGCGTCAT

CGGGGATCCAGATGCCCAGGAGCCCACGAAGATCACCATGTAA

>CodbSNMP2

ACAATTAGTTACGTCAAAATGTTGGGGAAGTATTCCAAATTGTTATTTTT

GATTTCTCTCATTGTGACAATCTTGGTTGCCGCTACAGCTATCTGGACTG

GACCGTACATCGTCAAGAAACAAATTCAAAAGAGCGTTCAGCTAGACAGC

TCATCTCAGATGTTCGACAAGTGGAAGAAACTTCCTATTCCCCTGAACTT

CAAGATACGGGTCTTCAATGTAACGAACCCTGATGAAGTTAATAGTGGCG

AAAAACCTAAACTGGCTGAAATTGGACCCTATGTTTATAAAGAGTACCGG

GAGAAGAGAATCCTCGACTACGGCGAAAATGACACGATAAGGTATATGCT

GAAGAAGAGTTACTTCTTCGATCAGGAGCAATCTGGCTCCTTGACAGAAG

ATGATGAAGTCACTGTAATCAACTTCTCTTACTTGGCGGCTATACTAGCA

GTGGAGGAGATCATGCCCAGTTTCGTGGGGACAGTGAACCAGGCGTTAGA

ACAGTTCTTCGAAGATCTGCGGGACCCATTCCTGAGGGTCAAGGTTCGAG

ATCTGTTTTTTGATGGGGTGTTCCTCAACTGCAGTGGAGATCATTCTGCT

TTGGGCCTCGTTTGCGGTAAGATTAAGTCCGACACGCCACAGACCATGCG

ACCAGCGGAAGATGGAACGGGGTATTTCTTCTCCATGTTTTCTCATCTAA

ATAGAACAGAGTCAGGCCCATACGAAGTCGTCCGCGGGAGGGACAACCTT

TACGAGCTTGGTCGCGTAGTATCCTACAAGAACAAAACCGTGTTGAACAA

CTGGAGGGACCCATATTGTGGACAAATTAACGGAACAGATTCGTCCATCT

TTCCGCCTATAGATCAGAGTAACGTGCCTAGGAGATTGTATAGCTTTGAG

CCAGATATATGCAGGTCTCTTTACGTGGAATACGTTGAGGAGAGGTCCAT

TTTCAACCTGTCAGCTTATTACTACGAGATACCGGAATCAGCTTTAGCGG

CAAAGAGCGCCAACAAGAATAACAAATGCTTCTGTAGAAGAAACTGGAGC

GCAAATCACGACGGTTGCCTAATCATGGGTGTCCTCAACCTGGCTCCGTGTCAAGGAGCTCCCGCCATCGCCTCCTTCCCCCACTTCTACATGGCTTCCGAAGAACTCCTGGAATACTTCAATGGAGGAATCGAACCCGAGAGAGAAAAACACGCTACTTATTTGTACTTAGATCCGCTTACAGGAGTTGTTTTGAGAGGTGCAAAAAGGTTACAGTTCAACATTGAATTGCGAAACATTAAAGGAATTCCACAGTTGGAGAATGTTTCCACTGGATTATTTCCACTGCTTTGGATTGAAGAGGGAGCAGAAATTCCTGTTTCAATCGCAGAAGAGCTCCAAGCCTCCCACAAGTTAGTGAAGAACGTCAAATTGCTGCCCTGGATCTTTTTTGGAATTTTTGCACTTGCTACGGTTGCAAGTGCGTGTGCTGTACTCAGAATGTTACCTTTCTTTAGAAAACACACGAATTCTGTCAGCTTTATACTAAATTCTGACGCTAATAAAGTGAGAACA

>C*obdPR1*

ATGTTTCTGATGGTGCAAATGTATTTTGGTTGCACGATGTTCAACCTTAT

TCCCTTCTACAGAAGCCTGAACGATGGCGCGTTCCGCAACGATAAGCCCG

CAAACGTAACTTTTAAACATTGTGTATACTTAGAACTACCTCTTGATTAT

GAACACGACTTCAAGTCCTACGTTTACGTGGTTTTCCTGAATGTGTATCT

TACCTGCTTGTGTTCCACGCTTTTTTGTACAGTTGATTTGACGATCGCCT

TGATTGTTTTGCACCTATGGGGTCACTTAAAGATTCTCCTTCATGATTTG

GAACACTTCCCCAAGCCAATCATTGATGGGCCAAGCAGCAAGTTTGCAGA

TCATGAGATGAAAAAAGTCTTCAGTCATCTGATAGACATCATCCAGCATC

ACAAATTCATTTTGGAGTTTACCCAACAATTATCTGATACTTACAGGCCA

TTTTTAATAATATACCTGGCTTTTCATCAAGTAGCTACATGTATCTTGAT

GTTGGAATGTTCGCAAATGACAGTAGATTCGCTCCTGCTGTATGGACCCC

TAACCATTATTTTGCTACAACAATTGATTCAGTTGTCTGTTGTTTTTGAA

TTAATGTACTCTACGAACGAGAAGGTCCAAGATTATGTCTACAAT

>CodbOR2

ATGATACAAATACTATTCCACAATTTTTCGAGGTTATGTCCGGGTTTTCA

CTGTAATTTTCGTGTTGTGGTAGTGTTATGTATGATATCGGGAGACCTGC

TTTTCGTGATATTTCTGAGCCACCTCAGCACACAGTTCGACCTGCTCAGC

GCCAGGGTGCGCCGGAGTATCTTCGTGCCGATAGACTGCCAAATGATCAA

GGAGTATCCTCTCGGTTTTTACAGTGAATATTTC

> C*obdPR2*

ATGGAAACTGACTTAGACACAGTTGGAAAACTGAAAAAACGCGACTCTAT

TTTATCGATAAAATACATAAAAATAGTTCGTTTTTTTCTCACAACTATAA

CTGCTTGGCCACGCAAGGAGTTATGCGAAAATTCGACCAGAAGTCTACTG

TATACGTTGAAAATCTACCGCTGTTTTTTGCTTGTATTCGAGGTTTCCAT

GACAGCCTCTTGTATATATTATCTTTGTGTTACTGATTTAGATATGATGG

GCTTTGGGCAAAATTTTATAGGTAGTTTTCTTACTTTTATGACAACGACT

CGACTAATATTGACCGGTTTTAAAGAATATAAGAGTATCATTGAAGAATT

TTTGATGAAGTTTCATTTATCACATTTCAAACATAAGGGCTCAATCTTCG

AAGAGGTCTACAAAATCATAGAGAAAGCTTCTTATTATTTCACAATGTAT

ACATGTTACAGTATGATTTTTGGATGTACCATGTTTAATTTAACACCCTT

CTACAACAACTACAAAAGCGGCGCCTTTTCCAAAGAAACTGCCAACGTTA

CCTACGACTACAGCATGTTCTTGGCGTTTCCCTACATCGATACATCCAAA

TATTTCAGATCGATCACTATTATAAATAACTATATATCTTGGAACTGCAC

TATAGCCATATGTGTTATTGATTTGTTCCTATATATAATGGTATTCCATA

TTATTGGCCATATAATAATTTTGAGAAAAAGTCTGGAAAAAATGGAAGGG

CCACAAACATCTAAAGCCACGGAAGGGACTAAAGAGTGGGAAATAAGAAG

GTACGCCGTGAAATACAGCATTTCGGAGAACAATAATATTGCCGGGTTTA

TTGAAGATTTTGTCAAACATCATAGATTAATAACTAGTTTCACAGAGCGT

ATGTCTCGCTTCTTCGGGCCTGTGCTGGCGATGAACTACGTGTTTCAGCT

GACCAGCCTCTGCATATTACTGCTGGAGAGTACGCAAGGTGACTTGGGCG

CTATGATTCGCTTCGGTCCACTGACGGTTATTATTTTCTGCCAGCTGATA

CAAATATCAGTTATATTTGAAATTGTCGGCACTGTTAGTGAGAAACTCCC

TGATGCTCTATACTCGATCCCGTGGGAGTGCCTGGACATCAGGAACCAGC

GCTCCGTGTGCATCCTGCTGCAGCGGCTGCAGCGCCCCATTAACGTCACC

GCCTTGGGCATGACCACCGTCGGCGTCACTACCATGGCTGGGATTTTGAA

GACCACATTTTCTTACTTTGCGTTTTTGAGATCCCTAAATGAATAG

> C*obdPR3*

ATGGGGTGTATCATGTTCAATATCTCACCATTCTACAACAATTTCAAGAA

CGGAGCTTTTTCCAGAAATCCGCCTGCTAACATTACCTACGACTTCAGTG

TATTCTTCAAATTCCCCTTTGTCGACCCATCCGACTTTTTCAGAACAGCA

ACTGTTGTTAATATGTATTTCTCCTACAACTGTGCCGTGTCGGTGTGCAT

AATTGATTTGTTGCTGTCACTAATGGCACTGCATATTATCGGTCATATCA

TAATTTTGAGAAAGAGTCTGGAAAAAATTCAAGGCCCACAACATTGTAAC

GTAATGGAAGTAGTGGAAGAGACTAATGAACACTGGAAAGTAAGAAGATG

CACCGTAAAATACAGCATTTCGGAGAACGATAATTTTTTCGGGTTTATTG

AAGATTTCGTCTTACATCATAGACTTATAATCAGTTTCGCAGATCGTGTG

TCTCGCTTTTACGGGCCCGTGCTGGCGATGAACTACATGTTTCAGATGAT

CAGCCTCTGCATACTTCTGCTGGAG

>CodbOR5

ATGTTTATGTATGTTGTCATGTGTTCACTTATGCTGTGTGCCAGTGCTTT

TCAACTAACGTCTGCTACAAACGCGACCCAAAAGTTACTCATGGCTGAAT

ACTTGATTTTCGGTGTCGCCCAACTCTTCATGTTTTGTTGGCATAGCAAT

GATGTTTTATTAAAGAGCGAAAACGTATCTTTGGGTCCTTACGAGAGCGA

ATGGTGGGCAGCTAGCGTACGTCAAAGGAAAAGCTTATTCATCCTCTCAG

GACAATTTCATATCAGTCATATTTTTACTGCTGGGCCTTTCACCAATCTG

ACGTTATCGGCTTTTATAACAATTTTGAAAGGAGCGTACAGTTATTACAC

TCTACTAAGAAAATAA

>CodbOR6

ATGAGAATGCGCACAATTACTTACTTACAACTGAGTACTTACTTACAACT

GAGTACTTACTTACAACTGAGTACTTACTTACAACTGAGTACT

>CodbOrco

ATGATGACCAAAGTAAAGACCCAGGGCCTGGTGTCCGATCTGATGCCCAA

CATAACTCTGATGCGGATATTTGGACATTTCTTATTCAATTACTTACCAG

AGAGTGGTGGTATGTCTCTTCTCCTACGGAAGATCTACGCTAGCACTCAT

GCCTTCTTAATTGTCATAAACTTCGTCTGCCTCGGGATCAATATGGCTAA

ATACTCTGATGAGGTCAACGAACTCACAGCCAACACCATTACGGTCCTGT

TCTTCACCCATACCATCATCAAGCTCGCTTTCTTCGCCATTAACTCCAAA

AGCTTCTACAGGACCCTGGCAGTGTGGAACCAGTCAAACAGCCACCCGCT

ATTCACGGAGTCAGACGCGCGCTACCATCAGCTCGCCCTCACCAAGAACC

GACGGCTGCTTTACTTCATCTCCGGGATGACTGTCTTTTCTGTTATCTGT

TGGATAATCTTAACGTTCTTCGGAGAGTCAGTGTACCACATAATGGACAA

GGAGGCTAACATGACTATAACGGAGCCAGCCCCAAGGCTGCCCCTAAAGG

CCTGGTATCCATTCAACGCTATGAGTGGAACCATGTATATGGCAGCATTT

GCTTATCAGGTGTACTGGCTCCTCTTCTCGATGATGACTGCTAACCTGCT

TGACGTGATGTTCTGCTCCTGGCTTGCGTTCGCATGCGAGCAGCTGCAAC

ACCTAAAAGCCATTATGAAGCCTCTCATGGAACTTAGCGCTTCGTTGGAC

ACATACCGACCGAATACTGCTGAGCTGTTCAGAGCTAATTCTACAGAGAA

ATCCGAGAAAATCCCAGACCCAGTGGACATAAATATCCGCGGGATATATT

CCACGGAGCAAGACTTTGGCATGACACTGAGGGGAGCTGGTGGACGTCTC

CAGATCTTCGGACAGAACAACACTACCAATCCCAACGGACTGAGTCAGAA

ACAGGAGATGTTGGCACGATCAGCTATCAAGTACTGGGTGGAGAGACACA

AGCATGTCGTGAGACTGGTGGCGTCCATAGGAGATACCTATGGAACTGCT

CTGCTGTTCCACATGCTTGTCTCCACTATTACCCTCACACTGCTGGCCTA

TCAAGCGACGAAGATCAATGGGCTAAACGTATATGCTTTTAGCACAATCG

GCTATCTGAGTTACACGCTTGGACAGGTGTTCCACTTCTGCATATTCGGC

AACCGGCTTATTGAGGAGAGTTCATCAGTTATGGAAGCGGCATACTCCTG

CCAGTGGTACGACGGATCTGAGGAGGCGAAGACCTTCGTTCAGATAGTCT

GCCAGCAGTGCCAGAAGGCCATGAGCATCTCTGGAGCCAAGTTCTTCACC

GTGTCACTGGACCTGTTTGCTTCTGTACTTGGAGCTGTCGTGACCTACTT

CATGGTGTTGGTGCAACTTAAGTAA

>CodbOR7

ATGTTTTGTTGGCACAGCAATGATGTTTTGATGAAGAGTGAAAACGTTTC

TCTGGGTCCATATGAGAGCTACTGGTGGGCTGCCAGCGTTCGTCAAAGAA

GATACGTGCTCGTTCTAATTGGGCAGCTTCGTATCAGCTACATGTTTTCA

GCTGGACCATTCACCAATCTCACATTGTATACGTTTATAACAATTTTAAA

AGGAGCATATAGTTACTACACTCTACTAAGAAAATAA

>CodbOR8

ATGGTATTGGTATTCTGTACTCTAGGCCAACAAGTACAAAATGAAGTAAG

TACTATTCATGATTTCAATCAAAAAATTTAG

>CodbOR9

ATGGCATCAAAAAAAGTTTTTGTATTAATAACCCGATTTCTTGCTGACCG

TACGTTTTTAAAATCTGTCAATATGGACCAAATTTCCTCCAAAGTTAAGG

ACGGTCTCCTAACACCGTTTTCCTGTAGTGCCAATTGTTAA

>CodbOR10

ATGTGTGAAAAAATTGAGCAAAACGTCCTGGAGAAATGGTACATCTTCAA

TCGGGCACACAAAGTAAATATGCTCATCTTCAAAATAGCGCTCGACCAAA

GAATGCCTATCTATATTTTTGGATCTATAACACTATCTTTGCCTACGTTT

ACATGGTTTATCAAAACGGGAATGTCTTTCTTCACTTTAGTAATGTCATT

ACTTGAAGAAATAAAC

>CodbOR11

ATGGAAAACCATAGTCCTCAAGAACGGACCGTACCGATACAGTATGTGCG

AGGATCGCGGGCTTTCCGCCAGTTCAAAAACCCTCCACAGCCTCATATGT

GCATACAAGACACGATAAAGGACACTTCTGAAAAGCTCTTCATCAACGTG

CTGGGATGGCAGAAAATCGCGAATCCGAAGCATTATTCAGATCCCATACC

GTTGTATGGGGGTATGCAGGTCCCTCAAGGCTGTGGGCCAAACAGCAATC

GTCCTCCACTGCTGGTGTTTGCTGTGATGGTGAACCCTGATATCCTGAAA

GCCAATGGGAAAAATGCTGTCAATCCTACAGATCGTGAGGCACTTGTGAG

TCTGCTCTGCGACTTTGTAGAAGCCATGAATCCAGGCCTAGTTTTGGAAC

GGAACCCCGTTATCCTGAGGGACCGTGACCTGGCTGGAGAGTTGAAGGAT

GTTTGGCTCGCTGTCCAAAACAAAAGGGAACGGGAGAAGGGGATGTCTCA

GGACGTCATGTATAAGGTTTACGACATCGATGGAATAGGCGGCGACGAAG

GCATAGAAGACGAAAAGAACATTCCCAACTACAACCAGAACAGCGGATCT

CCTTCGCGAGGGAACGGTGAGAAAAAGCCATTAAAATCCTCCAAACAGAT

ACTACTCAATGCAGGACAAAAGTCTGAATTTGATTCAGGTATGAACAACT

GCCAACTAAGTCACAGCTCAAATAAAGATAGCAAAGTCAGCGGTACCGAC

GCTGATACCACTTATTGCACGTCTTACGGACAAATAATGTCAAGCAGAGA

AAATCACAACCAAATTAATGATATACAACAAAAATTTGCAGCGAATGACA

CTATGAAACCTTCAAGCTCGTTCAGGAAAGACTGGAACCCCGAGCACGGG

AAGACAACGGAAGGGTGGGACGAGTTCTCCAAACGTAACATATCGTCAAT

ACAATGTGAAGGTGAAAGAAGACGGTCCCTCAAATCGGACAAGTCCAAAG

TTAGTAATGGCAAGGCTCACTACGATTTCTTCCCCGTGTTTGACAATAAA

GCTTCTGAAAGTGAGAGCTCCGAAGATAATAATAGAAAAGAACAAGATTT

AAACGAAGACAATTCTAAAATAGTTATAGACTCAATGCAGAAATTAGTTC

TGCATTCGACTGATAACAACATATGTGATAATAACAAAAGTGCTTTCAGC

TCATTAGGCTCATAA

>CodbOR12

ATGAGTGTGGAAGCGAATTTAACGCAGCTTGAAGCTGAGGACAATACGCG

AACTAGCCCACATAGCCCCGTCGTTATTAGAAGGACTGTGGCGCCATCCA

GGAAGTCTGTAGCTACCAGGCTTTGGGATGTTCTACAGCCTCTGGCAAGA

CTATGGGGCCTTATTACTGCTATCGTGATGAGCGGCGCGGGCGCAGAGCT

GGCGGTGCTGGGCTATGACATCGCGCCGCTGGTGCTAGTCGGCGCAGCAT

CGATCTTCATCCTGGAAACCATGTGGGTGGTGGCGCTGTTCGTGGATCTG

CTGTGCCGGCGCGGGGACTACACTCTGTCGCTGCGGTGCTGGGACTTCTT

CCGCTGGTCGTGCGGCCGCGCGCGCGCCCCGTACTACGCGTGTGTGGCCA

CCGCACTGCTCTTTGCTGACTTGACAATACTTACTACCGCTACTGGTGGA

ATGCTGCTGGTGATGGCTGCTCTCCGCGCCGCAGTGCCGTTCTCCCCGTA

CGCAACCCACGGCCCGCACTCGCCGCGGGCGGGTAGCGCGCTACTGAGCC

AACACGACTCGCCCTTGCCAGACGTCTTCTATAACGCCGCTCATACGGGC

GAAGACAGGAGTGAAGAAATGACAGTTTTGGACGTGAAAACTCCGGGGCG

GTCTCGGTCGGTGACGCCGAGGCCTCCTCTACTAGAACTATAA

>CodbIR25a

ATGCACACATGCCTTGCACGTGGACGTTTGTCCTTCCAAATCAGTGGGTT

AGTGCTGGCCAGACCAACATGTCGACGGAACCATACGAGGTCTTCTAAGT

CTAGCCTGACTAAGCGGCGGATCAAAATGGAAATAATAATGCTTTTGTTC

TTTATCGTATTATTTGGTGAAGCGACATGCCAGACGACGCAAAATATTAA

TGTTTTGCTAATAAACGAAGAGAACAATGTCCTAGCAGAGAGGTCATTCG

AAGTAGCTAAAGAGTATGTGAGGCGGAACCCAAGTCTGGGGCTGGCAGTG

GACCCCATAATCGTGGTCGGCAATCGGTCGGATGCGAAAACTTTCTTGGA

GAATGTTTGCAGGAAATACAACGACATGCTTTTAACAAAGAAAACTCCAC

ATGTAGTACTAGACTTCACCATGACCGGCGTAGGTTCTGAGACCATAAAG

TCGTTTACCGCCGCACTAGGGCTGCCCACAATATCGGGGTCTTTCGGCCA

GGCCGGAGATCTGAGGCAGTGGCGCAATATGAACGGGAACCAAACCAGGT

TCTTACTGCAAGTGATGCCGCCAGCCGATATTCTGCCGGAGGCAATAAGG

GCGATTGTCACGAAGTTGGACTCTACTAATGCAGCTATCATTTTCGACGA

GTACTTCGTAATGGACCATAAATACAAATCACTGCTACAGAACATACCCA

CGCGACACGTCATTACTCCGGTCAAAAGCTTCAACAGAGACGAGATCAAA

ACACAGCTCAGAAGTTTGCGAGAATTGGACATAGTCACGTTCTTCGTAGT

TGGCAGTCTGCGGACTATAAAGAATGTTCTGGACGCCGCCGATGAGAACC

AGTATTTCGGGCGGAAGACCTCGTGGTTCGCCCTGTCTTTGGACAAAGGG

GACATAAGCTGCGGCTGCAAGGACGCCACCATAGTCTACATGAGACCAAC

TCCAGACGCCAAAAGCAGAGATCGCCTTGGAAAGATCAAAACAACGTATA

GCATGAACGGCGAGCCGGAGATTACGGCTGCGTTCTACTTCGACCTATCT

CTGAGGACGTTTTTAGCCATCAAGTCGCTGCTGGACTCTGGCAAATGGCC

AAACGACATGCGTTACATTAGTTGCGACGAATACGATGGCCAGAACACTC

CCAATCGGTCCTTGGACCTTAAAACCGCTTTTAAGGAGGTCAAAGAGACA

CCCACGTATGCCCCATTTTACATTCCCGAGGACGATCCCATGAACGGACG

AAGCTACATGGAGTTTACCACGGACCTATCAGCTGTGACAGTCAAGGACG

GGGCCTCTATTGGGAGCAAACCCCTTGGCTCGTGGAAGGCCGGTCTAGCA

AGCCCCCTGGGTTTGACCGACCCTGAAAATATGAGCGATTACTCCGCTCA

GCTCGTCTACCGAGTAGTAACAGTCGAGCAAGATCCATTTATAATCCGAG

ACGACGATGCACCTAAAAAGTTTAAAGGATACTGCATAGATCTCATTGAA

GAAATCCGTCAGATCGTCAAATTCGACTACGAAATCACCCTTTCACCCGA

CGGAAACTTCGGAACTATGGACGAAAATGGCAATTGGAACGGAATAATAA

AGGAGTTGATGGAGAAAAGAGCAGATATAGGTCTGACGTCTCTCTCAGTG

ATGGCAGAAAGGGAAAACGTAGTAGATTTCACGGTCCCCTACTATGATTT

GGTCGGGATAACGATACTGATGAAGTTACCTAGGACTCCGACTTCGCTTT

TCAAGTTCCTCACCGTTTTGGAGAACGATGTGTGGCTGTCAATACTGGCC

GCTTACTTCTTCACTAGCTTTCTGATGTGGGTATTCGACAAATGGAGTCC

GTACAGCTACCAGAACAACCGCGAGAAGTACAAAGAGGATGAGGAGAAAA

GAGAATTTACCTTGAAAGAGTGTCTATGGTTCTGTATGACGTCACTCACA

CCTCAAGGGGGAGGGGAAGCCCCGAAGAATCTCTCGGGAAGGTTGCTGGC

TGCTACGTGGTGGCTGTTTGGCTTCATCATCATAGCGTCGTACACGGCGA

ACCTGGCTGCCTTCCTCACCGTGTCGCGGCTGGACACGCCCATCGAGTCC

CTGGACGACCTCTCCAAGCAGTACAAGATCCAGTACGCGCCGCTCAACGG

CTCCGCTGCGATGACCTACTTCGAGCGTATGGCGCACATTGAGATCAAGT

TCTATGAGATCTGGAAAGAGATGAGTCTCAACGACAGCCTGAGTGACGTC

GAGCGCGCCAAGCTGGCGGTGTGGGACTACCCCGTGTCGGACAAGTACAC

CAAGATGTGGCAGGCCATGCGCGAAGCAGGCCTGCCCAACTCCATCGAGG

AGGCCTTGCAGCGGGTCCGCGACTCCAAGAGCTCCAGTGAAGGTTTTGCG

TGGCTGGGCGACGCGACCGACGTGCGCTACCAGGTGCTGACCAGCTGCGA

CCTGCAGATGGTCGGCGACGAGTTCTCGCGCAAGCCCTACGCCATCGCCG

TGCAGCAGGGGTCGCCGCTCAAGGACCAGTTCAACAATGCAATACTCCAG

CTCCTAAACAAACGCAAACTAGAAAAGCTCAAGGAAGAATGGTGGAACAA

CAACCCTAAAGCCATGAAGTGTGACAAACAGGACGACCAGAGCGATGGGA

TCTCCATCCAGAACATTGGAGGGGTGTTCATAGTCATCTTCATGGGGATA

GGATTGGCCTGCATCACGCTGGGAGTGGAGTACTGGTGGTACAAGTGGAG

GAAACGCCCCACGATTGCTGACGTCACCCAGGTTGAGCCTTCCAAATTTA

CCAAAAACAATGTGGAGGATATCGCTAAACGGTTCGGCGACGGTTTCACT

TTCCGTTCGCGCAACCCGGGACCGGCCGACTTGAAGCCCAAGTTCTAA

> CobdIR75p2

ATGAAATACGTATTGGACAGTCCGATATTCAGTGACAGTGACCTAGTGGT

AGCTGAGAGGATTGGCGAAACATTTCGGATGACTGAACTTCACAAGCCAT

CGACAGTCTCCGGACCCATCCACCACACTTTTCGAGGCCTCTACAACGGA

ACCCTAATAGACGTCAGACCTCACCGTGAGATGTTCAGGCGACGACGAGA

TACAATGGGACACCCGCTTACAATGTCTGTTGTCATTCAAGACAGCAACA

CCACGCAGAACCTTCTACCCATCGAGTCTAGAGCGGAACCACAGCACGAT

TCAATAGCCAAAATTGGTTGGGTAAGCGCTAAAACTGCTTTTGAAATGCT

GAACTCGACACCTCGATACATCTTTAGCCACCGATGGGGATACAAGATCA

ATGGAACCTGGTCTGGGATGATCAATGACCTTGACACGGACAGGGCTGAA

GTTGGATCCAACTGCTACATGACGCTTCAAAGACAAGAGGCCGTGGACTT

TTCTGACATGATGACCCACATCCGTTTGAAGTTCATATTCCGCCAGCCCC

CACTGGCTTTCGTCACCAATATCTTCTCTCTGGCATTTTCAGCAAGAGTC

TGGGTGGTACTGGTGTTGTGCGCTGCCTGTGCTACACTTGCTTTGTTCCT

GAGTACGCGGCATGAAGCCAAATCGGGAACGAGCCGAAGCCAGTTGGACG

GAAGTATAAATGACTCTTTGCTGATGACATTCAGTGCTGTGAGTCAACAA

GGTTGTGCTGTGGAACCAAGGAAGCTTTCAGGTCGCATAGTGGTGTGGGC

ACTGTTTACAGCATATATGGCGCTTTATGCAGCGTACTCAGCCAATATTG

TGGTGCTGCTTCAAGCGCCCTCTGGATCTATAAGGACCCTGTCGCAACTG

GACAACTCCAAAATTACACCTGCGGCGTTAGACGTTGATTACAATCGCCT

TATATTCAATGACAAAAGTTTTATTGCCGACGATCCAGTAAGAATGCGTA

TAGTCAAGAAGATTGAGCCTGAGACGGGCAAACACCATTATTATTCGCTG

GACGAGGGTGTCGAAAAATTAAGACAGGGTATGTTTGCGTTTCACTGCCT

GGTACACACGCTTTACAAAAGAATTGAGGAAACCTTCTTGGATATAGAAA

AGTGTGACCTCGCGCTAGTGGACTTCAGTGGCGAATTCCACGTCTTACGG

CCTCTCAATAGGAATTCACCGTACCTCGAACTCATTAAAGTTGTTTTCCA

ACAAATCCACGAAACCGGCATCCACTCTGTGCACGTCAGCCGTTTTGTAG

TGCCTGTACCGCGCTGCCACGGCGGCGGACCAGCTTTTAGCAGCGTCGGC

ATCGGAGAAACATGGCCCGTCATTTACATGATGCTGTGTGGGTTTGCGAT

GGCTTGTGTCATCGCGGGTTTGGAGATCATTGTGTACAGGATGAACAACC

GGTTGTCGATAGCCAGAGCACAACGATTGAAGAAACAAATTGAACAGGAT

TAA

> CobdIR64a

ATGCACAATGGGAGCTTCGACGGTATGATCGGCACCCTGCAGCGGGGAGA

GGCAGACATCGGGTGCTCTCCCGTTCTTTTCCGACTGGAACGCGCTAAGG

TTGTTGACGCCGCTATGCAGGTCTGGAGATCCAAGCCGTTGTTTATACTG

CGTCACCCGAAGCACCCCGGCGGCTACTCCACCATCTTCTCGCGACCCCT

CACGGCGAGGGTCTGGTGCTGCATCGCGGCTCTCTTGGCGCTCTGCTCTC

TGCTCCTGTGCGCCATGTTCCACTCGAGGATACTGAGCCAAGAAGGCGAC

GTGGACACCTCGTACAGTCTCGCTGTCTTGACCATATGGAGCGCTGTTTG

TCAACAAGGCACCTCAGAAAACCGAAGTTCGTCGTCCATAAAAATAGTGG

TATTCGTCACGTTCCTGTTCACGCTGACGACATACCAGTACTACAACGCC

ACAGTGGTGTCCACGCTGCTCCGGGAGCCGCCGAAGAACATTCGGACTTC

CAGCGACCTGCTGCGGAGCAATCTGAAGCTCGGGGTCCATGATGTCCTTT

ACAATAAAGACTTTTTTAGTCGTACGACCGACCCCGTAGCGATACAAATG

TACAGAACAAAGATAGTCACGTCGACGGAGTATAACTACTTCGCGTTGGA

GACAGGAATGGCGAAAGTAAAGCGCGGAGGTTTCGCGTTCCTGGTGGACA

ATACCATCGCTTACGGCGTCATGCGGGCCACGTTTACTGAGAAGGAGCTC

TGCGAAGCACATGAGGTCTCCCTCTACCCCCCGGAGCATATGGCAGCGAT

TGTGAGGAAGGGATCGCCGTATAAGGAACACTTTACTTATGGGTTACGAC

GAACAATGGAGACGGGCCTGATGCACCGCGTGAAGTCAGTGTGGGACGCG

CCGCGGCCCGCCTGCGTGCGCACTCCCGACTCGAGCCTCATCGCGGTGTC

TCTCGGGGAGTTCTCCATGGCACTGGTGGCCCTTGCGTGCGGCATGTTGG

CCTCGGGCGCCGTGCTGGCTGCCGAACTGGCCCGAAGCTTGGCGCAAGCA

CGAGCACATAAACACCCGACGAGACTGTTGGTAAAAGCAAGAAATGCTCG

GGTAGTGTCCGACTGA

>CodbIR93a

ATGAACGCTTTTAAATGGAAGTCGGCGGTTATACTCCATGATGATACCTT

GAATCGAGACATGGTGTCCAGAGTTGTGAAGTCCCTAACATCTCAGATAG

ATGAGGACAGTGCGCCTTCTGTGTCCGTCTCTGTCTTCAAAATGAAACAT

GAGATCAACGAATATCTGAGAAGGAAGGAGATGTACAGAGTTCTGTCTAA

GCTGCCTGTGAAGTATATAGGTGAAAATTTCATAGCGATAGTGACTACAG

ACGTGATGGCGACCATGGCGGAAACAGCACGAGACCTCACCATGTCTCAC

ACTCAAGCGCAGTGGCTGTACGTTATATCCGATACGAACAAGCAGAACGG

GAACTTGACTGGGTTGATCAACGCTCTTTACGAGGGAGAGAATGTGGCGT

ACATTTACAATATCACAGATGACGATCCAGAATGTCGGAACGGCATAATG

TGCTATTGCCAAGAGATGATGTCTGCGTTCATATCTGCCCTGGATGCGGC

AATCCAGGATGAGTTTGATGTTGCTGCGCAGGTATCTGACGAGGAGTGGG

AGGCCATCAGACCCTCGAAGGTGCAGCGACGGGATATGCTGTTGAGGCAT

ATGCAGCAACACATTGTGCTAAAAAGCTCCTGCGGCAACTGTAGCACATG

GAGAGCATTAGCTGCGGACACATGGGGTGCCACCTATCGCGAACACAGCG

ATAATATCGGCCGGCTGGACGACAAGCCATTTGGGAACAACACTACTGCT

ACCAGTAACATCATTGACCATGTTGAGCTGTTACAGGTGGGATACTGGCG

TCCTATCGACGGAATAAGATTCGATGATGTGCTATTCCCCCACGTGGAAC

ATGGATTTAGAGGAAAAGAGCTGCCAATAATGACTTATCATAACCCACCA

TGGACTATACTGCAGGCTAACGAGTCCGGTGCTATTGTCGGTTACAGCGG

TCTCATGTTCGACATAGTCACCCAACTTGCCAAGAACAAGAACTTCACAA

TCAAAATTTTAGTTCCTGGAAGTGTTAAACAAGATTTCTCGAACAACGAA

ACCACTAGTGATATGATGCACAGTCAGGCAGCTTTGCTGACGCTACAGGC

AGTATCAAGGGGCCAGGTGGCTTTGGCTGCTGCCTCCTTCACTGTACTGG

CTGATCATATGCCAGGAATCAACTACACGACGCCAGTGAGCACGCAGCCT

TACTCCTTCATCATCGCCCGCCCACGAGAGCTGAGTCGAGCGCTACTTTT

CTTGCTTCCATTTACTACTGATACTTGGTTGTGCCTGGGTTTAGCTGTAA

TTCTGATGGGTCCTACGCTGTATGTGATCCACCGACTGAGCCCATACTAT

GAAGCCAAGGAAATTACAAGGCAAGGTGGACTGTCAACCATCCATAACTG

CCTTTGGTACATTTATGGAGCTTTGCTTCAGCAGGGTGGCATGTACCTGC

CCCGAGCTGATAGCGGTCGCCTGGTCGTCGGCACGTGGTGGCTGGTCGTG

CTGGTGGTCGTGACGACCTACTCCGGGAACCTGGTCGCATTCCTCACGTT

CCCCAAGCAGGAGATGCCGGTTACGACCATCGCAGAGCTGGTCGAGAACA

GTGGCACTTATACCTGGTCATTGTACAAGGGTACGTTTCTGGAGATGGAG

TTGAAGAATTCAGACGAGACCAAATATTCGACTCTACTAAAACGAGCTGA

GCTTATGACTGGAGGTGGCGGCATGGAATCCACTATGACGACAGGGAAGA

ACATGTTGAATCGCGTGCGCACTCAAAGACATGCGTTCATAGACTGGAAA

TTACGACTGGCTTATCTGATGCGGGCTGACCACTTGGAGAAAGACGCCTG

CGACTTCTCTCTTAGTGCTGAGGAGTTCATGGACGAACAAGTGGCAATGG

TAGTTCCAGCTGGGAGCCCTTACTTGCCTGTAATCAATAAGGAACTGAAC

CGCATGCACAAAGCGGGGCTCATCTCCAAGTGGTTGTCGGCACATTTGCC

GAAGCGCGACCGGTGTTGGAAGGCCTCTTCCATAGCGCAAGAGGTCGACA

ACCATACTGTGAACCTGAGCGATATGCAGGGGTCGTTCTTCGTCCTGTTT

CTGGGTTTTTTTATTGCATCCAGTGTTTTGTTCATCGAGTGGCTGTGGCA

TCGGAGGAAACGGCGC

> CobdIR62a

ATGGAACAATTCGCATTCAACACTTTGAGTGAGTTAGAAGGATTTGAAGT

TGATTATACTTTCGCTGATTTGGGAGAAAAGATGTCTAAGGTTGACGAGG

ATAACATAGCTCAAGGGGTTCTACGTCGCATTCAGACGAAAGAAATGGAC

GCAGTGTTCGGTTTTATGATGTTGACTAGTGCACGTGGCGCGGCTTTCGA

CTACCTGTACGGGCACCTCGCCTTTACCGACGAGTTCACACACTTTGTAC

AAACAGCCGGGCCGGTCAGTGCATGGAAAAACTTGTACTTGGTATTCACA

AAGACAGTTTGGTTGATGATTTTCCTATCCTTTGTCTCATACGCTATATT

GATGATAATGCTCATACGTGACCGAGATCGAGTGTCCATCATTTTAAAGA

TGGTGGATGCGTTGGTTCTTCATGGGCCTACTATTCAATCGAGATTTCTG

GTGAAAGTAATCTTTCTTTTGTGGATTTGGTTCGCCTATTTGATTAATTC

CATCTATCAGTGCAGTCTGTTTGGCCTGACCACGCATCCATATAAAGAAT

ACCAAGTGAAGGGTGTGGAAGATTTAAAAGTGTATAACTATGAGCCATGC

GTTAGCGACGCTTTTAATGAGTTCATGAAGGTCGAGGCAGGGGTTACTTT

TGAGCAACGCGACGAGTGTAAGCGATTCTTACAGGCCAATTTATACGTGA

GCAATCATCATGGAGTTTATACTCTCTCAACGCGTTTGCTTTTTTTTTAT

TCACAGTATCTTTATTATGACGCGAATGGAAAAAGCAAACTTTGGCCCTT

TGAGAAAGCAATTAATAAAATAAGTTATGCAATTTATTTGTATAAAGGGT

TCCCGGTGTATCAGAAATTGCAACTCCATACTCTAAGATTGCGAGAGAAT

GGTCTGATGCTTTATCATTTTGAAAGATTGTATGACGAGCAGAAAAAGAA

ATATCCCGATTTACAAGGAAGC

> CobdIR68a

ATGTTGGGGAAGTATTCCAAATTGTTATTTTTGATTTCTCTCATTGTGAC

AATCTTGGTTGCCGCTACAGCTATCTGGACTGGACCGTACATCGTCAAGA

AACAAATTCAAAAGAGCGTTCAGCTAGACAGCTCATCTCAGATGTTCGAC

AAGTGGAAGAAACTTCCTATTCCCCTGAACTTCAAGATACGGGTCTTCAA

TGTAACGAACCCTGATGAAGTTAATAGTGGCGAAAAACCTAAACTGGCTG

AAATTGGACCCTATGTTTATAAAGAGTACCGGGAGAAGAGAATCCTCGAC

TACGGCGAAAATGACACGATAAGGTATATGCTGAAGAAGAGTTACTTCTT

CGATCAGGAGCAATCTGGCTCCTTGACAGAAGATGATGAAGTCACTGTAA

TCAACTTCTCTTACTTGGCGGCTATACTAGCAGTGGAGGAGATCATGCCC

AGTTTCGTGGGGACAGTGAACCAGGCGTTAGAACAGTTCTTCGAAGATCT

GCGGGACCCATTCCTGAGGGTCAAGGTTCGAGATCTGTTTTTTGATGGGG

TGTTCCTCAACTGCAGTGGAGATCATTCTGCTTTGGGCCTCGTTTGCGGT

AAGATTAAGTCCGACACGCCACAGACCATGCGACCAGCGGAAGATGGAAC

GGGGTATTTCTTCTCCATGTTTTCTCATCTAAATAGAACAGAGTCAGGCC

CATACGAAGTCGTCCGCGGGAGGGACAACCTTTACGAGCTTGGTCGCGTA

GTATCCTACAAGAACAAAACCGTGTTGAACAACTGGAGGGACCCATATTG

TGGACAAATTAACGGAACAGATTCGTCCATCTTTCCGCCTATAGATCAGA

GTAACGTGCCTAGGAGATTGTATAGCTTTGAGCCAGATATATGCAGGTCT

CTTTACGTGGAATACGTTGAGGAGAGGTCCATTTTCAACCTGTCAGCTTA

TTACTACGAGATACCGGAATCAGCTTTAGCGGCAAAGAGCGCCAACAAGA

ATAACAAATGCTTCTGTAGAAGAAACTGGAGCGCAAATCACGACGGTTGC

CTAATCATGGGTGTCCTCAACCTGGCTCCGTGTCAAGGAGCTCCCGCCAT

CGCCTCCTTCCCCCACTTCTACATGGCTTCCGAAGAACTCCTGGAATACT

TCAATGGAGGAATCGAACCCGAGAGAGAAAAACACGCTACTTATTTGTAC

TTAGATCCGCTTACAGGAGTTGTTTTGAGAGGTGCAAAAAGGTTACAGTT

CAACATTGAATTGCGAAACATTAAAGGAATTCCACAGTTGGAGAATGTTT

CCACTGGATTATTTCCACTGCTTTGGATTGAAGAGGGAGCAGAAATTCCT

GTTTCAATCGCAGAAGAGCTCCAAGCCTCCCACAAGTTAGTGAAGAACGT

CAAATTGCTGCCCTGGATCTTTTTTGGAATTTTTGCACTTGCTACGGTTG

CAAGTGCGTGTGCTGTACTCAGAATGTTACCTTTCTTTAGAAAACACACG

AATTCTGTCAGCTTTATACTAAATTCTGACGCTAATAAAGTGAGAACA

> CobdIR76b

ATGGCCACCGGTATTGAACTCATCATCTCAGCCTTTTGCAATGCCACTTT

CTGTGAGCCTGTATACGATAATCCTTTAAAAGAGGATGCTGAGGAACTGC

GGATCGAGCAACTGATTCACGATCTGAATGGGAAACATCTAAAAGTGGCC

ACTTACACCAACTACCCTCTAAGCTGGGTAAAAAGGAGCGAAAATGGGAC

ATCAACGGGAAAAGGCGTGGCCTTCATCATCATGGACGTGCTGCAGGAGA

AGTTCAACTTCACCTACGATATCGTTACACCGGAGATGAACTTTGTCATC

GGGGGTAGCAAGCCACAAGACTCGTTGGTGGGGTTACTGAATAGTAGCGC

CGTGGATATGGCGGCTGCGTTTATACCGCGCCTTCCGAAGTTGTACCTTA

AGGTCGACTTCTCTGATACACTCGACGAAGGCACGTGGATGATGATGCTG

AAGCGGCCCAAGGTGTCTGCAGCAGGGTCTGGGCTATTGGCGCCGTTCGA

TGAATATGTGTGGTATCTAATCTTAGCAGCAGTGCTTTCCTACGGACCTA

TCATCACACTCTTGACGAAACTGCGCTTAAAATTCTCAAGAGACAAGAAG

GAGATGCACATACCACTGTCACCCAGTTTCTGGTTTGTCTATGGAGCGTT

CATCAAGCAAGGGACTTCTCTTGCTCCCGAAGCCAACACAACGAGAATAC

TGTTCGCAACATGGTGGCTGTTCATAATCCTGCTATCATCCTTCTACACG

GCGAATCTGACCGCTTTCCTGACCCTGTCCAAATTCACGCTGGCGATCGA

GGAGCCTCGCGACCTGCTGAAGAAGAACTATCGCTGGATAGCAGACGAGG

GCGGGCCGGTCGAATACATCATTAGGGATGAAGATGAAGATCTAAACTTC

CTCTACCAGATGGTACAGAACGGTCGCGCAGAGTTCCGCTCATACCTTCG

AGACAGGGACTTCCTTCCCCTGGTGGCAGGGGGAGCGGTGCTGATCAAGG

AGTCCACCGGAGTAGACCACTTGATGTACGGAGAATACCTCAGGAAAGCG

AAAGAGGGCATCGCGGAAGAATCTAGGTGTACATACGTCATCGCACCAAA

TAGTTTTGTAGTGGTGCCGAGGGCGTTTGCGTATCCCAGAGGCAGCTCTC

TAAAGCCCCTGTTTGATCCTATCTTCACCGACCTCCTGCAAGCGGGCATC

ATCACGTTCCTGGAGCACAAGAACCTGCCCAGCACTAAGATCTGCCCGCT

GGACCTGCAGTCCAAGGATAGGAAGCTGAGCAACACTGACCTGATGATGA

CCTATGTGATCATGCTGATTGGGCTCGCTGCTGCCAGCGCAGTTTTTGGG

ATGGAGATTCTATTGAGGCGTTACATTCGCGTCAAGGTGAGAAACAACGA

TGGTGTTAAGAAAAAGAAATCAAGACGAATTCGGTTCAAAGACAAAGAAG

CAGACGTCCCACCACCGCCTTACGACTCGCTTTTTGGTCGAAACCCCCAA

TACAAAGTCAGCGGGAACGCCAGAACGAAGATAATAAACGGGAGAGAGTA

TTACGTAGTACATACAGTTAGCGGGGATTGTAGGTTGATACCGGTTAGGA

CACCGTCTGCATTTTTGTATCGGTAA

>CodbGR1

ATGTACAAGTCATTTGTACTGTTTTGTGTGGTGCTGTGTGTACTTGTTGA

GGCCCGCCCCAAATGGCAGTTCCTGGCACCGAAGCCCGGCTACGTGCCGG

TCTACATCAGGCCAGGAGACACTCCCCTCGAAGAGATCAACCCTGACTTA

GCAGAGGCCTTCCACGCGTTCCCCGCGGGCCGCAGCGCCGGCAAGGAGCT

CGAGGCCTCCCCGGAGGCCCCCGAGGCCCCCGACGCAAAGGCCGACCTAG

AGGAACCCGGGGACTCAGCTGTCGACCGCCGCCCGTACGAGAAGAAATTT

GTAGAGAAGAAAACCAAAAAGCCAACGGAATTTACTAAGCCAGAAGAACG

TAGATAG

>CodbGR64

ATGTTTTATGTGGTGATAGTGTGCAATTTTATTTTTCTGCTACAACAGTT

CCCGTCAACGTTCACAAGCGAGCCTGGCAACTCTATCCGGTCCCTCCACC

TGGGATCACAGCAGCTGCGGTACGAGAATGCCTGCGACAACATCTGCAAT

GACCTTCCTCTCGATGACAGGGTGCACGTCTGCATGATATCCAGCCGTTA

CAAGAAGGGTTTACAGTTTTTTGTCAACATGTGTCATGCAAGACGGTCGA

TCTGTAAGGATAATTTAGTGCTACACATGGTCCACCCAGAAAGATGCCTA

AGAAGCAAACAGTTCTTTATGTCAATGAATTAA

>Codb43a

ATGTCTACTCTCCTGCATCTCATAGTTACGCCTTACTTCTTGATTATGGA

GATCATTGTGTCCGTCAACAGAATACACTTCTTGGTGCTGCAGTTCCTGT

GGTGCGCCACGCACGTGCTGCGACTGTTTGTGGTGGTTGAGCCCTGCCAT

TATACCATCGCAGAGGGCAAAAGAACAGAAGGCCTAGTCTGCCGGCTGAT

GATGTCAGCGCCATCTACCGGCGTACTC

>CodbGR2

ATGGTTTTCTCCCGAAGCTCATTTATATGTTTGCCCCATCTCATGGAGCG

ATCTAACCAAAGCCCCAAATATTTTGCTGATTCAACTACACTGATTTTAA

TTTTGTTAATTTTGATTGGAACAATCTCTTTTTTATAA

>CodbGR3

ATGTACAAAACCGTCGAGTACTCCCCGCCGCGCTTCTCCGTCTACGACAT

GTGGCAAATGGACGCGCGCATTTTAATCAAGATATTCAACATACTCAGTA

CCGTCTTCGTAGCTCTACTGCAGTTCGCTTTTGTTTGA

>CodbGR4

ATGCATACTTTAAGTAGGTTATTATTAGATCTTGGACTAGAGATATCTCC

GTCAAAAACAAAAGTATGCATATTTAAAAAAGGATTCAGAAGGGATCATA

TCCGAATAAGCATTAATAATTGTAATTTACAAGTAGTTGATAATGTGAAG

TATTTAGGTCTGTGGTTGGATCGAAATTTAAGATGGAATAAACATATTAA

CGAAACTAGAATAAATACTTTGAAATTTATGAACGTTTTAAAAGTTTTGT

CTGGACCGGGCTGGGGAGTGCACCCTGTACACATGCGACGGTTATACATA

GCTATTGCGCGCAGTAGATTGGATTACGCCAGTATTTTTTATAGTAATAG

CTGCAAAAGTAATTTGAATAAACTCGATAAAATTCAAAATCAATGCTTAC

GTATAATCGGCGGTTTTATTAAAACAACCCCTATTCATGTAATGGAATGT

GAATTATTTCTTCCACCTCTGCATATCAGGCGTTACTATCTAGCGGTCAA

ATTTTGGTTGAAATGCAGATCATTTTTGAATAGTGATTTTATCGACGTAT

TAGATAAACTATATTATTTAAGACAGTGCCCCTATTGGCGTCGTAAGCAG

TTACCGTTGACTGTCAAAGTTCACGATCATTTTAAGAATGTACCGTTACA

CCGAAGTAGACAATTAGAAATGTTTTCTTTGAATACATGGGTAAGCAATA

TAAATTTAGCAAATACAATTAGCCCCCATATAGATACTGTACAAAGGGGT

AAACATAAATATAATGAATTACAATTAAGGAATTTATTTAATCAGTTTAT

ATTTGATAAATACTCGAAATCTTATTTTGTATATACTGATGGCTCCAAAT

GTGGTACTGACATTGGTGCGGCATTATTTGATCCGCAAGAAAATATATAC

TATAAATTTAAAATTACATCAGATATTTCTATACTGTATGCAGAATTAAA

ATAA

>CodbGR5

ATGGAGAGTGAATGTTGTGTTCCTCCTCTATCCTTGAGAAGGCACTATCT

GGCCTACAAATACTGTTTAAAATCATTATCGTTATCAAATAATGTAACTA

TCGAATTATTACGTAATCTTGAAGTTATGTATGATGTACAAAATAATCAT

AAAATTCCCCTATTAATAAAAGTGTATAATGATTGTAAAAGTTTAAAAAT

ACGTTCGACTAACCCGTTAGAAATGTTTAGGTTCAATACGTGTATAACTA

ACATAAATGTACATGAGATGATCAAAACGTCCCTAGACACAATTATAGGT

TCTAAAAAATCATACAACCCCCATATTCTTAGACTACATACACTAAAAGA

AATTTATCAAAAATATTTAAACTACTACAAAATTTATACAGATGGGTCAA

AAAATGATGGTATTGGTGCCGCATTTTTTGACCCAATTAGCGACACGAGT

TATTGTTATAAAATAACATCAGATATTTGCATTATGTCGGCGGAGTTATT

TGCAATATCAGAGGCTATATCTTATATATTACAATTGGGATCTGAAGGCA

AATTTGTAATTTTTACTGACAGTAAAAGTTCACTTCAGCAGATAGCTCGA

TGTGCCTCTGGTGAAAGAGGATTTTCGCTAGCTTACTCAATTATTGATAA

ATTTTTGGAATGTCAACTTAAGGGTGTAAATCTGATACTGCAATGGATTC

CGTCACATGTCGGAGTCTCCGGAAATGAAAAAGTAGATTCATTGGCAAAA

TTGTCTATTAATATCGGCTCTGAAATATACATACTTCCAAATTTTACAGA

GTATTTGTGTAAGTTTAAAAGAAAATGTTATTTGTTTTGGAAAGAATATT

TTGATATCAGATCTAAA

>CodbGR6

ATGATATACAACTTGGGATGTGTCGGTAGAAAGTTCGTTCTTAATTTATA

TAATTTAATCTTACAATCAGGTCAAATTCCACAACAATGGCGTGAAATAG

AAATTGTGCCTATTCCCAAGTCAAATGGAAGTACCAACGCCACAAGTAAA

ATTAGACCTATCTCCTTAATAAGCTGTCTGTGTAAAATTTTCCATTTGAT

GCTATGTAAACGCCTTGAATGGTTTATCGAGAAAAACAAAATATTGTCAC

CCTACACTAGCGGATTCCGAAGAGCTCAATCCTGTTTAGACAATTTAGTC

AGATTAGTTACATATATCCAGTTAGGTTTTAAATCAAATATCAGCACATT

AGCATGTTTTATAGATATTGAAAATGCCTACAATGAAGTTTTAATAGAAA

AGGTAGTTTTTGTTCTCGATTCCCTTTATGTCGGGTCTAAAGTTTGTAAT

TATTTATGGCATTTTCTAAGTGAAAGACGTCTAAAGATTAAAAACGATAA

TAATCATTCTAGATGGTCATGTAAAGGTTTGGCACAAGGCGACCCCTTGT

CACCCCTTTTGTTTAATATTGTTACACATGAAATCTGTCGCCAAACACAA

AATATTATAGTAATATCTCAATATGCGGACGATTTTGTTCTTTACGTCAG

ATCTAATAACTTACATGATAGTGTGATCAAAATACAGCAATCTTTGGACG

TGATGACTTACTTGTTAGATGAGATCGGATTAACTATATCTTGTAATAAA

TAA

>CodbGR7

ATGGTGGACAGCAAGATCGGCCCGCTCGTCCTGCTTTCCAACTTGAACAA

CTTCTACTTCATTTGCTTGCAACTGTTTTTGGGTATTAACAAGAACGCAC

AAGGAGGCGGCTTCCTACAGCGTCTCTACTATTTCTTCTCCCTCGGCTGG

CTGCTGTTCAGGGCCAGCGCCGTGGTGCTCGCAGCCGCTGAGGTCCACCA

CCAGTCTAAGCAGGCGCTACCCTTTCTGTTTTCATGTCCTGCCTCTTGCT

ACAATATTGAGATAAAAAGATTGAAAAATCAACTGGCTCACGAGTGTGTA

GCTCTAAGTGGAATGGGCTTTTTTTATTTGGATCGGCAATTGTTATTAGA

GGTGGCTGCAGCTATACTGAAATATGAGTTGGTGCTGATCCAATATGACC

AAACGTAA

>CodbGR8

CTGTATTGTAACAGCCCGTATTTATGGACTGAAAATCTTCAAGAAGCTTA

TGACACTTTTGTAGATTATATAAATATTGCTGCTGAACTATTCATACCAA

TTATAAAAATCAATCAAGACCCTACTAGTTCGTTTAGCCCTAAACCTTAT

TGGAATGAAGACATCTCGAAGGCAGTAGCTGAGCGACGCTTAGCCTTAGC

GATATTTCAAAAGAACCCTACCCCCCGTAATTCAGAAGTTTTAAAAGACA

AAATTGGAATTTCACAGTGTCTAATACGTAAAGCAAAATCTAAAAGCTGG

CAAGATTTTTGTTCCTCATTTGATCAATCGGTGTCTCTGAGTGAGGTTTG

GCGAAGGTTACGATGGGTCAAGGGAAAAAAGTCCTCATCGTTTTATGTAG

ATAGTTATAAAGCGAACGAATTATTACATAGCTTATCACCTGATTTTGTT

ACACCAGACCAACCTAGTTTTAAATCATCAAACCCTTCATTAGAAATAGA

TATCTCACTTTCAGAGTTAGAATGTCACTTAACCAAGAAAAATACCTCCC

CAGGGGCCGATGGCATAAACTACTCAATGATAAGATATTTACCTAATAAC

GGGAAAGTATTATTAATAAAACTCTATAATAAAATT
