# Supplementary material for: The whole body transcriptome of Coleophora obducta reveals important olfactory proteins
Source: PeerJ. 2020 Apr 10;8:e8902. doi: 10.7717/peerj.8902 (PMC7153557; doi:10.7717/peerj.8902)
Supplement: Supplemental Information 2 [file peerj-08-8902-s002.docx]

**The whole body transcriptome of *Coleophora obducta* reveals important olfactory proteins**

Dongbai Wang^2^, Jing Tao^3^, Pengfei Lu^3^, Youqing Luo^3^, Ping Hu^1,2^

^1^ Guangxi University, Nanning, Guangxi, China

^2^ Xingan Vocational and Technical College, Xinganmeng, Inner mongolia, China

^3^ Beijing Key Laboratory for Forest Pest Control, Beijing Forestry University, Beijing, China

**Supplementary file 2**

**The protein names and gene accession number were used in phylogenetic trees**

| **CSP** | | **CSP** | |
| --- | --- | --- | --- |
| **Accession NO.** | **Name** | **Accession NO.** | **Name** |
| EFA07564.2 | TcasCSP14 | AOG12897.1 | EhipCSP6 |
| KYB26756.1 | TcasCSP13 | AOG12896.1 | EhipCSP7 |
| EFA07640.1 | TcasCSP8 | AOG12895.1 | EhipCSP8 |
| EFA07577.1 | TcasCSP19 | AOG12894.1 | EhipCSP9 |
| EFA07570.1 | TcasCSP18 | AOG12893.1 | EhipCSP10 |
| EFA07566.1 | TcasCSP12 | AOG12892.1 | EhipCSP11 |
| EFA07563.1 | TcasCSP11 | AOG12890.1 | EhipCSP13 |
| EFA07552.1 | TcasCSP10 | AOG12888.1 | EhipCSP14 |
| EFA07424.1 | TcasCSP7 | AOG12887.1 | EhipCSP15 |
| EFA07423.1 | TcasCSP1 | AOG12885.1 | EhipCSP17 |
| EFA07422.1 | TcasCSP9 | BAV56823.1 | OfurCSP 19 |
| EFA07421.1 | TcasCSP6 | BAV56820.1 | OfurCSP 16 |
| EFA07420.1 | TcasCSP2 | BAV56818.1 | OfurCSP 14 |
| EFA07419.1 | TcasCSP5 | BAV56817.1 | OfurCSP 13 |
| EFA07418.1 | TcasCSP4 | BAV56816.1 | OfurCSP 12 |
| EFA07417.1 | TcasCSP3 | BAV56815.1 | OfurCSP 11 |
| EFA01297.1 | TcasCSP20 | BAV56814.1 | OfurCSP 10 |
| AAF56814.3 | DmelCSP A98a | BAV56812.1 | OfurCSP 8 |
| AAS64792.2 | DmelCSP B42c | BAV56811.1 | OfurCSP 7 |
| AAF53928.2 | DmelCSP B38c | BAV56810.1 | OfurCSP 6 |
| AAZ66059.1 | DmelCSP B74a | BAV56809.1 | OfurCSP 5 |
| AAF49268.2 | DmelCSPA75a | BAV56808.1 | OfurCSP 4 |
| AAZ52819.1 | DmelCSP A46a | BAV56806.1 | OfurCSP 2 |
| AAZ52804.1 | DmelCSP A56a | BAV56805.1 | OfurCSP 1 |
| AAZ52549.1 | DmelCSP A86a | AII01043.1 | DkikCSP2 |
| AAN13876.2 | DmelCSP B93B | AII01040.1 | DkikCSP4 |
| AAF55894.2 | DmelCSP B93a | AII01039.1 | DkikCSP5 |
| AAZ52518.1 | DmelCSP A84a | AII01038.1 | DkikCSP6 |
| AAF46258.2 | DmelCSPA7a | AII01037.1 | DkikCSP7 |
| AAX52702.1 | DmelCSP B53B | AII01036.1 | DkikCSP8 |
| AAX52701.1 | DmelCSP B53a | AII01035.1 | DkikCSP9 |
| AAM70837.2 | DmelCSP B42a | AII01034.1 | DkikCSP10 |
| AAS64791.1 | DmelCSP B42B | AII01033.1 | DkikCSP11 |
| AAS64728.1 | DmelCSP B38a | AII01032.1 | DkikCSP12 |
| AAS64727.1 | DmelCSP B38B | AII01031.1 | DkikCSP13 |
| AAO41170.1 | DmelCSP A29a | AII01030.1 | DkikCSP14 |
| vAAF54934.1 | DmelCSP A87a | AII01029.1 | DkikCSP15 |
| AOG12902.1 | EhipCSP1 | AII01028.1 | DkikCSP16 |
| AOG12901.1 | EhipCSP2 |  |  |
| AOG12900.1 | EhipCSP3 |  |  |
| AOG12899.1 | EhipCSP4 |  |  |
| AOG12898.1 | EhipCSP5 |  |  |

| **CXE** | | | | | | **CXE** | | | | | | | | | **CXE** | | | | | | |  |  |  |  |  |
| --- | --- | --- | --- | --- | --- | --- | --- | --- | --- | --- | --- | --- | --- | --- | --- | --- | --- | --- | --- | --- | --- | --- | --- | --- | --- | --- |
| **Accession NO.** | | **Name** | | | | **Accession NO.** | | | | | **Name** | | | | **Accession NO.** | | | | **Name** | | |  |  |  |  |  |
| AF448479 | | TmolJHE | | | | KX015868 | | | | | EoblCXE26 | | | | MK864068 | | | | PintCXE8 | | |  |  |  |  |  |
| FJ652463 | | SlittCXE20 | | | | KX015869 | | | | | EoblCXE27 | | | | MK864067 | | | | PintCXE7 | | |  |  |  |  |  |
| FJ652462 | | SlittCXE19 | | | | KX015870 | | | | | EoblCXE28 | | | | MK864066 | | | | PintCXE6 | | |  |  |  |  |  |
| FJ652461 | | SlittCXE18 | | | | KX015871 | | | | | EoblCXE29 | | | | MK864065 | | | | PintCXE5 | | |  |  |  |  |  |
| FJ652460 | | SlittCXE17 | | | | KX015872 | | | | | EoblCXE30 | | | | MK864064 | | | | PintCXE4 | | |  |  |  |  |  |
| FJ652459 | | SlittCXE16 | | | | KX015873 | | | | | EoblCXE31 | | | | MK864063 | | | | PintCXE3 | | |  |  |  |  |  |
| FJ652458 | | SlittCXE15 | | | | KX015874 | | | | | EoblCXE32 | | | | MK864062 | | | | PintCXE2 | | |  |  |  |  |  |
| FJ652457 | | SlittCXE14 | | | | KX015875 | | | | | EoblCXE33 | | | | MK864061 | | | | PintCXE1 | | |  |  |  |  |  |
| FJ652456 | | SlittCXE13 | | | | KX015876 | | | | | EoblCXE34 | | | | KX015866 | | | | EoblCXE24 | | |  |  |  |  |  |
| FJ652455 | | SlittCXE12 | | | | KX015877 | | | | | EoblCXE35 | | | | KX015867 | | | | EoblCXE25 | | |  |  |  |  |  |
| FJ652454 | | SlittCXE11 | | | | NM_079544 | | | | | CG2505aE2 | | | | MK864070 | | | | PintCXE10 | | |  |  |  |  |  |
| FJ652453 | | SlittCXE10 | | | | NM_079545 | | | | | CG1031aE1 | | | | MK864069 | | | | PintCXE9 | | |  |  |  |  |  |
| FJ652452 | | SlittCXE9 | | | | NM_137834 | | | | | CG6018aE1-0 | | | | KX015862 | | | | EoblCXE20 | | |  |  |  |  |  |
| FJ652451 | | SlittCXE8 | | | | NM_001260036 | | | | | CG1089aE5 | | | | KX015863 | | | | EoblCXE21 | | |  |  |  |  |  |
| FJ652450 | | SlittCXE7 | | | | NM_079537 | | | | | CG1112aE7 | | | | KX015864 | | | | EoblCXE22 | | |  |  |  |  |  |
| FJ652449 | | SlittCXE6 | | | | NM_079535 | | | | | CG1121aE8 | | | | KX015865 | | | | EoblCXE23 | | |  |  |  |  |  |
| FJ652448 | | SlittCXE5 | | | | NM_169187 | | | | | CG1128aE9 | | | | MK864073 | | | | PintCXE13 | | |  |  |  |  |  |
| FJ652447 | | SlittCXE4 | | | | NM_079543 | | | | | CG1257aE3 | | | | MK864072 | | | | PintCXE12 | | |  |  |  |  |  |
| FJ652446 | | SlittCXE3 | | | | BT044260 | | | | | CG1108aE6 | | | | MK864079 | | | | PintCXE19 | | |  |  |  |  |  |
| FJ652445 | | SlittCXE2 | | | | NM_079542 | | | | | CG1082aE4 | | | | MK864078 | | | | PintCXE18 | | |  |  |  |  |  |
| DQ680828 | | SlittEst | | | | NM_079034 | | | | | CG8425JHE | | | | MK864077 | | | | PintCXE17 | | |  |  |  |  |  |
| AB259898 | | PhilJHE | | | | NM_137241 | | | | | CG8424 | | | | MK864076 | | | | PintCXE16 | | |  |  |  |  |  |
| AY866482 | | PjapPDE | | | | AF153367 | | | | | CfumJHE | | | | MK864075 | | | | PintCXE15 | | |  |  |  |  |  |
| AF302777 | | NlugCXE | | | | NM_001043562 | | | | | BmorJHE | | | | MK864074 | | | | PintCXE14 | | |  |  |  |  |  |
| EU380769 | | NlugJHE | | | | XM_393293 | | | | | GB16889 | | | | KX015857 | | | | EoblCXE15 | | |  |  |  |  |  |
| AF327882 | | MsexJHE | | | | NM_001126244 | | | | | GB10820 | | | | KX015858 | | | | EoblCXE16 | | |  |  |  |  |  |
| J04955 | | HvirJHE | | | | XM_391943 | | | | | GB11403 | | | | KX015859 | | | | EoblCXE17 | | |  |  |  |  |  |
| EF558769 | | GassJHE | | | | NM_001145739 | | | | | GB13939 | | | | KX015860 | | | | EoblCXE18 | | |  |  |  |  |  |
| KX015843 | | EoblCXE1 | | | | NM_001145736 | | | | | GB18290 | | | | KX015861 | | | | EoblCXE19 | | |  |  |  |  |  |
| KX015844 | | EoblCXE2 | | | | XM_396706 | | | | | GB12309 | | | | MK864071 | | | | PintCXE11 | | |  |  |  |  |  |
| KX015845 | | EoblCXE3 | | | | AY866480 | | | | | ApolPDE | | | |  | | | |  | | |  |  |  |  |  |
| KX015846 | | EoblCXE4 | | | | AY091503 | | | | | ApolODE | | | |  | | | |  | | |  |  |  |  |  |
| KX015847 | | EoblCXE5 | | | | AY091504 | | | | | AploIE | | | |  | | | |  | | |  |  |  |  |  |
| KX015848 | | EoblCXE6 | | | | MK864088 | | | | | PintCXE28 | | | |  | | | |  | | |  |  |  |  |  |
| KX015849 | | EoblCXE7 | | | | MK864087 | | | | | PintCXE27 | | | |  | | | |  | | |  |  |  |  |  |
| KX015850 | | EoblCXE8 | | | | MK864086 | | | | | PintCXE26 | | | |  | | | |  | | |  |  |  |  |  |
| KX015851 | | EoblCXE9 | | | | MK864085 | | | | | PintCXE25 | | | |  | | | |  | | |  |  |  |  |  |
| KX015852 | | EoblCXE10 | | | | MK864084 | | | | | PintCXE24 | | | |  | | | |  | | |  |  |  |  |  |
| KX015853 | | EoblCXE11 | | | | MK864083 | | | | | PintCXE23 | | | |  | | | |  | | |  |  |  |  |  |
| KX015854 | | EoblCXE12 | | | | MK864082 | | | | | PintCXE22 | | | |  | | | |  | | |  |  |  |  |  |
| KX015855 | | EoblCXE13 | | | | MK864081 | | | | | PintCXE21 | | | |  | | | |  | | |  |  |  |  |  |
| KX015856 | | EoblCXE14 | | | | MK864080 | | | | | PintCXE20 | | | |  | | | |  | | |  |  |  |  |  |
| **OR** | | | | | **OR** | | | | | | | | | **OR** | | | | | | | **OR** | | | | |  |
| **Accession NO.** | **Name** | | | | **Accession NO.** | | | | | **Name** | | | | **Accession NO.** | | | | **Name** | | | **Accession NO.** | | | **Name** | |  |
| AII01110.1 | DkikOR1 | | | | AJO62235.1 | | | | | TmolOR16 | | | | CUQ99405.1 | | | | MsexOR23 | | | AUW64516.1 | | | LpraOrco | |  |
| AII01109.1 | DkikOR2 | | | | AJO62234.1 | | | | | TmolOR15 | | | | CUQ99404.1 | | | | MsexOR22 | | | AUW64515.1 | | | AsutOrco | |  |
| AII01108.1 | DkikOR3 | | | | AJO62231.1 | | | | | TmolOR12 | | | | CUQ99403.1 | | | | MsexOR21 | | | AUW64514.1 | | | AfasOrco | |  |
| AII01107.1 | DkikOR4 | | | | AJO62230.1 | | | | | TmolOR11 | | | | CUQ99402.1 | | | | MsexOR20 | | | AKO69815.1 | | | CchlOrco | |  |
| AII01106.1 | DkikOR5 | | | | AJO62229.1 | | | | | TmolOR10 | | | | CUQ99401.1 | | | | MsexOR18 | | | AKC58535.1 | | | AcorOrco | |  |
| AII01105.1 | DkikOR6 | | | | AJO62228.1 | | | | | TmolOR9 | | | | CUQ99400.1 | | | | MsexOR17 | | | AHL20247.1 | | | AalbOrco | |  |
| AII01104.1 | DkikOR7 | | | | AJO62227.1 | | | | | TmolOR8 | | | | CUQ99399.1 | | | | MsexOR16 | | | AGF29886.1 | | | CpunOrco | |  |
| AII01103.1 | DkikOR8 | | | | AJO62226.1 | | | | | TmolOR7 | | | | CUQ99398.1 | | | | MsexOR15 | | | AEX28371.1 | | | SgreOrco | |  |
| AII01102.1 | DkikOR9 | | | | AJO62225.1 | | | | | TmolOR6 | | | | CUQ99397.1 | | | | MsexOR13 | | | AEX28370.1 | | | LmigOrco | |  |
| AII01101.1 | DkikOR10 | | | | AJO62224.1 | | | | | TmolOR5 | | | | CUQ99396.1 | | | | MsexOR12 | | | AFX73448.1 | | | LlinOrco | |  |
| AII01100.1 | DkikOR11 | | | | AJO62223.1 | | | | | TmolOR4 | | | | CUQ99395.1 | | | | MsexOR11 | | | AFX73447.1 | | | LhesOrco | |  |
| AII01099.1 | DkikOR12 | | | | AJO62222.1 | | | | | TmolOR3 | | | | CUQ99394.1 | | | | MsexOR10 | | | AWO14314.1 | | | LstrOrco | |  |
| AII01098.1 | DkikOR13 | | | | AJO62221.1 | | | | | TmolOR2 | | | | CUQ99393.1 | | | | MsexOR9 | | | AVO63533.1 | | | YsigOrco | |  |
| AII01097.1 | DkikOR14 | | | | AJO62220.1 | | | | | TmolOR1 | | | | CUQ99392.1 | | | | MsexOR8 | | | APZ81509.1 | | | AlinOrco | |  |
| AII01096.1 | DkikOR15 | | | | ABY55454.1 | | | | | DmauOR33b | | | | CUQ99391.1 | | | | MsexOR7 | | | AHC72290.1 | | | AlucOrco | |  |
| AII01095.1 | DkikOR16 | | | | ADD14628.1 | | | | | DmauOR85a | | | | CUQ99390.1 | | | | MsexOR6 | | | AJO62219.1 | | | TmolOrco | |  |
| AII01094.1 | DkikOR17 | | | | ADD14627.1 | | | | | DmauOR59c | | | | CUQ99389.1 | | | | MsexOR5 | | | AVN97813.1 | | | AchiOrco | |  |
| AII01093.1 | DkikOR18 | | | | ADD14626.1 | | | | | DmauOR59b | | | | CUQ99388.1 | | | | MsexOR4 | | |  | | |  | |  |
| AII01092.1 | DkikOR19 | | | | ADD14625.1 | | | | | DmauOR43b | | | | CUQ99387.1 | | | | MsexOR1 | | |  | | |  | |  |
| AII01091.1 | DkikOR20 | | | | ADD14624.1 | | | | | DmauOR42b | | | | ACM18059.1 | | | | MsexPR | | |  | | |  | |  |
| AII01090.1 | DkikOR21 | | | | ADD14623.1 | | | | | DmauOR7a | | | | NP_001097687.1 | | | | DmelOrco B | | |  | | |  | |  |
| AII01089.1 | DkikOR22 | | | | ACM18060.1 | | | | | MsexOR2 | | | | NP_524235.2 | | | | DmelOrco A | | |  | | |  | |  |
| AII01088.1 | DkikOR23 | | | | AFL70825.1 | | | | | MsexOR62 | | | | AII15784.1 | | | | ScerOrco | | |  | | |  | |  |
| AII01087.1 | DkikOR24 | | | | ADM32898.1 | | | | | MsexOR5 | | | | XP_001359364.3 | | | | DpseOrco | | |  | | |  | |  |
| AII01086.1 | DkikOR25 | | | | ADM32897.1 | | | | | MsexOR4 | | | | AWV67916.1 | | | | LcapOrco | | |  | | |  | |  |
| AII01085.1 | DkikOR26 | | | | ACM18061.1 | | | | | MsexOR3 | | | | AGS41440.1 | | | | AsegOrco | | |  | | |  | |  |
| AII01084.1 | DkikOR27 | | | | CUQ99422.1 | | | | | MsexOrco | | | | AEA30004.2 | | | | CmegOrco | | |  | | |  | |  |
| AII01083.1 | DkikOR28 | | | | CUQ99421.1 | | | | | MsexOR80 | | | | AFH96944.1 | | | | MdomOrco | | |  | | |  | |  |
| AII01082.1 | DkikOR29 | | | | CUQ99419.1 | | | | | MsexOR65 | | | | AFH96943.1 | | | | CrufOrco | | |  | | |  | |  |
| AII01081.1 | DkikOR30 | | | | CUQ99418.1 | | | | | MsexOR42 | | | | ATV96621.1 | | | | EsemOrco | | |  | | |  | |  |
| AII01080.1 | DkikOR31 | | | | CUQ99417.1 | | | | | MsexOR40 | | | | RLZ02183.1 | | | | CcinOrco | | |  | | |  | |  |
| AII01079.1 | DkikOR32 | | | | CUQ99416.1 | | | | | MsexOR36 | | | | AWW05228.1 | | | | CcilOrco | | |  | | |  | |  |
| AII01078.1 | DkikOR33 | | | | CUQ99415.1 | | | | | MsexOR35 | | | | BAO48211.1 | | | | CjapOrco | | |  | | |  | |  |
| AJF20961.1 | ObruPR | | | | CUQ99414.1 | | | | | MsexOR34 | | | | AQY16483.1 | | | | PsauOrco | | |  | | |  | |  |
| ALT31682.1 | CmedPR1 | | | | CUQ99413.1 | | | | | MsexOR33 | | | | ALT31679.1 | | | | CmedOrco | | |  | | |  | |  |
| ALT31681.1 | CmedPR2 | | | | CUQ99412.1 | | | | | MsexOR31 | | | | AJF94638.2 | | | | AquaOrco | | |  | | |  | |  |
| AJG42377.1 | HarmPR | | | | CUQ99411.1 | | | | | MsexOR30 | | | | AIO10777.1 | | | | AfunOrco | | |  | | |  | |  |
| AIZ94619.1 | OnubPR | | | | CUQ99410.1 | | | | | MsexOR29 | | | | AFC91712.1 | | | | CpomOrco | | |  | | |  | |  |
| JAG00306.1 | LhesPR | | | | CUQ99409.1 | | | | | MsexOR27 | | | | AOT85634.1 | | | | MdesOrco | | |  | | |  | |  |
| AJO62238.1 | TmolOR19 | | | | CUQ99408.1 | | | | | MsexOR26 | | | | BAU20240.1 | | | | RspeOrco | | |  | | |  | |  |
| AJO62237.1 | TmolOR18 | | | | CUQ99407.1 | | | | | MsexOR25 | | | | NP_001303637.1 | | | | ClecOrco1 | | |  | | |  | |  |
| AJO62236.1 | TmolOR17 | | | | CUQ99406.1 | | | | | MsexOR24 | | | | AIW42735.1 | | | | ClecOrco2 | | |  | | |  | |  |
| **IR** | | | | | | | | |  |  |  |  |  |  |  |  |  |  |  |  |  |  |  |  |  |  |
| **Name** | | | | **GI** | | | | |  |  |  |  |  |  |  |  |  |  |  |  |  |  |  |  |  |  |
| HarmIRa | | | | 666916257 | | | | |  |  |  |  |  |  |  |  |  |  |  |  |  |  |  |  |  |  |
| HarmIRb | | | | 666916255 | | | | |  |  |  |  |  |  |  |  |  |  |  |  |  |  |  |  |  |  |
| HarmIRc | | | | 666916249 | | | | |  |  |  |  |  |  |  |  |  |  |  |  |  |  |  |  |  |  |
| DmelIR25a | | | | 316994955 | | | | |  |  |  |  |  |  |  |  |  |  |  |  |  |  |  |  |  |  |
| DmelIR10a | | | | 158031786 | | | | |  |  |  |  |  |  |  |  |  |  |  |  |  |  |  |  |  |  |
| DmelIR52c | | | | 21645383 | | | | |  |  |  |  |  |  |  |  |  |  |  |  |  |  |  |  |  |  |
| DmelIR56a | | | | 21626963 | | | | |  |  |  |  |  |  |  |  |  |  |  |  |  |  |  |  |  |  |
| DmelIR67a | | | | 442631348 | | | | |  |  |  |  |  |  |  |  |  |  |  |  |  |  |  |  |  |  |
| DmelIR41a | | | | 442622278 | | | | |  |  |  |  |  |  |  |  |  |  |  |  |  |  |  |  |  |  |
| DmelIR84a | | | | 442617872 | | | | |  |  |  |  |  |  |  |  |  |  |  |  |  |  |  |  |  |  |
| DmelIR11a | | | | 442616103 | | | | |  |  |  |  |  |  |  |  |  |  |  |  |  |  |  |  |  |  |
| DmelIR7b | | | | 442615429 | | | | |  |  |  |  |  |  |  |  |  |  |  |  |  |  |  |  |  |  |
| DmelIR75d | | | | 386771401 | | | | |  |  |  |  |  |  |  |  |  |  |  |  |  |  |  |  |  |  |
| DmelIR94e | | | | 221458656 | | | | |  |  |  |  |  |  |  |  |  |  |  |  |  |  |  |  |  |  |
| DmelIR54a | | | | 221330374 | | | | |  |  |  |  |  |  |  |  |  |  |  |  |  |  |  |  |  |  |
| DmelIR52a | | | | 221330289 | | | | |  |  |  |  |  |  |  |  |  |  |  |  |  |  |  |  |  |  |
| DmelIR7f | | | | 221329768 | | | | |  |  |  |  |  |  |  |  |  |  |  |  |  |  |  |  |  |  |
| DmelIR21a | | | | 161076594 | | | | |  |  |  |  |  |  |  |  |  |  |  |  |  |  |  |  |  |  |
| DmelIR62a | | | | 85725098 | | | | |  |  |  |  |  |  |  |  |  |  |  |  |  |  |  |  |  |  |
| DmelIR60a | | | | 24762594 | | | | |  |  |  |  |  |  |  |  |  |  |  |  |  |  |  |  |  |  |
| DmelIR68b | | | | 24663135 | | | | |  |  |  |  |  |  |  |  |  |  |  |  |  |  |  |  |  |  |
| DmelIR56d | | | | 24655848 | | | | |  |  |  |  |  |  |  |  |  |  |  |  |  |  |  |  |  |  |
| DmelIR51b | | | | 24653793 | | | | |  |  |  |  |  |  |  |  |  |  |  |  |  |  |  |  |  |  |
| DmelIR48b | | | | 24652806 | | | | |  |  |  |  |  |  |  |  |  |  |  |  |  |  |  |  |  |  |
| DmelIR47a | | | | 24652423 | | | | |  |  |  |  |  |  |  |  |  |  |  |  |  |  |  |  |  |  |
| DmelIR85a | | | | 24645175 | | | | |  |  |  |  |  |  |  |  |  |  |  |  |  |  |  |  |  |  |
| DmelIR20a | | | | 24643741 | | | | |  |  |  |  |  |  |  |  |  |  |  |  |  |  |  |  |  |  |
| DmelIR7a | | | | 24640399 | | | | |  |  |  |  |  |  |  |  |  |  |  |  |  |  |  |  |  |  |
| DmelIR76b | | | | 316994957 | | | | |  |  |  |  |  |  |  |  |  |  |  |  |  |  |  |  |  |  |
| DmelIR8a | | | | 316994953 | | | | |  |  |  |  |  |  |  |  |  |  |  |  |  |  |  |  |  |  |
| DmelIR64a | | | | 316994961 | | | | |  |  |  |  |  |  |  |  |  |  |  |  |  |  |  |  |  |  |
| DmelIR75a | | | | 312434883 | | | | |  |  |  |  |  |  |  |  |  |  |  |  |  |  |  |  |  |  |
| DmelIR93a | | | | 440217690 | | | | |  |  |  |  |  |  |  |  |  |  |  |  |  |  |  |  |  |  |
| DmelIR92a | | | | 440217656 | | | | |  |  |  |  |  |  |  |  |  |  |  |  |  |  |  |  |  |  |
| DmelIR87a | | | | 440217387 | | | | |  |  |  |  |  |  |  |  |  |  |  |  |  |  |  |  |  |  |
|  | | | |  | | | | |  |  |  |  |  |  |  |  |  |  |  |  |  |  |  |  |  |  |
| OBP | | | | | | | OBP | | | | | | | | | | OBP | | | | | | OBP | | | |
| **Accession NO.** | | | **Name** | | | | **Accession NO.** | | | | | | **Name** | | | | **Accession NO.** | | | **Name** | | | **Accession NO.** | | **Name** | |
| AAK01304.1 | | | AmelOBP | | | | AFD34183.1 | | | | | | AconPBP2 | | | | EFA07430.1 | | | Tcasmin-C OBP | | | ACX53743.1 | | HvirOBP7 | |
| NP_001035310.1 | | | AmelOBP 7 | | | | AFD34183.1 | | | | | | AconPBP3 | | | | EFA05742.1 | | | TcasOBP4 | | | ACX53711.1 | | HvirOBP8 | |
| XP_026298378.1 | | | AmelOBP 6 X1 | | | | AFD34176.1 | | | | | | AconPBP1 | | | | EFA05695.1 | | | TcasOBP11 | | | ACX53696.1 | | HvirOBP9 | |
| XP_016770832.2 | | | AmelOBP 12 X1 | | | | AAP57469.1 | | | | | | AsegPBP | | | | EFA05678.1 | | | TcasOBP1 | | | CAA65606.1 | | HvirGOBP2 | |
| XP_016772293.1 | | | AmelOBP 19 X1 | | | | AAP57468.1 | | | | | | AipsPBP1 | | | | EFA05677.1 | | | TcasOBP5 | | | CAA65605.1 | | HvirGOBP1 | |
| XP_016771954.1 | | | AmelOBP 9 X1 | | | | AAP57467.1 | | | | | | AipsPBP2 | | | | EFA05675.1 | | | TcasOBP3 | | | AGM38613.1 | | CsupOBP5 | |
| XP_006566010.1 | | | AmelOBP 10 X1 | | | | BAH36763.1 | | | | | | BmorOBP7 | | | | EFA04594.1 | | | TcasOBP6 | | | AGM38612.1 | | CsupOBP6 | |
| XP_006567396.1 | | | AmelOBP 3 X1 | | | | BAH36762.1 | | | | | | BmorOBP8 | | | | EFA04593.1 | | | TcasOBP7 | | | AGM38611.1 | | CsupOBP7 | |
| NP_001164515.1 | | | AmelOBP 8 | | | | BAH36761.1 | | | | | | BmorOBP9 | | | | EFA04576.1 | | | TcasOBP24 | | | AGM38610.1 | | CsupOBP8 | |
| AAL60422.1 | | | AmelOBP 5 | | | | BAH36761.1 | | | | | | BmorOBP10 | | | | EFA02960.1 | | | TcasOBP19 | | | AGM38609.1 | | CsupOBP9 | |
| AAL60421.1 | | | AmelOBP 6 | | | | BAH36760.1 | | | | | | BmorOBP11 | | | | EFA02914.1 | | | TcasOBP14 | | | AGM38608.1 | | CsupOBP10 | |
| AAL60420.1 | | | AmelOBP 4 | | | | BAH36759.1 | | | | | | BmorOBP12 | | | | EFA02861.1 | | | TcasOBP17 | | | AGM38607.1 | | CsupOBP11 | |
| AAL60419.1 | | | AmelOBP 1 | | | | BAI44701.1 | | | | | | BmorOBP13 | | | | EFA02860.1 | | | TcasOBP18 | | | AGM38606.1 | | CsupOBP12 | |
| AAL60418.1 | | | AmelOBP 2 | | | | BAI44700.1 | | | | | | BmorOBP14 | | | | EFA02858.1 | | | TcasOBP13 | | | AGM38605.1 | | CsupOBP13 | |
| AAL60417.1 | | | AmelOBP 4 | | | | BAI22690.1 | | | | | | BmorOBP15 | | | | EFA02857.1 | | | TcasOBP12 | | | ADD71058.1 | | CsupOBP14 | |
| AAF06123.1 | | | SexiPBP | | | | BAI22689.1 | | | | | | BmorOBP16 | | | | AII01010.1 | | | DkikOBP1 | | | ACJ07120.1 | | CsupGOBP2 | |
| AAF06144.1 | | | CmurPBP4 | | | | BAH79159.1 | | | | | | BmorOBP17 | | | | AII01009.1 | | | DkikOBP2 | | | ACJ07127.1 | | CsupGOBP1 | |
| AAF06143.1 | | | YcagPBP | | | | CAA64445.1 | | | | | | BmorGOBP2 | | | | AII01008.1 | | | DkikOBP3 | | | ANZ73034.1 | | CsupOBP4 | |
| AAF06142.1 | | | SexiPBP | | | | CAA64444.1 | | | | | | BmorGOBP1 | | | | AII01006.1 | | | DkikOBP4 | | | ANZ73033.1 | | CsupOBP1 | |
| AAF06141.1 | | | PgosPBP | | | | NP_001140190.1 | | | | | | BmorOBP6 | | | | AII01004.1 | | | DkikOBP5 | | |  | |  | |
| AAF06140.1 | | | CrosPBP1 | | | | NP_001140188.1 | | | | | | BmorOBP4 | | | | AII01002.1 | | | DkikOBP6 | | |  | |  | |
| AAF06139.1 | | | CrosPBP2 | | | | NP_001140185.1 | | | | | | BmorOBP1 | | | | AII01001.1 | | | DkikOBP7 | | |  | |  | |
| AAF06138.1 | | | CpinPBP1 | | | | NP_001159622.2 | | | | | | BmorOBP56d | | | | AII01000.1 | | | DkikOBP8 | | |  | |  | |
| AAF06137.1 | | | CrosPBP | | | | XP_021204926.1 | | | | | | BmorOBP1X1 | | | | AII00999.1 | | | DkikOBP9 | | |  | |  | |
| AAF06136.1 | | | Cpin PBP2 | | | | XP_012547442.1 | | | | | | BmorOBP6X1 | | | | AII00998.1 | | | DkikOBP10 | | |  | |  | |
| AAF06135.1 | | | CpinPBP3 | | | | XP_012547441.1 | | | | | | BmorOBP4X1 | | | | AII00997.1 | | | DkikOBP11 | | |  | |  | |
| AAF06134.1 | | | CparPBP1 | | | | CAS90130.1 | | | | | | BmorOBP6 | | | | AII00995.1 | | | DkikOBP12 | | |  | |  | |
| AAF06133.1 | | | CparPBP2 | | | | CAS90128.1 | | | | | | BmorOBP4 | | | | AII00994.1 | | | DkikOBP13 | | |  | |  | |
| AAF06132.1 | | | CmurPBP1 | | | | CAS90125.1 | | | | | | BmorOBP1 | | | | AII00993.1 | | | DkikOBP14 | | |  | |  | |
| AAF06131.1 | | | CmurPBP2 | | | | EFA02853.2 | | | | | | TcasOBP16 | | | | AII00992.1 | | | DkikOBP15 | | |  | |  | |
| AAF06130.1 | | | CmurPBP3 | | | | EFA04746.2 | | | | | | TcasOBP26 | | | | AII00991.1 | | | DkikOBP16 | | |  | |  | |
| AAF06129.1 | | | CfumPBP1 | | | | EFA04747.2 | | | | | | TcasOBP25 | | | | AII00990.1 | | | DkikOBP17 | | |  | |  | |
| AAF06128.1 | | | CfumPBP2 | | | | EFA04687.2 | | | | | | TcasOBP8 | | | | AII00989.1 | | | DkikOBP18 | | |  | |  | |
| AAF06127.1 | | | CfumPBP3 | | | | EFA05793.2 | | | | | | TcasOBP20 | | | | AII00988.1 | | | DkikOBP19 | | |  | |  | |
| AAF06126.1 | | | AvelPBP1 | | | | EFA05676.2 | | | | | | TcasOBP2 | | | | AGJ83357.1 | | | DkikGOBP1 | | |  | |  | |
| AAF06125.1 | | | AvelPBP2 | | | | EFA09215.2 | | | | | | TcasOBP21 | | | | AGJ83353.1 | | | DkikGOBP2 | | |  | |  | |
| AAF06124.1 | | | AvelPBP3 | | | | EFA09155.2 | | | | | | TcasOBP22 | | | | ACX53761.1 | | | HvirOBP1 | | |  | |  | |
| AAC36315.1 | | | HzeaPBP | | | | EFA12066.1 | | | | | | TcasOBP15 | | | | ACX53748.1 | | | HvirOBP2 | | |  | |  | |
| BAF64703.1 | | | AselPBP2 | | | | EFA10803.1 | | | | | | TcasOBP23 | | | | ACX53819.1 | | | HvirOBP3 | | |  | |  | |
| AAX85460.1 | | | AsegPBP2 | | | | EFA10713.1 | | | | | | TcasOBP9 | | | | ACX53795.1 | | | HvirOBP4 | | |  | |  | |
| AAX85459.1 | | | AipsPBP2 | | | | EFA07544.1 | | | | | | Tcas min-C OBP | | | | ACX53756.1 | | | HvirOBP5 | | |  | |  | |
| AAX85458.1 | | | AipsPBP1 | | | | EFA07542.1 | | | | | | TcasOBP10 | | | | ACX53747.1 | | | HvirOBP6 | | |  | |  | |
| GR | | | | | | | | GR | | | | | | | |  |  |  |  |  |  |  |  |  |  |  |
| **GI** | | **Name** | | | | | | **GI** | | | | **Name** | | | |  |  |  |  |  |  |  |  |  |  |  |
| 429545903 | | HarmGRa | | | | | | 47117920 | | | | DmelGR43a | | | |  |  |  |  |  |  |  |  |  |  |  |
| 666916233 | | HarmGRb | | | | | | 17986119 | | | | DmelGR61a | | | |  |  |  |  |  |  |  |  |  |  |  |
| 666916229 | | HarmGRc | | | | | | 24657115 | | | | DmelGR64a | | | |  |  |  |  |  |  |  |  |  |  |  |
| 666916227 | | HarmGRd | | | | | | 118500892 | | | | DmelGR21a | | | |  |  |  |  |  |  |  |  |  |  |  |
| 666916225 | | HarmGRe | | | | | | 24639922 | | | | DmelGR5a | | | |  |  |  |  |  |  |  |  |  |  |  |
| 486139707 | | HarmGR5 | | | | | | 281365837 | | | | DmelGR66a | | | |  |  |  |  |  |  |  |  |  |  |  |
| 486139682 | | HarmGR4 | | | | | | 221330835 | | | | DmelGR63a | | | |  |  |  |  |  |  |  |  |  |  |  |
| 486139658 | | HarmGR1 | | | | | | 45549158 | | | | DmelGR32a | | | |  |  |  |  |  |  |  |  |  |  |  |
| 221136983 | | TcasGRa | | | | | | 45551511 | | | | DmelGR64f | | | |  |  |  |  |  |  |  |  |  |  |  |
| 163716784 | | TcasGRb | | | | | | 24662881 | | | | DmelGR68a | | | |  |  |  |  |  |  |  |  |  |  |  |
| 163716754 | | TcasGRc | | | | | | 45549284 | | | | DmelGR33a | | | |  |  |  |  |  |  |  |  |  |  |  |
| 270006491 | | TcasGR178 | | | | | | 24648814 | | | | DmelGR93a | | | |  |  |  |  |  |  |  |  |  |  |  |
| 270006483 | | TcasGR 97 | | | | | | 17986103 | | | | DmelGR57a | | | |  |  |  |  |  |  |  |  |  |  |  |
| 270006476 | | TcasGR 2 | | | | | | 45549155 | | | | DmelGR28a | | | |  |  |  |  |  |  |  |  |  |  |  |
| 270015820 | | TcasGR 26 | | | | | | 24641287 | | | | DmelGR10a | | | |  |  |  |  |  |  |  |  |  |  |  |
| 270015819 | | TcasGR 27 | | | | | | 28571153 | | | | DmelGR8a | | | |  |  |  |  |  |  |  |  |  |  |  |
| 270011186 | | TcasGR 168 | | | | | | 28573623 | | | | DmelGR59f | | | |  |  |  |  |  |  |  |  |  |  |  |
| 270011184 | | TcasGR 154 | | | | | | 24585658 | | | | DmelGR39b | | | |  |  |  |  |  |  |  |  |  |  |  |
| 270011183 | | TcasGR 153 | | | | | | 24649189 | | | | DmelGR94a | | | |  |  |  |  |  |  |  |  |  |  |  |
| 270011173 | | TcasGR 125 | | | | | | 28573606 | | | | DmelGR58c | | | |  |  |  |  |  |  |  |  |  |  |  |
| 270011167 | | TcasGR 118 | | | | | | 45550994 | | | | DmelGR36a | | | |  |  |  |  |  |  |  |  |  |  |  |
| 270011166 | | TcasGR 117 | | | | | | 24580943 | | | | DmelGR22a | | | |  |  |  |  |  |  |  |  |  |  |  |
| 270011146 | | TcasGR 1 | | | | | | 24667642 | | | | DmelGR77a | | | |  |  |  |  |  |  |  |  |  |  |  |
| 270009311 | | TcasGR 24 | | | | | | 45551079 | | | | DmelGR47b | | | |  |  |  |  |  |  |  |  |  |  |  |
| 270008279 | | TcasGR 166 | | | | | | 45550757 | | | | DmelGR89a | | | |  |  |  |  |  |  |  |  |  |  |  |
| 270008276 | | TcasGR 18 | | | | | | 24650640 | | | | DmelGR98d | | | |  |  |  |  |  |  |  |  |  |  |  |
| 270008272 | | TcasGR 14 | | | | | | 45551934 | | | | DmelGR92a | | | |  |  |  |  |  |  |  |  |  |  |  |
| 270008271 | | TcasGR 13 | | | | | | 24645557 | | | | DmelGR85a | | | |  |  |  |  |  |  |  |  |  |  |  |
| 270008270 | | TcasGR 12 | | | | | | 17986021 | | | | DmelGR10b | | | |  |  |  |  |  |  |  |  |  |  |  |
| 270008269 | | TcasGR 11 | | | | | | 24640947 | | | | DmelGR9a | | | |  |  |  |  |  |  |  |  |  |  |  |
| 270008268 | | TcasGR 10 | | | | | | 350536295 | | | | BmorGR68 | | | |  |  |  |  |  |  |  |  |  |  |  |
| 270008265 | | TcasGR 7 | | | | | | 350536275 | | | | BmorGR67 | | | |  |  |  |  |  |  |  |  |  |  |  |
| 270008263 | | TcasGR 5 | | | | | | 195963349 | | | | BmorGR60 | | | |  |  |  |  |  |  |  |  |  |  |  |
| 270008261 | | TcasGR 3 | | | | | | 195963347 | | | | BmorGR45 | | | |  |  |  |  |  |  |  |  |  |  |  |
| 270002945 | | TcasGR 164 | | | | | | 195963345 | | | | BmorGR9 | | | |  |  |  |  |  |  |  |  |  |  |  |
| 270002937 | | TcasGR 87 | | | | | | 195963343 | | | | BmorGR8 | | | |  |  |  |  |  |  |  |  |  |  |  |
| 224458358 | | TcasGR82 | | | | | | 912719236 | | | | BmorGR10 | | | |  |  |  |  |  |  |  |  |  |  |  |
| 571550487 | | AmelGR7 | | | | | |  | | | |  | | | |  |  |  |  |  |  |  |  |  |  |  |
| 339715206 | | AmelGR10 | | | | | |  | | | |  | | | |  |  |  |  |  |  |  |  |  |  |  |
| 571550434 | | AmelGR32 | | | | | |  | | | |  | | | |  |  |  |  |  |  |  |  |  |  |  |
| 571529490 | | AmelGR64f | | | | | |  | | | |  | | | |  |  |  |  |  |  |  |  |  |  |  |
| 328778564 | | AmelGR43a | | | | | |  | | | |  | | | |  |  |  |  |  |  |  |  |  |  |  |
