# Supplementary material for: The whole body transcriptome of Coleophora obducta reveals important olfactory proteins
Source: PeerJ. 2020 Apr 10;8:e8902. doi: 10.7717/peerj.8902 (PMC7153557; doi:10.7717/peerj.8902)
Supplement: Supplemental Information 3 [file peerj-08-8902-s003.docx]

**The whole body transcriptome of *Coleophora obducta* reveals important olfactory proteins**

Dongbai Wang^2^, Jing Tao^3^, Pengfei Lu^3^, Youqing Luo^3^, Ping Hu^1,2^

^1^ Guangxi University, Nanning, Guangxi, China

^2^ Xingan Vocational and Technical College, Xinganmeng, Inner mongolia, China

^3^ Beijing Key Laboratory for Forest Pest Control, Beijing Forestry University, Beijing, China

**Supplementary file 3**

**Primer used for fluorescence quantitative real-time PCR**

| Name | Forward primer | Reverse primer |
| --- | --- | --- |
| β-actin | AGAAGCACTTGCGGTGGACAAT | ACCTGTACGCCAACACTGTCAT |
| CobdPBP1 | GAAGAAACTGACGATGGGATT | CAGGTCGAACTTGGAGGC |
| CobdPBP2 | AAGTGTTAGTAAATGTGGTGGTGT | CAAGAAGGTTGAGTTTGTGGC |
| CobdPBP3 | TGGCTCCTGATGAAGTGCT | AAGTGGTCCCGATAACGAA |
