# Supplementary material for: The whole body transcriptome of Coleophora obducta reveals important olfactory proteins
Source: PeerJ. 2020 Apr 10;8:e8902. doi: 10.7717/peerj.8902 (PMC7153557; doi:10.7717/peerj.8902)
Supplement: Supplemental Information 4 [file peerj-08-8902-s004.docx]

**The whole body transcriptome of *Coleophora obducta* reveals important olfactory proteins**

Dongbai Wang^2^, Jing Tao^3^, Pengfei Lu^3^, Youqing Luo^3^, Ping Hu^1,2^

^1^ Guangxi University, Nanning, Guangxi, China

^2^ Xingan Vocational and Technical College, Xinganmeng, Inner mongolia, China

^3^ Beijing Key Laboratory for Forest Pest Control, Beijing Forestry University, Beijing, China

**Supplementary file 4 Putative olfactory protein in the** **whole body transcriptome of *C. obducta***

|  | | | |  |  | **Best Blast Match** | | | |
| --- | --- | --- | --- | --- | --- | --- | --- | --- | --- |
| **Number** | **Gene ID** | **Gene length(bp)** | **ORF length(bp)** | **FPKM** |  | **Name** | **ACC. Number** | **Speceies** | **E-value** |
| CobdGOBP1 | TRINITY_DN10193_c4_g1 | 1888 | 501 | 86.05 |  | general odorant binding protein | AOG12859.1 | *Eogystia hippophaecolus* | 6.10E-61 |
| CobdOBP1 | TRINITY_DN10393_c1_g2 | 822 | 219 | 20.65 |  | Odorant Binding Protein 38 | ARO70197.1 | *Dendrolimus punctatus* | 1.10E-06 |
| CobdOBP2 | TRINITY_DN10942_c3_g4 | 1072 | 303 | 5.28 |  | odorant binding protein 16 | AVZ44709.1 | *Grapholita molesta* | 5.90E-69 |
| CobdOBP3 | TRINITY_DN12399_c3_g2 | 464 | 408 | 17.69 |  | odorant binding protein | AOG12879.1 | *Eogystia hippophaecolus* | 1.90E-40 |
| CobdOBP4 | TRINITY_DN14180_c1_g1 | 402 | 327 | 197.37 |  | odorant binding protein 16 | ALD65890.1 | *Spodoptera litura* | 1.00E-13 |
| CobdOBP5 | TRINITY_DN14253_c1_g7 | 837 | 540 | 21.43 |  | odorant binding protein | AOG12877.1 | *Eogystia hippophaecolus* | 7.70E-40 |
| CobdOBP6 | TRINITY_DN3399_c0_g1 | 579 | 573 | 1.92 |  | odorant binding protein 9 | ALD65883.1 | *Spodoptera litura* | 4.40E-10 |
| CobdOBP7 | TRINITY_DN5958_c0_g1 | 587 | 459 | 3.75 |  | odorant binding protein LOC100301496 precursor | NP_001153664.1 | *Bombyx mori* | 2.50E-45 |
| CobdOBP8 | TRINITY_DN6761_c0_g1 | 947 | 759 | 24.95 |  | odorant binding protein | AOG12867.1 | *Eogystia hippophaecolus* | 7.50E-84 |
| CobdOBP9 | TRINITY_DN8533_c0_g1 | 859 | 720 | 6.35 |  | odorant binding protein | AOG12855.1 | *Eogystia hippophaecolus* | 3.00E-55 |
| CobdGOBP2 | TRINITY_DN8926_c1_g4 | 808 | 489 | 178.96 |  | general odorant binding protein | AEZ52491.1 | *Orthaga achatina* | 1.30E-63 |
| CobdOBP10 | TRINITY_DN9234_c2_g2 | 461 | 423 | 12.12 |  | odorant binding protein | AOG12873.1 | *Eogystia hippophaecolus* | 5.80E-58 |
| CobdOBP11 | TRINITY_DN6480_c0_g1 | 715 | 459 | 59.70 |  | odorant-binding protein 4 | AGK24580.1 | *Chilo suppressalis* | 9.70E-60 |
| CobdPBP1 | TRINITY_DN10947_c2_g3 | 831 | 465 | 23.86 |  | pheromone binding protein 2 | AKA27976.1 | *Atrijuglans hetaohei* | 3.00E-60 |
| CobdPBP2 | TRINITY_DN6722_c0_g1 | 804 | 495 | 5.00 |  | pheromone-binding protein 3 | AHZ89399.1 | *Grapholita molesta* | 4.20E-51 |
| CobdPBP3 | TRINITY_DN7344_c0_g1 | 1377 | 543 | 9.08 |  | pheromone binding protein 2 | ACJ07123.1 | *Chilo suppressalis* | 1.40E-14 |
| CobdCSP1 | TRINITY_DN9951_c0_g1 | 968 | 336 | 72.92 |  | chemosensory protein 20 | AKT26494.1 | *Spodoptera exigua* | 9.50E-42 |
| CobdCSP2 | TRINITY_DN9566_c1_g1 | 553 | 381 | 9.97 |  | chemosensory protein 3 | AGR39573.1 | *Agrotis ipsilon* | 1.90E-34 |
| CobdCSP3 | TRINITY_DN8861_c1_g4 | 771 | 390 | 370.89 |  | chemosensory protein | ABW34383.1 | *Pieris rapae* | 1.90E-40 |
| CobdCSP4 | TRINITY_DN8789_c1_g1 | 769 | 129 | 92.22 |  | chemosensory protein | AIX97838.1 | *Cnaphalocrocis medinalis* | 3.00E-14 |
| CobdCSP5 | TRINITY_DN8204_c1_g1 | 1428 | 966 | 18.71 |  | chemosensory protein 14 | AKT26490.1 | *Spodoptera exigua* | 3.80E-55 |
| CobdCSP6 | TRINITY_DN5965_c0_g1 | 525 | 360 | 32.30 |  | chemosensory protein 7 precursor | NP_001037068.1 | *Bombyx mori* | 4.50E-30 |
| CobdCSP7 | TRINITY_DN4941_c0_g2 | 276 | 228 | 1.43 |  | putative chemosensory receptor 4, partial | CAD31946.1 | *Heliothis virescens* | 5.80E-37 |
| CobdCSP8 | TRINITY_DN14546_c1_g4 | 1019 | 330 | 2.56 |  | chemosensory protein | AIX97829.1 | *Cnaphalocrocis medinalis* | 7.90E-47 |
| CobdCSP9 | TRINITY_DN14335_c4_g2 | 988 | 369 | 12.72 |  | chemosensory protein 5 | BAV56809.1 | *Ostrinia furnacalis* | 2.10E-44 |
| CobdCSP10 | TRINITY_DN13445_c0_g1 | 744 | 324 | 79.19 |  | chemosensory protein | APG32548.1 | *Conogethes punctiferalis* | 4.00E-16 |
| CobdCSP11 | TRINITY_DN12620_c1_g1 | 2535 | 267 | 4.33 |  | putative chemosensory receptor 2 | AAW52583.1 | *Spodoptera exigua* | 2.30E-236 |
| CobdCSP12 | TRINITY_DN12289_c0_g1 | 437 | 342 | 1.85 |  | chemosensory protein 8 | AKT26485.1 | *Spodoptera exigua* | 3.20E-37 |
| CobdCSP13 | TRINITY_DN10385_c3_g2 | 685 | 645 | 1.70 |  | putative chemosensory receptor 13 | CAG38114.1 | *Heliothis virescens* | 2.60E-46 |
| CobdCSP14 | TRINITY_DN10019_c5_g4 | 699 | 384 | 379.12 |  | chemosensory protein 7 | BAV56811.1 | *Ostrinia furnacalis* | 1.40E-39 |
| CobdPR1(CobdOR1) | TRINITY_DN10385_c3_g2 | 685 | 645 | 1.70 |  | putative odorant receptor | AGY14585.2 | *Sesamia inferens* | 8.20E-48 |
| CobdOR2 | TRINITY_DN10828_c0_g4 | 254 | 234 | 2.72 |  | odorant receptor 4 | XP_021208172.1 | *Bombyx mori* | 1.00E-11 |
| CobdPR2(CobdOR3) | TRINITY_DN11086_c0_g2 | 1648 | 1296 | 3.39 |  | odorant receptor 13a-like | NP_001292415.1 | *Plutella xylostella* | 7.70E-76 |
| CobdPR3(CobdOR4) | TRINITY_DN11086_c0_g4 | 604 | 525 | 1.72 |  | PREDICTEDodorant receptor 13a-like | XP_011564712.1 | *Plutella xylostella* | 2.30E-38 |
| CobdOR5 | TRINITY_DN12061_c0_g6 | 577 | 366 | 6.23 |  | putative odorant receptor OR29 | AST36264.1 | *Hedya nubiferana* | 2.50E-58 |
| CobdOR6 | TRINITY_DN1239_c0_g1 | 376 | 93 | 1.32 |  | odorant receptor 4 isoform X2 | XP_021205150.1 | *Bombyx mori* | 1.00E-20 |
| CobdOrco | TRINITY_DN12620_c1_g1 | 2535 | 1425 | 4.33 |  | odorant receptor co-receptor | AII15784.1 | *Sitotroga cerealella* | 3.20E-238 |
| CobdOR7 | TRINITY_DN17301_c0_g1 | 342 | 237 | 1.25 |  | Odorant Receptor 40 | ARO70252.1 | *Dendrolimus punctatus* | 6.50E-30 |
| CobdOR8 | TRINITY_DN5607_c0_g1 | 444 | 81 | 2.23 |  | odorant receptor, partial | AIG51872.1 | *Helicoverpa armigera* | 7.00E-08 |
| CobdOR9 | TRINITY_DN8037_c1_g5 | 406 | 141 | 7.95 |  | Odorant receptor, partial | KOB65086.1 | *Operophtera brumata* | 9.20E-07 |
| CobdOR10 | TRINITY_DN8149_c2_g3 | 1387 | 216 | 19.56 |  | odorant receptor, partial | AIG51872.1 | *Helicoverpa armigera* | 4.40E-24 |
| CobdOR11 | TRINITY_DN9175_c3_g1 | 2849 | 1215 | 4.93 |  | odorant receptor 17 | ARO76423.1 | *Conogethes punctiferalis* | 4.70E-166 |
| CobdOR12 | TRINITY_DN9366_c2_g1 | 1384 | 693 | 24.94 |  | odorant receptor 60 | ALM26243.1 | *Athetis dissimilis* | 3.20E-83 |
| CobdIR25a | TRINITY_DN11261_c2_g2 | 3717 | 2898 | 12.86 |  | putative ionotropic receptor IR25a | AQM73611.1 | *Cydia nigricana* | 0 |
| CobdIR75p2(CobdIR1) | TRINITY_DN11626_c1_g1 | 2128 | 1503 | 8.22 |  | ionotropic receptor | BAR64805.1 | *Ostrinia furnacalis* | 2.60E-153 |
| CobdIR64a(CobdIR2) | TRINITY_DN12976_c3_g2 | 1721 | 1116 | 1.70 |  | ionotropic receptor | BAR64801.1 | *Ostrinia furnacalis* | 2.10E-116 |
| CobdIR93a | TRINITY_DN13522_c1_g1 | 2343 | 2166 | 2.21 |  | ionotropic receptor 93a | XP_021190111.1 | *Helicoverpa armigera* | 0 |
| CobdIR62a(CobdIR3) | TRINITY_DN4307_c0_g2 | 1226 | 972 | 1.60 |  | ionotropic receptor 60a1b, partial | AMM70740.1 | *Heliconius timareta* | 9.40E-87 |
| CobdIR68a(CobdIR4) | TRINITY_DN7754_c0_g1 | 1871 | 1548 | 23.73 |  | ionotropic receptor | BAR64816.1 | *Ostrinia furnacalis* | 1.10E-200 |
| CobdIR76b(CobdIR5) | TRINITY_DN8192_c1_g1 | 1857 | 1626 | 25.69 |  | ionotropic receptor | AOG12850.1 | *Eogystia hippophaecolus* | 3.60E-207 |
| CobdGR1 | TRINITY_DN8311_c0_g1 | 771 | 357 | 172.98 |  | gustatory receptor | AOG12970.1 | *Eogystia hippophaecolus* | 4.00E-19 |
| CobdGR64 | TRINITY_DN7854_c1_g1 | 442 | 333 | 70.34 |  | gustatory receptor for sugar taste 64e-like | XP_013148432.1 | *Papilio polytes* | 1.20E-12 |
| CobdGR43a | TRINITY_DN4941_c0_g2 | 276 | 228 | 1.43 |  | gustatory receptor for sugar taste 43a-like | XP_021196329.1 | *Helicoverpa armigera* | 5.80E-37 |
| CobdGR2 | TRINITY_DN4584_c0_g2 | 372 | 138 | 1.44 |  | gustatory receptor 2, partial | ALM26252.1 | *Athetis dissimilis* | 1.00E-28 |
| CobdGR3 | TRINITY_DN16452_c0_g1 | 235 | 138 | 3.14 |  | antennal gustatory receptor 12 | ARO70284.1 | *Dendrolimus punctatus* | 3.30E-09 |
| CobdGR4 | TRINITY_DN14538_c0_g2 | 1778 | 954 | 9.92 |  | gustatory receptor 2, partial | ALM26252.1 | *Athetis dissimilis* | 5.90E-90 |
| CobdGR5 | TRINITY_DN14422_c2_g1 | 1192 | 867 | 5.27 |  | gustatory receptor 2, partial | ALM26252.1 | *Athetis dissimilis* | 1.30E-40 |
| CobdGR6 | TRINITY_DN13413_c1_g3 | 1449 | 753 | 1.40 |  | gustatory receptor 2, partial | ALM26252.1 | *Athetis dissimilis* | 6.70E-100 |
| CobdGR7 | TRINITY_DN12116_c0_g7 | 772 | 408 | 4.24 |  | gustatory receptor 4 | ASW18693.1 | *Helicoverpa armigera* | 4.00E-51 |
| CobdGR8 | TRINITY_DN11423_c2_g3 | 733 | 636 | 2.70 |  | gustatory receptor 2, partial | ALM26252.1 | *Athetis dissimilis* | 1.90E-50 |
| CobdODE1 | TRINITY_DN10244_c0_g3 | 2107 | 1491 | 6.51 |  | odorant degrading enzyme CXE14 | AII21988.1 | *Sesamia inferens* | 8.10E-171 |
| CobdODE2 | TRINITY_DN9716_c0_g1 | 2422 | 1629 | 6.30 |  | odorant degrading enzyme CXE10 | AII21984.1 | *Sesamia inferens* | 2.40E-174 |
| CobdODE3 | TRINITY_DN10903_c0_g2 | 1868 | 1629 | 20.22 |  | odorant degrading enzyme CXE18 | AII21990.1 | *Sesamia inferens* | 2.20E-196 |
| CobdODE4 | TRINITY_DN12476_c3_g2 | 1912 | 1677 | 25.07 |  | odorant degrading enzyme CXE13 | AII21987.1 | *Sesamia inferens* | 5.10E-241 |
| CobdODE5 | TRINITY_DN10817_c2_g3 | 2163 | 1677 | 4.43 |  | odorant degrading enzyme CXE9 | AII21983.1 | *Sesamia inferens* | 7.70E-209 |
| CobdODE6 | TRINITY_DN10817_c2_g4 | 1965 | 1617 | 8.85 |  | odorant degrading enzyme CXE3 | AII21980.1 | *Sesamia inferens* | 1.10E-201 |
| CobdCXE1 | TRINITY_DN10261_c0_g1 | 2017 | 1632 | 7.34 |  | antennal esterase CXE12 | AMB19665.1 | *Cydia pomonella]* | 4.10E-204 |
| CobdCXE2 | TRINITY_DN10998_c0_g1 | 3257 | 2070 | 86.89 |  | antennal oxidoreductase | AAR26515.1 | *Mamestra brassicae* | 0 |
| CobdCXE3 | TRINITY_DN13764_c3_g1 | 3208 | 1695 | 9.72 |  | Antennal esterase CXE14 | KOB65096.1 | *Operophtera brumata* | 2.30E-153 |
| CobdCXE4 | TRINITY_DN16765_c0_g1 | 260 | 213 | 2.86 |  | Antennal esterase CXE19 | KOB70665.1 | *Operophtera brumata* | 1.10E-29 |
| CobdCXE5 | TRINITY_DN9092_c1_g1 | 1447 | 1113 | 6.45 |  | antennal carboxylesterase 18, partial | AKS40370.1 | *Chilo suppressalis* | 2.70E-101 |
| CobdSNMP3 | TRINITY_DN11604_c0_g1 | 737 | 575 | 3.78 |  | PREDICTED: sensory neuron membrane protein 2-like | XP_014366457.1 | *Papilio machaon* | 2.00E-60 |
| CobdSNMP1 | TRINITY_DN13248_c0_g1 | 2403 | 1593 | 6.80 |  | sensory neuron membrane protein 1-like | XP_026331738.1 | *Hyposmocoma kahamanoa* | 0.00E+00 |
| CobdSNMP2 | TRINITY_DN7754_c0_g1 | 1871 | 1566 | 23.73 |  | RecName: Full=Sensory neuron membrane protein 2 | E5EZW9.1 | *Ostrinia nubilalis* | 0.00E+00 |
