# Supplementary figures and images for: The whole body transcriptome of Coleophora obducta reveals important olfactory proteins
Source: PeerJ. 2020 Apr 10;8:e8902. doi: 10.7717/peerj.8902 (PMC7153557; doi:10.7717/peerj.8902)

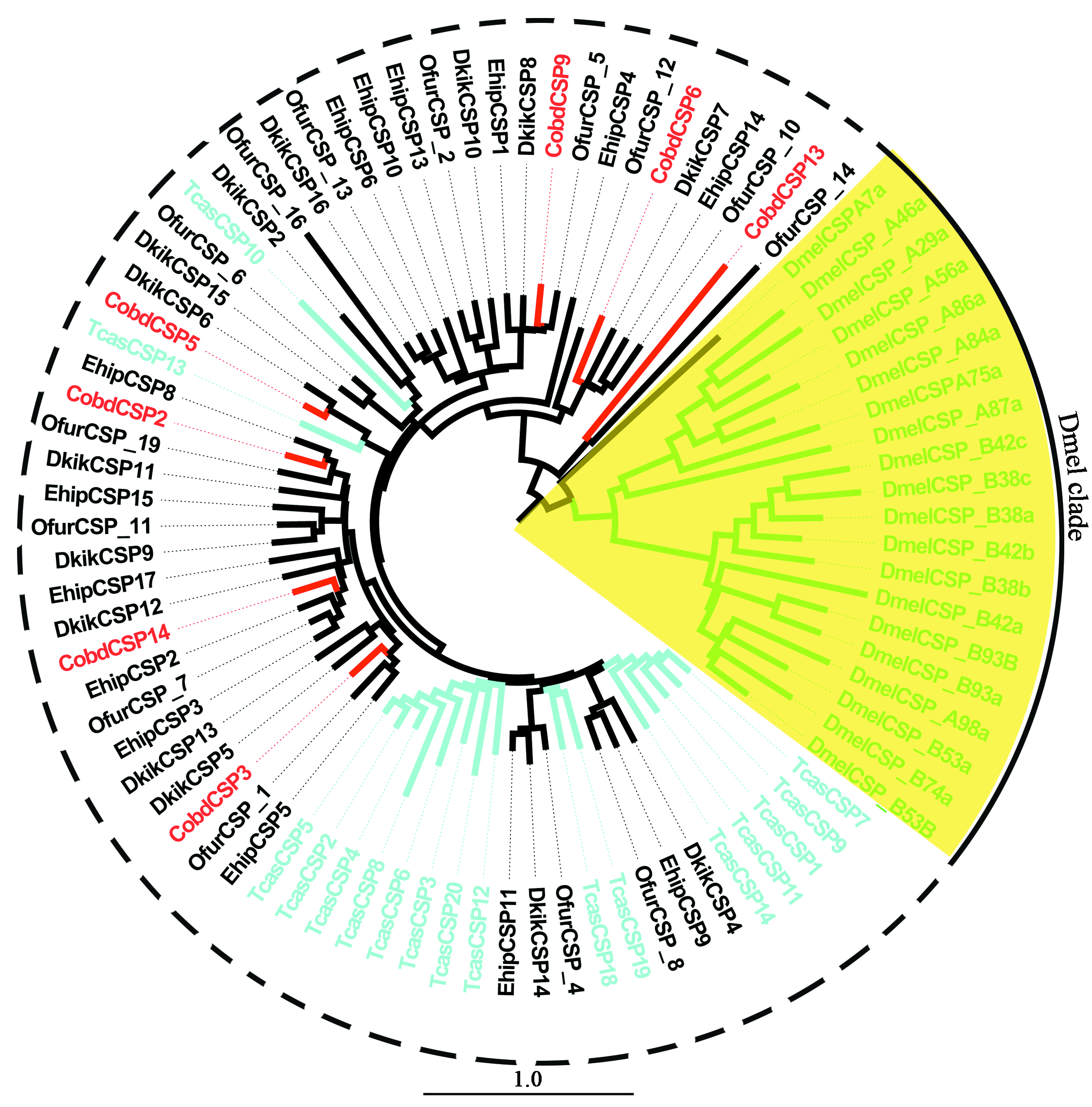

Supplement: Supplemental Information 5 — The NJ phylogenetic analysis of CSPs of C. obducta (CobdCSP, red) was performed with reference CSPs of D. melanogaster (DmelCSP, Diptera), Tribolium castaneum (TcasCSP, Coleoptera) and CSPs of Lepidoptera species. Yellow fill area refers to Diptera clade. The scale bar represents 1.0 substitutions per site. [file peerj-08-8902-s005.jpg]

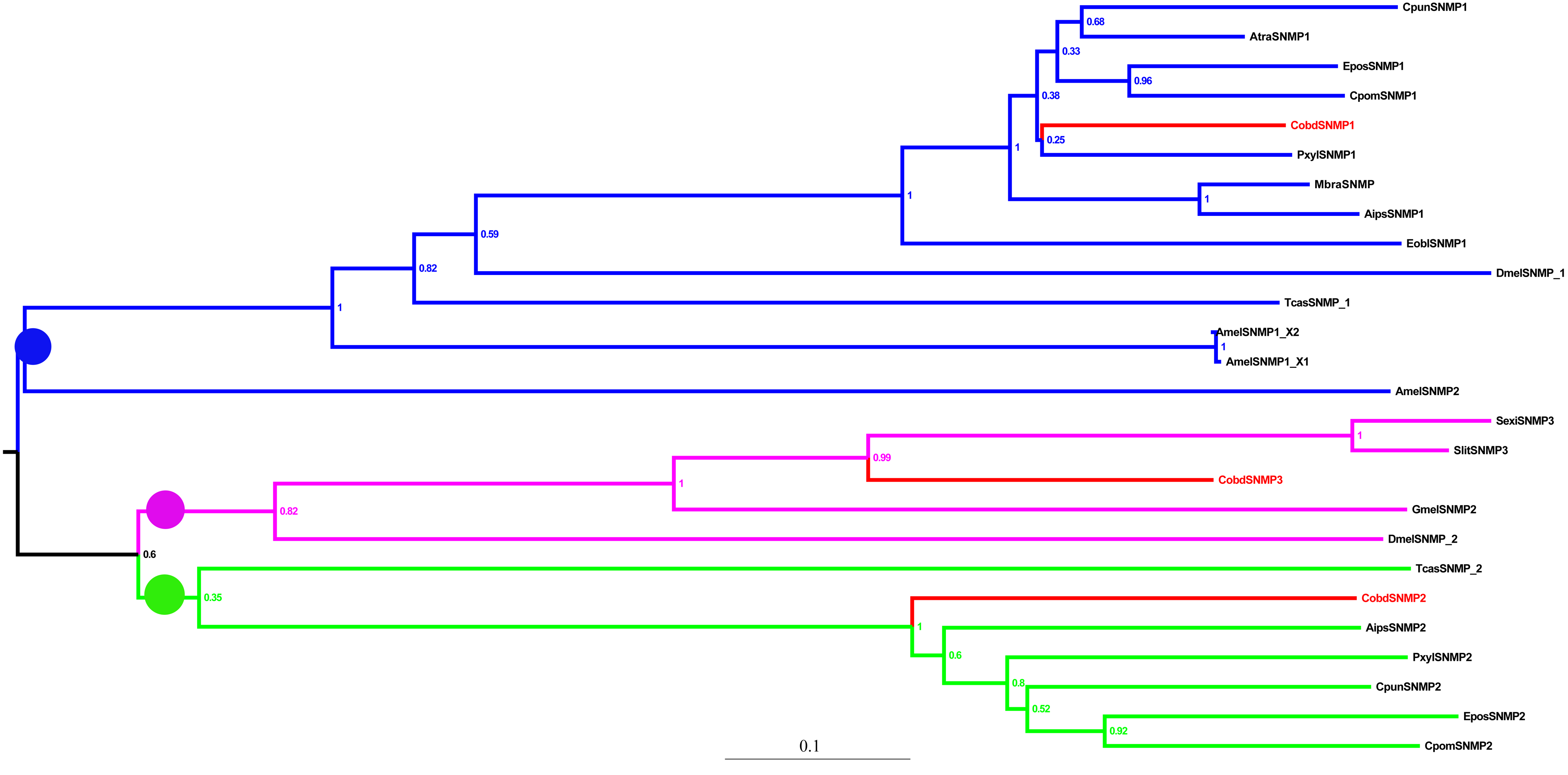

Supplement: Supplemental Information 6 — The NJ phylogenetic analysis of SNMPs of C. obducta (CobdSNMP, red) was performed with reference SNMPs of insect inNCBI database. The blue, green and black clade refer to SNMP1, SNMP2 and SNMP3 respectively. The scale bar represents 0.1 substitutions per site. [file peerj-08-8902-s006.png]

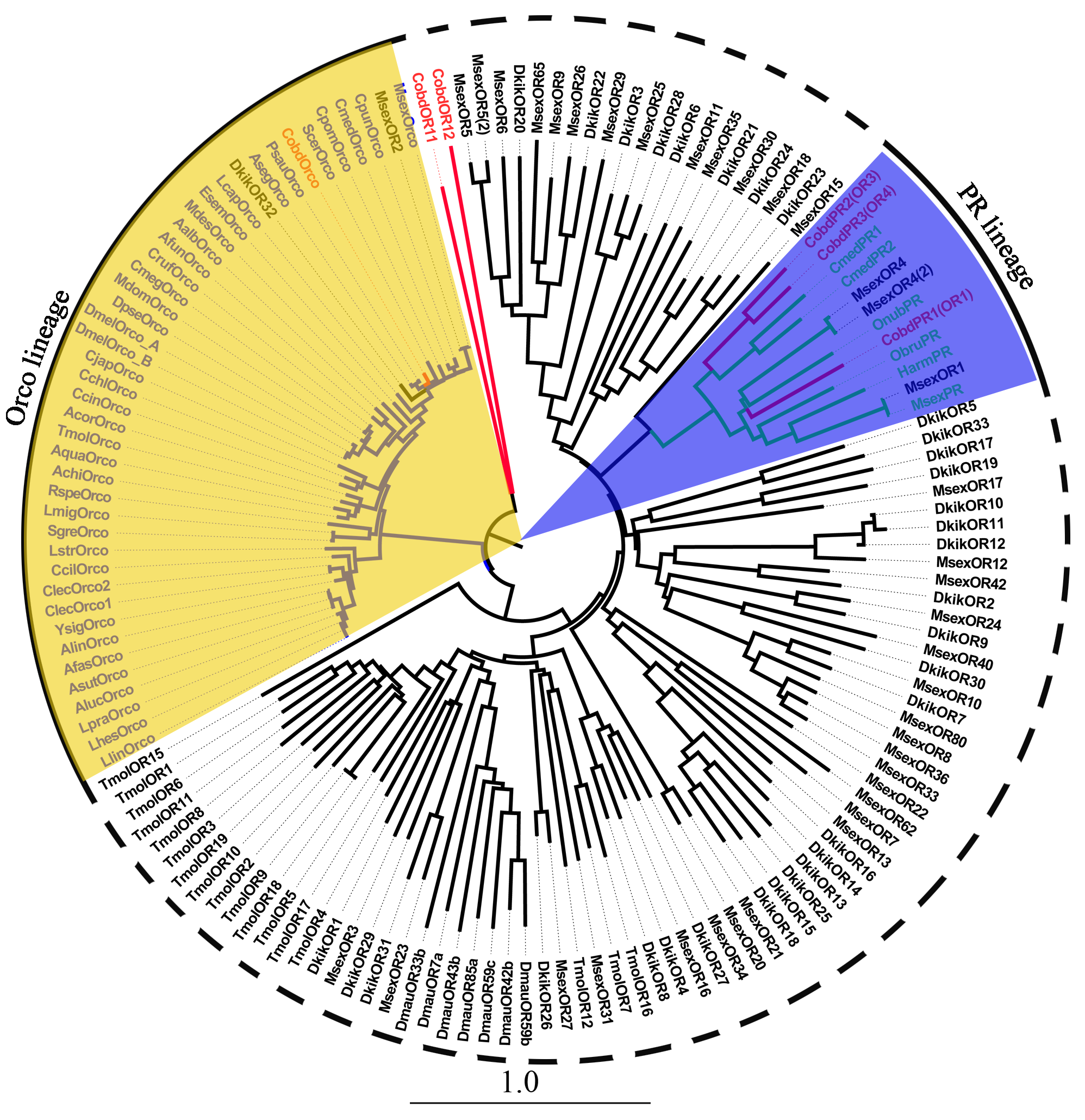

Supplement: Supplemental Information 7 — The NJ phylogenetic analysis of ORs of C. obducta (CobdOR, red) was performed with reference ORs of Lepidoptera (black) and Coleoptera (celeste) insect. The blue and yellow fill area refer to PR and Orco lineage respectively. The scale bar represents 1.0 substitutions per site. [file peerj-08-8902-s007.png]
