# Supplementary material for: The whole body transcriptome of Coleophora obducta reveals important olfactory proteins
Source: PeerJ. 2020 Apr 10;8:e8902. doi: 10.7717/peerj.8902 (PMC7153557; doi:10.7717/peerj.8902)
Supplement: Supplemental Information 8 [file peerj-08-8902-s008.docx]

**The whole body transcriptome of *Coleophora obducta* reveals important olfactory proteins**

Dongbai Wang^2^, Jing Tao^3^, Pengfei Lu^3^, Youqing Luo^3^, Ping Hu^1,2^

^1^ Guangxi University, Nanning, Guangxi, China

^2^ Xingan Vocational and Technical College, Xinganmeng, Inner mongolia, China

^3^ Beijing Key Laboratory for Forest Pest Control, Beijing Forestry University, Beijing, China

**Supplementary file 8 Identified olfactory proteins and quality index of antennal and whole body transcriptome in manuscript**

| **Quality indexs** | ***Coleophora obducta*** | ***Oedaleus infernalis*** | ***Batocera horsfieldi*** | ***Mythimna separata*** | ***Episyrphus balteatus*** | ***Eupeodes corollae*** | ***Tessaratoma papillosa*** |
| --- | --- | --- | --- | --- | --- | --- | --- |
| **Tissues** | whole bodyof male and female adult | whole body of male and female adult | male, female  larval, pupal | antenna | antenna | antenna | antenna |
| **Raw reads** | 45,859,325 | 115,294,434 | 50510255 | 82,290,798 | 68,710,000.00 | 77,280,000.00 | 48,712,716 |
| **Clean reads** | 45,337,446 | 113,315,306 | 48668786 | 77,734,418 | 65,690,000.00 | 74,250,000.00 | 48,630,777 |
| **Q20(%)** | 97.57 | 96.79% | 96.26 | 98.29% | \ | \ | 96.24 |
| **Q30(%)** | 93.53 | \ | 90.58 | \ | \ | \ | 90.93 |
| **GC content(%)** | 47.86 | 44.67% | 41.3 | 43.68% | \ | \ | 34.09 |
| **Total transcripts number** | 96,657 | 113,810 | 171,664 | 123,094 | 53,575 | 50,942 | 74,183 |
| **Total unigenes number** | 52,354 | 92,476 | 877,32 | 62,779 | 54,116 | 61,220 | \ |
| **Largest length(bp)** | 19,273 | \ | 27920 | \ | \ | \ | 24,277 |
| **Average length(bp)** | 900 | 729 | 2048 | 503 | 889 | 1039 | 1096 |
| **N50** | 1533 | 1528 | 3669 | 734 | 1724 | 2104 | 2342 |
| **OBPs** | 16 | 18 | 7 | 32 | 49 | 44 | 33 |
| **CSPs** | 14 | \ | 3 | 16 | 6 | 7 | \ |
| **Ors** | 11 | \ | \ | 71 | 51 | 42 | 59 |
| **GRs** | 10 | \ | \ | 1 | 14 | 16 | \ |
| **IRs** | 7 | \ | \ | 8 | 32 | 23 | 14 |
| **SNMPs** | 3 | \ | \ | 2 | 2 | 2 | \ |
| **ODEs** | 6 | \ | \ | \ | \ | \ | \ |
